# Spectral dependence as a framework for neural coordination

**DOI:** 10.1016/j.crneur.2026.100159

**Published:** 2026-05-11

**Authors:** C. Besosa, Y. Qin, S.N. Burke, A.P. Maurer

**Affiliations:** a McKnight Brain Institute, Department of Neuroscience, University of Florida, Gainesville, FL, 32610, USA; b Engineering School of Sustainable Infrastructure and Environment, University of Florida, Gainesville, FL, 32611, USA; c Department of Neuroscience, University of Arizona, Tucson, AZ, USA; d BIO5 Institute, University of Arizona, Tucson, AZ, 85719, USA

**Keywords:** Cognition, Gamma, Hippocampus, Local field potential, Rhythm, Theta

## Abstract

Theories of large-scale neural coordination frequently assign distinct cognitive functions to discrete, independent frequency bands of oscillatory activity. In the hippocampus, memory encoding and recall are attributed to specific non-overlapping gamma frequencies relayed through separate anatomical pathways. These “spectral parcellation” models assume frequency bands operate as functionally meaningful units that must be selectively generated, maintained, and decoded. However, this framework is inconsistent with biophysical constraints governing synaptic integration, current flow, and excitatory–inhibitory balance. An alternative framework treats oscillatory structure as an emergent consequence of multiscale circuit dynamics under shared constraints. Slow, large-amplitude oscillations such as theta (4–12 Hz) organize inter-regional coordination while higher frequencies reflect local dissipation of synaptic energy through progressively smaller, faster circuit motifs. Cross-frequency coupling arises naturally from this energy redistribution rather than from dedicated multiplexing mechanisms. This “energy cascade” framework makes explicit, falsifiable predictions about how spectral structure scales with behavioral state, metabolic demand, and experimental perturbation. We review evidence from hippocampal theta-gamma interactions demonstrating that: (1) gamma power covaries with theta power, (2) gamma properties shift continuously rather than discretely with circuit state, and (3) perturbations propagate hierarchically across frequencies rather than selectively disrupting isolated bands. These findings support constraint-based models over frequency-parcellation accounts and suggest that oscillations reflect how circuits dissipate energy under physical constraints rather than serving as discrete communication channels.

## Highlights

• Theoretical foundation for understanding oscillatory mechanisms of cognition.
• Theta-gamma interactions as mechanism for inter-regional neural coordination.
• Systematic integration of biophysical constraints with cognitive theory.
• Compare spectral parcellation models to spectral dependence.

## Graphical abstract

Caption. Neural coordination emerges from a hierarchical energy cascade, where macroscale reentrant loops (blue) drive mesoscale reverberating circuits (green) and microscale synaptic dissipation (red). This multiscale architecture produces a unified, continuous spectral signature (right), indicating that brain rhythms are interdependent biophysical regimes rather than functionally isolated frequency bands.

**Figure:**
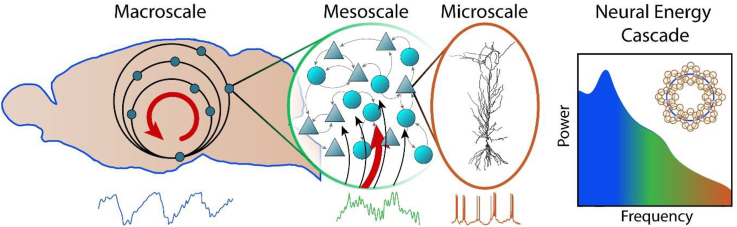

## 1 Introduction

How neural activity is coordinated across different brain regions to enable behavior remains a central question in neuroscience (Buzsaki and Draguhn, 2004; Eichenbaum, 2000; McNaughton et al., 2003; Sirota et al., 2003; Sutherland and McNaughton, 2000; Teyler and DiScenna, 1986). While the cortex exhibits sensorimotor biases, segregating higher cognition by anatomy alone echoing early phrenological mapping attempts (Gall, 1822-1825; Simpson, 2005), adaptive behavior relies on sequential interactions across cortical areas rather than isolated regions (Felleman and Van Essen, 1991; Hebb, 1949, 1958; Varela et al., 2001). For instance, object recognition requires integrating visual features processed in circuits separated by large distances. The brain achieves this integration across viewpoints and modalities (Barense et al., 2010; Devlin and Price, 2007; DiCarlo et al., 2012; Jacklin et al., 2016; Reid et al., 2012; Winters and Reid, 2010). This challenge is often labeled the binding problem, though it can be more precisely framed as a problem of dynamic convergence under anatomical and temporal constraints.

To explain such coordination, many theories rely on spectral binning and parcellation, in which cognitive functions are attributed to discrete frequency bands. These frameworks filter field potentials into narrow frequency windows, then relate phase or amplitude coupling to behavioral states, commonly focusing on hippocampal theta (4–7 Hz) and gamma (30–100 Hz) rhythms (Buzsáki, 2005; Colgin, 2016; Headley and Paré, 2017; Lisman and Jensen, 2013). The relationship between gamma and psychological function has historical precedence in theories that gamma synchronization binds an object's features across the cortex to form a coherent percept or experience (Engel et al., 2001; Singer and Gray, 1995). Contemporary frameworks further subdivide gamma into slow (30–50 Hz) and fast (60–100 Hz) variants, with proposed functional mappings to distinct anatomical pathways (CA3→CA1 versus entorhinal→CA1) and cognitive operations (retrieval versus encoding) (Bieri et al., 2014; Colgin et al., 2009; Fernández-Ruiz et al., 2021; Wang et al., 2025). These mappings form a testable set of predictions that can be evaluated against biophysical constraints and empirical evidence.

An alternate class of theories contends that the full frequency spectrum detected in LFP signals is interdependent, with large-amplitude slow rhythms driving activity in lower-amplitude fast rhythms (Buzsaki, 2006; Sheremet et al., 2018, 2019; Zhou et al., 2022). As Buzsaki and Draguhn (2004) synthesized:

“… perturbations occurring at slow frequencies can cause a cascade of energy dissipation at higher frequencies (Bak et al., 1987) and that widespread slow oscillations modulate faster local events (Csicsvari et al., 2003; Sirota et al., 2003; Steriade, 2001). These properties of neuronal oscillators are the result of the physical architecture of neuronal networks and the limited speed of neuronal communication due to axon conduction and synaptic delays (Nunez, 1995). Because most neuronal connections are local (Braitenberg and Schüz, 2013), the period of oscillation is constrained by the size of the neuronal pool engaged in a given cycle. Higher frequency oscillations are confined to a small neuronal space, whereas very large networks are recruited during slow oscillations (Csicsvari et al., 2003; Steriade, 2001). These relations between anatomical architecture and oscillatory patterns allow brain operations to be carried out simultaneously at multiple temporal and spatial scales (Buzsáki et al., 2004; Buzsaki and Draguhn, 2004)

Buzsáki's recent synthesis of subjective time and brain rhythms reaffirms this cascade architecture (Buzsáki, 2026). In this view, oscillations arise as scale-dependent consequences of circuit architecture and biophysical constraints, organized into a nested hierarchy. Frequencies reflect circuit size, conduction delays, and inhibition, not task-specific functions. Buzsáki explicitly rejects modular clock-like interpretations, describing classical timing models as phrenological and noting that no such clock was found. Oscillations are therefore not independent channels operating in parallel, but hierarchically nested processes coupled through cross-frequency phase-amplitude relationships that impose relative temporal structure across scales.

**Fig. 1:**
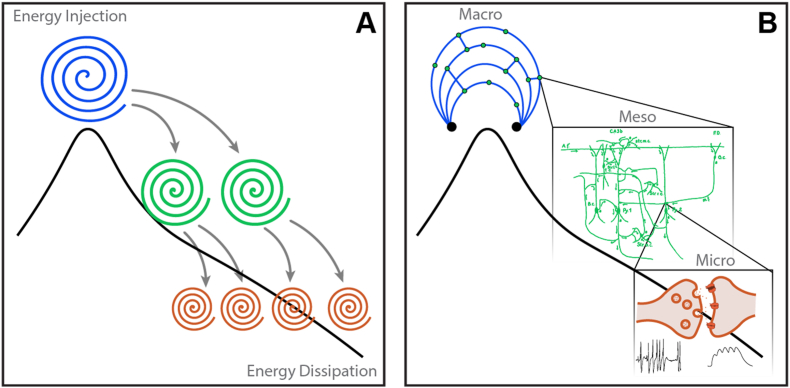
**The Energy Cascade. (A) Physical constraints of a driven, dissipative system.** Energy is injected at large spatial and temporal scales and redistributed through nested, reentrant loops (Richardson, 1922). This hierarchical redistribution is summarized by the power spectral density (PSD; black curve), whose downward slope reflects continuous transfer of activity across scales. The PSD does not generate this process but provides a descriptive signature of scale-dependent energy flow. The cascade terminates at dissipation-dominated scales, where organized dynamics can no longer sustain coherent structure and further transfer ceases. **(B) Neural instantiation of the cascade.** The spectral slope (black curve) emerges from a hierarchy of reentrant architectures. Macro-scale loops (blue), shaped by long conduction delays and extended anatomical paths (Buzsaki and Draguhn, 2004; Edelman, 1987), constrain dynamics toward slow, large-scale timescales (e.g., theta-range activity). These loops, in turn, drive meso-scale reverberating circuits (green), first described by Lorente de Nó (1938), which redistribute excitation into progressively smaller motifs. At micro-scales (orange), including synaptic transmission, high-frequency presynaptic spiking is transformed into subthreshold postsynaptic depolarization without further propagation. This conversion of structured activity into local, non-propagating signals represents one concrete mechanism of dissipation in neural tissue. From this perspective, conventional spectral bands (e.g., theta, gamma) do not correspond to discrete biological modules but reflect interdependent regimes of a single multiscale, driven, dissipative process.

This hierarchical framework remains continuous with Buzsáki's earlier formulation, in which oscillations reflect a cascade of energy dissipation at higher frequencies (Buzsaki and Draguhn, 2004). The oscillatory hierarchy spans orders of magnitude and provides relative divisions that serve as internal units without invoking absolute metrics. Within this cascade architecture, a mechanistic consequence follows. As activity propagates to progressively smaller spatial scales, anatomical constraints impose limits on recruitment. At sufficiently small scales, where loop sizes can no longer sustain organized reentry, structured dynamics must terminate. Energy injected at large scales redistributes through nested loops until, at microscales, synaptic transmission becomes subthreshold and coherent structure dissolves (Fig. 1).

By the nature of this organization, standard spectral parcellations (delta, theta, alpha, beta, gamma) are divisions that primarily reflect the neuroscientist's taxonomy rather than discrete biological entities. If naming brain regions and assigning each a singular cognitive function is anatomical phrenology, then isolating frequency bands to do the same is a form of spectral phrenology. In a driven, dissipative biological system, these bands are interdependent components of a multiscale process. The divisions are descriptive of scale: large, slow rhythms describe global dynamics, whereas smaller, faster rhythms describe more local dynamics.

This coupling of loop size and rhythmic structure is also consistent with reentrant architectures. Edelman noted that phasic reentry among parallel systems contributes to rhythmic activity (Edelman, 1987), and Edelman and Gally (2013) later formalized coordination through reentrant organization. Under this framework, macroscale rhythms are frequently observed as traveling waves (Muller et al., 2018). Rhythmic activity is therefore better described as nested dynamics across loop sizes than as a frequency band acting as a discrete functional unit.

The energy cascade framework also builds on a principle that is increasingly supported anatomically: the brain's geometry is scale-invariant over a wide range of spatial scales. The hippocampal formation exhibits fractal-like organization (Gutierrez Aceves et al., 2018), consistent with fractal cortical geometry more broadly (Krohn et al., 2026; Wang et al., 2024). This substrate constrains the dynamics that can emerge. The LFP power spectrum, with its characteristic 1/f^α scaling, may reflect energy propagation through this substrate, with dissipation occurring through mechanisms including probabilistic synaptic transmission (Lisman, 1997) and dendritic filtering.

Broadly, a neural energy cascade can be summarized by modifying the Lewis Fry Richardson poem (1922):

**Big loops have nested loopsThat redistribute excitation,And nested loops have smaller loopsAnd so on to dissipation.**

This view differs from spectral parcellation by grounding coordination in circuit architecture and conduction delays rather than assigned frequency channels. It directly challenges claims that oscillations, particularly in the gamma range, function as dedicated channels for specific cognitive tasks (Kopell et al., 2000; Siegel et al., 2012). Instead, biophysically grounded approaches (Buzsaki et al., 2012; Einevoll et al., 2013) provide a framework for energy cascade accounts (Buzsaki, 2006; Buzsáki and Mizuseki, 2014) and for binding-by-rate-enhancement (Roelfsema, 2023; Shadlen and Movshon, 1999), in which rhythmic structure is a consequence of architecture rather than a programmatic command.

While theories of coordination are often framed as general cortical principles, their evaluation rests on biophysical constraints conserved across regions. Evolution has repeatedly reused the same fundamental cellular and circuit mechanisms, including excitatory drive, inhibitory feedback, current conservation, and membrane filtering, rather than inventing region-specific coding schemes. The hippocampus is therefore not treated here as a system with unique governing principles, but as one in which shared constraints are unusually transparent. Its laminated inputs, well-characterized inhibitory circuitry, and prominent behaviorally modulated rhythms allow precise linkage between anatomy, biophysics, and extracellular field measurements. Accordingly, this review focuses on hippocampal physiology to test the mechanistic plausibility of spectral parcellation versus spectral dependence, while drawing on examples from other regions. In the final section, we discuss how the same constraints are expected to shape oscillatory structure in neocortex, where increased architectural complexity may obscure the linkage between anatomy and field measurements without altering the underlying principles.

This review examines competing theories of how brain oscillations coordinate cognition (Table 1). Spectral independence proposes that discrete frequency bands in the local field potential are assigned to unique cognitive functions or anatomical pathways. Spectral dependence contends that the spectrum is interconnected. We focus on the biophysical constraints that determine which claims are mechanistically plausible.

**Table 1:** Theoretical models of oscillations and neural coordination.

Name	Core claim	Framework	Key citations
**Binding by Synchrony (BBS)**	Features of a perceptual object are bound when neurons encoding those features exhibit temporally precise synchronous firing, such that synchrony itself serves as the binding mechanism.	Spectral parcellation and independence	Singer and Gray (1995)
**Communication Through Coherence (CTC)**	Effective inter-regional communication requires sustained phase alignment between oscillatory activity in sender and receiver populations, enabling selective transmission through coherence-dependent gain.	Spectral parcellation and independence	Fries (2005), 2015
**Spectral Fingerprints**	Cognitive states and large-scale network configurations are organized by frequency-specific patterns of inter-regional coherence, with different bands indexing distinct functional operations.	Spectral parcellation and independence	Siegel et al. (2012)
**Spectral Multiplexing**	Discrete oscillatory frequency bands act as partially separable communication channels, allowing simultaneous routing of multiple information streams through frequency-specific segregation and decoding.	Spectral parcellation and independence	Akam and Kullmann (2014)
**Energy Cascade**	Oscillatory structure reflects scale-dependent redistribution of activity from large, slow circuits to smaller, faster motifs, governed by anatomy, conduction delays, and dissipation rather than by frequency-specific function.	Spectral dependence	Buzsáki et al., 2004; Sheremet et al. (2018)
**Binding by Rate Enhancement (BBRE)**	Feature integration arises when neurons participating in the same object representation exhibit increased firing rates relative to other populations, with enhancement propagating through recurrent circuitry without requiring precise spike timing or frequency-specific channels.	Spectral dependence	Roelfsema (2023)

**Note.** Spectral parcellation models treat frequency bands as functionally meaningful units that must be selectively generated, maintained, and decoded. Spectral dependence models instead treat oscillatory structure as an emergent consequence of multiscale circuit dynamics under shared biophysical constraints.

## 2 Historical perspectives on the role of gamma rhythms in cognition

Ranck (1973) emphasized that learning situations engage multiple brain systems beyond those typically measured by experimenters. Even in simple conditioning paradigms, animals may form complex associations that include spatial hypotheses and social interpretations. Recording from visual cortex during a tone–shock avoidance task might reveal neural changes related to learned gaze patterns, but such changes represent only one component of a more complex learning process involving multiple interacting systems (O'Keefe and Nadel, 1978). Ranck argued that the complexity of experimental analysis should match the scope of the brain systems underlying behavior rather than the apparent simplicity of the observed behavior itself. This historical perspective applies directly to contemporary oscillation research, where frequency bands are often studied in isolation rather than as consequences of interactions among distributed circuits operating across spatial and temporal scales.

Early characterizations of gamma oscillations in hippocampal and entorhinal circuits provided foundational insights into the network conditions under which these rhythms emerge (Chrobak and Buzsáki, 1998; Chrobak et al., 2000). Buzsáki and Chrobak (1995) identified interneurons as scaffolds supporting the temporal organization of hippocampal ensembles, demonstrating that gamma rhythms arise naturally from network architecture and biophysical constraints. This perspective framed gamma as an emergent rhythm of relatively small circuits with appropriate excitatory–inhibitory balance and preceded later efforts to assign discrete cognitive functions to gamma sub-bands.

Subsequent work further demonstrated that gamma oscillations arise from well-defined circuit interactions and state-dependent network configurations. Gamma rhythms readily emerge in hippocampal slices through local circuitry alone (Fisahn et al., 1998; Whittington et al., 2000), whereas theta-like oscillations observed in vitro require strong cholinergic activation and likely reflect resonance or induced rhythmicity rather than spontaneously generated behavioral theta (Fischer et al., 2002). These experimentally induced rhythms differ from typical in vivo theta in their spatiotemporal dynamics and functional specificity. Gamma, therefore, arises naturally from intrinsic microcircuit dynamics governed by local excitatory–inhibitory balance, synaptic time constants, and short conduction delays, whereas genuine theta typically reflects broader, multi-region coordination. This same dependence on local input strength and circuit geometry is well established and imposes a fundamental constraint on interpretation. When frequency and amplitude reflect local circuit properties (excitatory drive strength, inhibitory feedback timing, and synaptic time constants) they cannot simultaneously serve as independently programmable channels for routing information.

The view of gamma as a signature of excitatory–inhibitory volleys in local networks has been increasingly supplemented by contemporary theories proposing that gamma rhythms coordinate disparate brain regions or route neural activity between distinct anatomical pathways. Our concern is not that frequency-parcellation models ignore microcircuit mechanisms, but that they elevate oscillatory frequency itself to a functional agent of communication, rather than treating rhythmic structure as an emergent consequence of how local excitatory–inhibitory circuits redistribute and dissipate synaptic drive.

## 3 Theoretical frameworks for models of spectral parcellation

This section explores a prominent class of theories developed to explain how the brain solves the “binding problem” (Table 1). These “spectral parcellation” models propose that specific brain oscillations coordinate neural processes to support cognition. While initially focused on perception, these ideas were later extended to other domains such as memory (Colgin, 2015; Dvorak et al., 2018; Fernandez-Ruiz et al., 2023; Fernández-Ruiz et al., 2021) and behavioral flexibility (Cho et al., 2023; Sohal et al., 2009). These frameworks vary in the strength of their claims. Some propose that oscillatory frequency bands play a direct causal role in routing or gating information transfer between circuits. Others adopt a more modest position, treating band-limited activity as a proxy for pathway engagement without asserting that oscillations themselves mediate communication. Both levels of interpretation, however, share a common foundational requirement: that the spectral components in question are separable from the underlying broadband and harmonic structure of the dominant rhythm. The biophysical evidence reviewed below challenges this shared assumption. We review these influential models here, highlighting the limitations and biophysical constraints that challenge their feasibility.

### 3.1 Binding by synchrony

Milner (1974) was among the first to propose visual binding through neural synchrony, noting:

*'If adjacent, or nearly adjacent, cells interact when excited, in such a way as to synchronize and perhaps intensify each other's activity, this could provide the unifying characteristic that ties the elements of a figure together …*. *The resulting difference in the effectiveness of temporal summation could serve to funnel the signals from different objects in the field into different paths through the feature detection network and thus minimize their mutual interference.’* (p.526).

Since this early conjecture, the visual cortex has been a primary target for studying gamma oscillations, based on the assumption that perception requires integrating stimulus elements that activate distinct cortical areas. Gray and Singer (1989) reported that neurons in the cat visual cortex exhibited synchronous firing in the gamma range (30-60 Hz) when responding to coherent stimuli. This finding formed the basis for the Binding by Synchrony (BBS) hypothesis (Singer and Gray, 1995), which proposes that gamma synchronization (30-100 Hz) between different brain areas binds features into a coherent percept (Engel et al., 2001).

In support of this, studies have reported increased gamma synchrony during perceptual tasks (Gray et al., 1989; Khamechian et al., 2019; Rodriguez et al., 1999). These ideas led to influential reviews (Engel et al., 2001; Tallon-Baudry and Bertrand, 1999) and the evolution of related theories, such as Communication Through Coherence (Fries, 2005, 2015). Yet critics note these observations are largely correlational; synchrony might be a consequence, not a cause, of integration (Shadlen and Movshon, 1999). Some research finds perceptual grouping independent of synchrony strength (Roelfsema et al., 2004).

At this point, it is worth recalling the historical contingency of the term *gamma* itself. As described by Buzsáki (2025), the label “gamma” was not introduced to denote a specific computational or cognitive mechanism but emerged as a convenient name for fast field activity after alpha and beta had already been coined (Bressler and Freeman, 1980; Jasper and Andrews, 1938). Early mechanistic work established the central role of inhibition in generating fast rhythms (Buzsáki et al., 1983; Penttonen et al., 1998), often without using the term “gamma” at all. Buzsáki later emphasized that gamma refers to a range of activity rather than a fixed frequency, and that its boundaries were never intended to mark discrete functional entities. This historical context clarifies a central concern for binding-by-synchrony models: gamma-band activity reflects circuit-level constraints on fast dynamics, not an independently selectable communication channel.

Significant limitations to the BBS model have since emerged. First, gamma oscillations in sensory cortex are highly dependent on stimulus properties. Henrie and Shapley (2005) demonstrated that gamma power and coherence in macaque V1 vary strongly with stimulus contrast and size, challenging the idea that gamma provides a general-purpose mechanism independent of sensory conditions. Similar stimulus dependence has been reported in subsequent work (Ray and Maunsell, 2010).

Second, concerns about generalizability remain. Gamma oscillations are robust in cats and some primates, yet they appear weaker and less consistent in humans (Hermes et al., 2015), raising questions about the extent to which findings from select animal models generalize across species.

A third major limitation involves ecological validity. Much foundational evidence for BBS was obtained under constrained conditions, including anesthetized preparations and artificial stimuli such as drifting gratings (Eckhorn et al., 1988; Gray et al., 1989). Under naturalistic conditions in awake animals, gamma synchrony is often weak, intermittent, or absent (Gieselmann and Thiele, 2008; Kayser et al., 2003; Thiele and Stoner, 2003) questioning whether precise synchronization reflects a general computational principle or a state-dependent regime induced by specific experimental contexts.

Interpretation of gamma synchrony is further complicated by methodological issues. High-frequency LFP components are often contaminated by broadband, spike-related signals (Jia et al., 2013; Ray and Maunsell, 2010). Because BBS requires identifiable, narrowband oscillations with precise phase relationships, such contamination complicates the isolation of the empirical phenomenon on which the theory relies.

Finally, causal evidence for gamma's role in perception remains limited. Histed and Maunsell (2014) demonstrated that optogenetically inducing gamma-frequency activity in macaque V1 increased spectral power but failed to improve, and in some cases disrupted, visual detection performance. While externally induced gamma may differ from endogenous rhythms, these findings highlight that replicating gamma spectral signatures is insufficient to invoke functional engagement. This underscores the necessity for caution when interpreting gamma activity as a causal driver of visual perception.

### 3.2 Communication Through Coherence (CTC)

Similar to the Binding by Synchrony model, the Communication Through Coherence (CTC) hypothesis (Fries, 2005, 2015) posits that synchrony between brain regions is not epiphenomenal and interprets coherence between oscillatory phases as enhancing neuronal communication. Whereas BBS was primarily discussed in the context of visual perception, CTC generalizes this framework to propose a mechanism for coordinating activity and routing information across networks and behavioral contexts.

The core mechanism proposed by CTC rests on the observation that neuronal oscillations reflect rhythmic fluctuations in the excitability of local populations. These oscillations produce alternating periods of relatively high excitability (high gain) and reduced excitability (low gain). Within this framework, effective communication requires temporal alignment: synaptic inputs must arrive at the efferent targets during a high-excitability phase. Coherence, defined as a consistent phase relationship between oscillatory activity in two regions, is taken to indicate such alignment and interpreted as maximizing the impact of synaptic input (Fries, 2015). This interpretation provides a putative mechanism for selective communication, in which synchronized pathways are rendered effective while non-coherent inputs, arriving out of sync with more excitable phases, are comparatively attenuated (Akam and Kullmann, 2012).

CTC emphasizes phase alignment as a means of modulating effective connectivity, a process often invoked in accounts of selective attention. Empirical support for this view comes primarily from studies reporting correlations between inter-areal coherence and behavioral performance (Bosman et al., 2012; Womelsdorf et al., 2007). In primates, directing attention to a specific stimulus has been associated with increased gamma-band coherence between visual areas such as V1 and V4 that process the attended stimulus (Bosman et al., 2012). Similar task-related coupling has been reported between prefrontal cortex and visual areas during attentional demands (Gregoriou et al., 2009).

Extending this logic, CTC further proposes that information routing is organized across distinct frequency bands, implying a spectral segregation of influence. In this view, gamma rhythms (approximately 30–90 Hz) are commonly interpreted as mediating feedforward or “bottom-up” sensory signaling, whereas lower-frequency bands, such as alpha (8–12 Hz) and beta (13–30 Hz), are associated with feedback or “top-down” control (Bastos and Schoffelen, 2015; Richter et al., 2017). In the hippocampus, for example, slow- and fast-gamma activity has been interpreted as differentially coupling CA1 to CA3 and entorhinal inputs, respectively, and thus as routing information related to memory retrieval versus encoding (Colgin et al., 2009).

Despite its conceptual appeal, establishing causality within the CTC framework remains challenging, as most supporting evidence is correlational. Recent work instead indicates that *coherence may arise as a consequence of inter-areal communication rather than serving as its causal mechanism*(Schneider et al., 2021). Using simultaneous recordings in macaque fronto-parietal cortex and mouse LGN–V1, combined with optogenetic perturbations and computational modeling, Schneider et al. demonstrated that inter-regional coherence emerges naturally when spikes from an efferent population of neurons elicit synaptic potentials both locally and in anatomically connected target regions. Crucially, optogenetic silencing of spiking activity in the target area did not abolish coherence, indicating that spike entrainment in the receiver is not required (for similar evidence in the hippocampus, see Zhao et al., 2026). Instead, afferent synaptic input was identified as the principal determinant of coherence.

These results imply that large-scale coherence patterns arise from anatomical structure and afferent input dynamics that continuously reconfigure with behavioral state. Consistent with this interpretation, coherence values are often low even during strong inter-regional interactions (Ray and Maunsell, 2010), and attention-related increases in coherence are confounded by concurrent changes in firing rates, stimulus drive, and phase-locking properties of projection neurons, each of which can modulate coherence independently of synaptic efficacy (Schneider et al., 2021). Together, these findings do not rule out coordinated oscillatory activity across brain regions, but they substantially constrain interpretations that treat coherence itself as a causal control variable for inter-regional communication.

### 3.3 The spectral fingerprints model

An extension of the BBS and CTC frameworks proposes that cognition is supported by “spectral fingerprints,” defined as frequency-specific patterns of inter-regional coherence and coupling that organize large-scale brain networks (Siegel et al., 2012). Consistent with earlier parcellation models, this account contends that distinct frequency bands serve complementary functions, with gamma-band synchrony associated with feedforward sensory processing and lower-frequency rhythms such as alpha, beta, and theta associated with top-down influences, attention, and decision making. Within this framework, oscillatory coherence is interpreted as providing a flexible frequency-based code for coordinating activity across distributed cortical and subcortical circuits.

As with earlier spectral parcellation approaches, this framework presupposes that frequency bands can serve as stable functional identifiers across contexts, despite constraints imposed by circuit geometry, conduction delays, and shared generators. Although spectral fingerprinting has been influential in linking oscillatory activity to computational roles, it has been criticized as overly reductive, given that oscillations are highly context-dependent and the same frequency band can support multiple functions across tasks and brain regions (Buzsáki and Schomburg, 2015; Buzsáki and Watson, 2012; Ray and Maunsell, 2015). Methodological concerns further complicate interpretation, as coherence and coupling measures are vulnerable to artifacts such as volume conduction and common input, which can spuriously inflate frequency-specific interactions (Bastos and Schoffelen, 2015; Vinck et al., 2011).

Moreover, much of the supporting evidence remains correlational, making it difficult to establish whether frequency-specific coherence plays a causal role in cognition (Herrmann et al., 2016; Jazayeri and Afraz, 2017). Critics have also noted that the framework underemphasizes cross-frequency interactions (Aru et al., 2015; Canolty and Knight, 2010) and transient dynamics. Short-lived oscillatory bursts, which dominate many cognitive tasks, challenge the assumption that stable, band-limited signatures can serve as functional primitives and instead point toward time-varying, state-dependent dynamics as the basis for large-scale coordination (Lundqvist et al., 2016; van Ede et al., 2018).

### 3.4 Spectral multiplexing model

The Spectral Multiplexing Theory extends spectral parcellation by proposing that discrete frequency bands are independent communication channels that can be identified via frequency-selective filtering. Within this framework, slower rhythms, typically theta (4–8 Hz), are proposed to organize temporal windows within which faster oscillations, such as gamma (30–100 Hz), encode stimulus-specific information (Bieri et al., 2014; Lisman and Jensen, 2013). Related proposals suggest that distinct oscillatory frequencies may bias information flow from specific anatomical pathways, effectively routing input based on spectral content (Colgin et al., 2009).

While computational models demonstrate that resonance-based filtering in recurrent interneuron networks could, in principle, support frequency-selective transmission (Akam and Kullmann, 2010), several biophysical constraints challenge the plausibility and scalability of this mechanism in hippocampal circuits. CA1 pyramidal neurons exhibit theta-frequency resonance (Leung and Yu, 1998), whereas GABAergic interneurons typically show either low-frequency (1–3 Hz) or broad high-frequency (10–50 Hz) resonance profiles (Binini et al., 2021; Pike et al., 2000). The absence of stable, narrowband gamma resonance undermines a core requirement for precise frequency-selective filtering. Consistent with this constraint, hippocampal gamma activity is observed primarily as transient bursts nested within theta cycles rather than as sustained oscillations. Together, these properties challenge the assumption that gamma can function as an independent carrier frequency suitable for frequency-division multiplexing.

Spectral multiplexing is further constrained by the high-convergence architecture of hippocampal circuits. Routing accuracy in resonance-based models degrades as the number of simultaneously active inputs increases (Akam and Kullmann, 2010), a critical limitation for systems operating under dense convergence. Moreover, computational implementations of multiplexed routing typically require external manipulation of circuit parameters, such as synaptic weights or tonic drive, to switch routing states. In these models, gamma oscillations emerge from underlying state changes rather than initiating the routing process itself, indicating that gamma reflects the dynamic signature of a circuit's biophysical state rather than serving as a causal control variable for information flow.

Finally, apparent evidence for multiplexing may reflect limitations of signal processing rather than distinct neurophysiological coding schemes. Broadband spectral components and non-sinusoidal waveform shapes can generate spurious cross-frequency coupling in standard analytic methods (Aru et al., 2015; Cole and Voytek, 2017; Jones, 2016; Scheffer-Teixeira and Tort, 2016; Zhou et al., 2019, 2022). Under these conditions, apparent frequency segregation can arise without distinct generators, channels, or routing operations, further undermining the interpretation of multiplexing as a biologically instantiated communication strategy.

### 3.5 Hippocampal gamma as an exemplar of spectral parcellation models

**Fig. 2:**
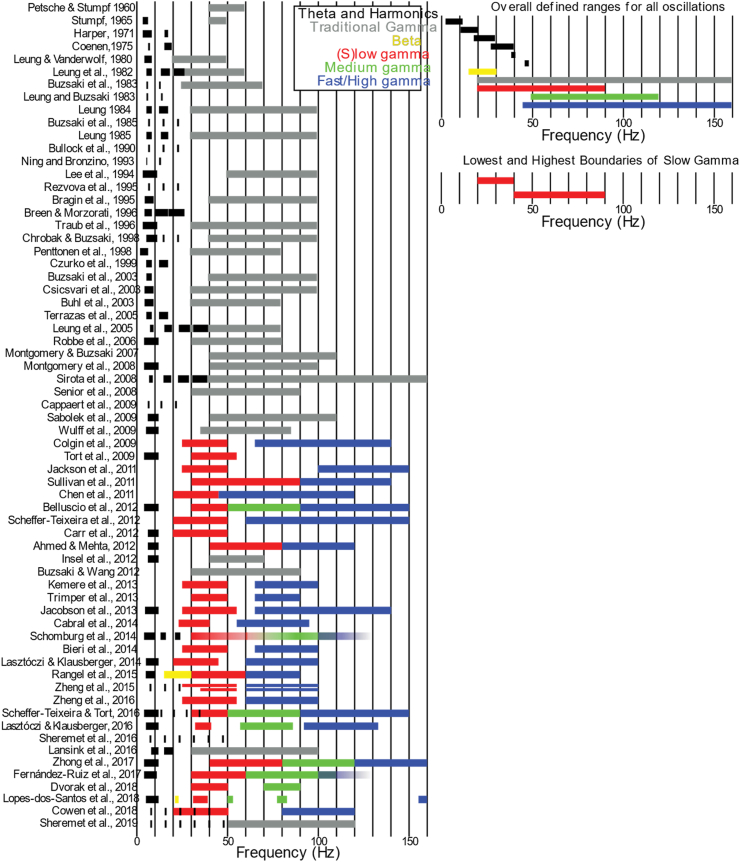
**Inconsistent frequency definitions across hippocampal oscillation studies. Left:** Historical definitions of medial temporal lobe oscillatory bands. Each bar shows the frequency range defined for a given oscillation in different studies. **Right top:** Combined defined ranges show theta harmonics overlapping the lower gamma band (<50 Hz), highlighting potential spectral confounds. **Right bottom:** The lowest and highest reported boundaries of slow gamma across studies show substantial variability, illustrating the absence of a single, invariant frequency definition for this rhythm. If slow gamma were a distinct biological entity with consistent generation mechanisms, frequency boundaries should converge across independent laboratories and recording conditions. Instead, definitional heterogeneity suggests that “slow gamma” may reflect analysis choices applied to continuous spectral structure rather than a discrete oscillatory process. Reproduced with permission from Zhou et al. (2019). Modified from the original to correct the representation of Lopes-Dos-Santos et al. (2018), who reported peak frequencies of ICA weight vectors (22, 35, 54, 80, and 169 Hz) rather than bounded frequency bands. These are plotted as narrow markers at their reported peaks because the paper defines no band boundaries. Of note, the component labeled ‘fast gamma’ (tSC5) peaks at 169 Hz, closer to the ripple band (135–250 Hz) than to any other study's definition of fast gamma, while the component labeled ‘beta’ (tSC1) at 22 Hz falls within the range dominated by theta harmonics.

The hippocampus has been a central testbed for spectral parcellation, especially models that assign distinct functional roles to “slow” and “fast” gamma during retrieval versus encoding. A fundamental problem immediately arises: the frequency boundaries used to define these bands vary substantially across studies (Fig. 2). Colgin et al. (2009) defined slow gamma as 25–50 Hz and fast gamma as 65–140 Hz; however, other reports use different cutoffs. This lack of agreement is not a cosmetic issue. If band definitions shift to accommodate each new dataset, the framework loses falsifiable boundaries and becomes difficult to refute. In parallel, the field often performs a kind of “dual phrenology,” mapping cognitive functions onto both frequency bands and anatomical subregions as if each mapping were independently diagnostic. The absence of a specified generation mechanism - for example, what synaptic, cellular, or circuit-level processes would enable CA3 to generate and transmit a discrete slow-gamma rhythm to CA1 - is what permits these definitions to drift across studies without apparent contradiction. A mechanistically anchored entity, defined by specific circuit properties, synaptic time constants, or interneuron populations, should predict convergent frequency boundaries across laboratories and recording conditions. Persistent definitional variability instead suggests that frequency labels accommodate correlational findings rather than track a stable biological process.

Colgin et al. (2009) proposed that slow gamma coordinates CA3→CA1 interactions during retrieval, whereas fast gamma reflects entorhinal→CA1 interactions during encoding. Subsequent work has not converged on a consistent anatomical and psychological mapping. In terms of the latter, Kemere et al. (2013) reported that power in both slow and fast gamma bands increased during exploration of novel environments relative to familiar ones, consistent with state-dependent modulation rather than frequency-specific encoding. Zheng et al. (2016) found that both frequency ranges could support either prospective or retrospective coding depending on task structure. Fernández-Ruiz et al. (2021) proposed a different anatomical mapping altogether, attributing lower-frequency gamma to lateral entorhinal inputs and object-related content and higher-frequency gamma to information streams related to space and medial entorhinal input. Although these accounts disagree about the upstream source, they share a critical assumption: that activity in the 30–50 Hz range reflects a separable oscillatory process rather than harmonic structure and nonlinear waveform features of theta that are partitioned by analysis choices.

A persistent problem is that identical frequency labels, particularly slow gamma, have been applied to activity attributed to different anatomical pathways (CA3→CA1 versus LEC→DG) without a mechanistic account of how multiple independent oscillatory generators with overlapping frequency ranges would arise from hippocampal circuitry. When considering that scientific conclusions should extend across manuscripts, the emerging evidence suggests a labeled-line continuity rather than independence. LEC inputs drive 30–50 Hz activity in dentate gyrus (Fernández-Ruiz et al., 2021), dentate gamma entrains CA3 (Hsiao et al., 2016), and CA3 gamma couples to CA1 (Colgin et al., 2009). This pattern is more consistent with the propagation of a circuit-level process through the pathway than with multiple functionally distinct gamma generators occupying the same frequency band. The alternative interpretation, that Fernández-Ruiz et al. identified a different slow gamma oscillator than Colgin et al. requires positing that hippocampal circuitry supports multiple independent yet overlapping 30–50 Hz generators with distinct anatomical origins and functional roles, a claim that lacks mechanistic support.

More fundamentally, functional claims about slow gamma make incompatible predictions. Kemere et al. (2013) found that slow gamma power decreases with running speed. If slow gamma mediates CA3→CA1 communication during retrieval, as proposed by Colgin et al. (2009), then animals should exhibit impaired CA3→CA1 interactions, disrupted place cell activity, and impaired recall at higher running speeds. No evidence has been reported to support this prediction. If slow gamma constitutes the mechanism enabling CA3 to drive CA1 spatial representations, then CA1 place cell activity should collapse when CA3 is silenced. However, complete CA3 silencing preserves both the fraction of CA1 place fields and their stable assembly expression (Zutshi et al., 2022), demonstrating that slow gamma cannot be the mechanism enabling CA3 to drive CA1 spatial coding.

This divergence illustrates a specific instance of a broader vulnerability identified by Buzsáki (2020), who critiqued top-down mappings between psychological constructs and neural correlates. The extension to frequency-band selection follows the same logic: Investigators can select whatever frequency range best correlates with a psychological label (retrieval, encoding, objects, space) and then back-map that range onto an anatomical pathway, producing a compelling story without identifying a generator. The result inherits the pitfalls of both anatomical and spectral phrenology. More fundamentally, the slow/fast dichotomy presupposes multiple distinct gamma oscillations in the hippocampus, an assumption that warrants scrutiny with respect to both the analytical tools used to identify and support theories of gamma routing and the anatomical and biophysical feasibility of these models.

### 3.6 Methodological limitations of spectral parcellation approaches

Theories of neural coordination based on spectral parcellation rely largely on correlational evidence and inverse inference, rather than direct mechanistic tests. A fundamental issue undermining gamma routing, BBS, CTC, and spectral multiplexing frameworks is the assumption that because spectral analysis can isolate distinct frequency bands, those bands correspond to independent cognitive or neural processes (Buzsaki, 2006; Jasper, 1948; Muthukumaraswamy and Singh, 2011). This inference conflates analytic decomposition with physiological separability. Neural circuits generate complex, non-sinusoidal activity patterns that distribute power across multiple frequencies simultaneously. As a result, spectral components may reflect different projections of a single underlying dynamic rather than distinct oscillatory generators (Canolty and Knight, 2010; Cohen, 2017).

Optogenetic and chemogenetic perturbations have sometimes been interpreted as causal evidence for frequency-specific routing (Liu et al., 2023; Yamamoto et al., 2014). However, such manipulations conflate pathway necessity with frequency necessity. Suppressing or driving specific inputs alters synaptic gain, spike timing statistics, and population synchrony upstream of the recorded site, necessarily reshaping downstream field potentials. For example, chemogenetic suppression of dentate gyrus and CA3 parvalbumin-positive versus somatostatin-positive interneurons produces opposing shifts in CA1 gamma-range power, with PV + suppression enhancing lower-frequency gamma components and SST + suppression enhancing higher-frequency components (Aery Jones et al., 2021). Critically, both manipulations increase DG and CA3 spiking output, while CA1 firing rates remain largely unchanged, indicating that differences in CA1 spectral content arise from redistributed upstream inhibition rather than frequency-selective transmission or decoding. Moreover, imposing artificial rhythms forces circuits into regimes not naturally expressed by their intrinsic dynamics, producing dissociations between field potentials and cellular firing patterns (Humphries, 2017, 2018; Scarlett et al., 2004). As shown in other systems, transient perturbations can destabilize downstream dynamics without identifying the steady-state computational substrate (Otchy et al., 2015), and structured inhibitory connectivity can strongly reshape network stability and gain without encoding information by frequency (Pfeffer et al., 2013). Frequency shifts observed under perturbation therefore index changes in circuit state and synaptic drive, not frequency as a causal routing variable.

**Fig. 3:**
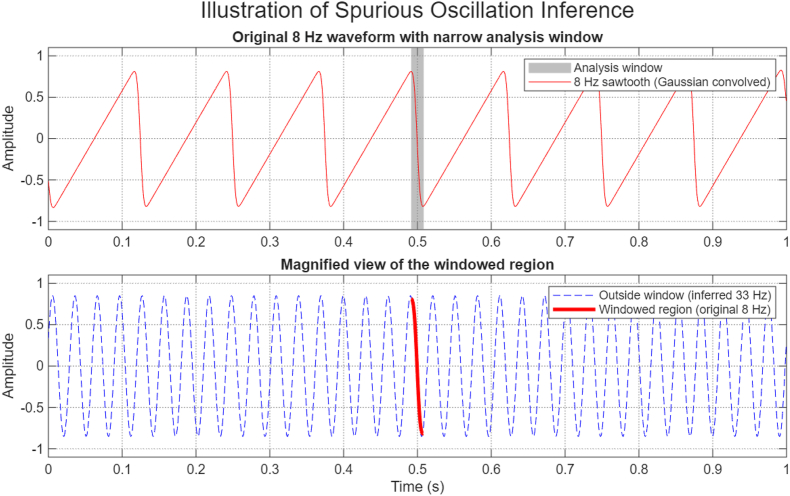
**Example of spurious oscillatory structure induced by short-time windowing of a non-sinusoidal rhythm. *(Top)*** An 8 Hz sawtooth-like waveform (red), generated by Gaussian convolution, shown with a narrow temporal analysis window (gray). ***(Bottom)*** View of the windowed segment (solid red trace) alongside an inferred higher-frequency sinusoidal component (blue dashed line), illustrating how brief temporal windowing of a non-sinusoidal oscillation can falsely suggest the presence of an independent fast rhythm. This effect reflects fundamental time–frequency resolution limits rather than the generation of a distinct oscillatory process.

Neural activity is also highly non-stationary and nonlinear, properties that are only partially captured by conventional spectral methods (Aru et al., 2015). The limitation is not spectral decomposition itself, but how its outputs are interpreted. Power spectra are descriptive. They summarize frequency content over time, but they are insensitive to waveform shape, temporal asymmetry, and mechanistic origin. When gamma activity is broadband, transient, and input-dependent, a localized increase in spectral power does not imply the presence of a discrete oscillator. Instead, it reflects the compound effect of overlapping, emergent circuit dynamics. A critical and often underappreciated constraint arises from the fundamental limits of time–frequency resolution. Wavelet-based approaches explicitly trade temporal precision for frequency precision (Tallon-Baudry et al., 1996). Under these constraints, sharp transitions in a non-sinusoidal waveform are mathematically dissociated from the fundamental frequency and redistributed into higher-frequency components. As a result, theta rhythms with asymmetric or sawtooth-like structure naturally generate spectral energy in the 20–50 Hz range, even in the absence of an independent fast oscillation (Sheremet et al., 2016, 2019). Representing sharp temporal features requires high-frequency sinusoids (Kramer et al., 2008), meaning that brief temporal windowing of such signals can falsely suggest the presence of higher-frequency rhythms (Fig. 3).

These ambiguities can be resolved using bicoherence analysis, which directly tests whether higher-frequency components are phase-coupled to lower-frequency fundamentals. Bicoherence is the third-order cumulant spectrum of the signal, extending the power spectral density (second-order cumulant spectrum) from amplitude to phase. Whereas the power spectrum measures how energy is distributed across frequencies, bicoherence measures whether phase relationships between frequency triplets (f_1_, f_2_, f_1_+f_2_) are stable across time. Because harmonics are defined by phase coupling to the fundamental rather than by their amplitude relative to other spectral components, this question cannot be resolved by amplitude-domain methods (including spectral peak fitting, parametric decomposition, or empirical mode decomposition) regardless of their sophistication (Zhou et al., 2019). For example, Lopes-dos-Santos et al. (2018) applied EEMD followed by ICA to extract spectral components from individual theta cycles, but neither method tests phase coupling, and ICA's assumption of statistical independence is violated by electroneutrality, which couples spectral content across dendritic compartments through closed current loops (Buzsaki et al., 2012; Einevoll et al., 2013). When Zhou et al. (2019) applied bicoherence to the post-EEMD signal, harmonic structure remained intact, demonstrating that the pipeline's extracted components cannot be distinguished from theta waveform features by the methods used to identify them. Multiple studies have shown that putative “slow gamma” components exhibit significant bicoherence with theta, consistent with harmonic coupling rather than independent oscillatory generators (Sheremet et al., 2019; Zhou et al., 2019). This finding accords with cautions raised by Aru et al. (2015) and Jones (2016), who emphasized that apparent cross-frequency coupling can arise from waveform features rather than interactions between distinct neural processes.

Moreover, these findings challenge parametric spectral decomposition methods such as FOOOF (Donoghue et al., 2020), which model the power spectral density (PSD) as the linear superposition of two separable components: an aperiodic 1/f background attributed to asynchronous or stochastic activity, and superimposed periodic peaks interpreted as discrete oscillatory generators. Oscillatory components are extracted by fitting Gaussian peaks onto the PSD, while the background is modeled as a fixed power-law exponent. This decomposition rests on a set of mathematical and diagnostic assumptions that are incompatible with a cascade-based interpretation of neural dynamics. At a foundational level, the method commits a category error. The Fourier transform represents signals using an orthonormal basis of perfectly periodic sinusoids. Every point in Fourier space corresponds to a sinusoidal component with a defined frequency, amplitude, and phase. A power-law spectrum, S(f) ∝ f^−α^, does not describe a distinct class of non-oscillatory activity. It describes how amplitude is distributed across these same periodic basis functions. A 1/f spectrum therefore reflects scale-dependent structure in the signal, not the presence of a separate, aperiodic process. By construction, Fourier space contains no elements that are “aperiodic.” Treating the 1/f component as a separable background is analogous to decomposing an image into square pixels and then attempting to isolate a class of “circular pixels.” The basis does not support such a distinction because the “circularity” is simply a specific spatial arrangement of the square pixels themselves. The same is true for the Fourier decomposition. The mathematical basis does not support such a distinction and the identified “aperiodicity” is an arrangement of perfectly periodic sin waves. As a result, separating “oscillatory peaks” from an “aperiodic background” is not a neutral reparameterization of the data, but an imposed ontology that is not present in the representation itself.

A second, more fundamental methodological limitation of peak-based spectral description is that the power spectral density (PSD) is not an invertible representation of a time series. The PSD is a second-order statistic that retains only amplitude information and discards phase entirely. As a result, infinitely many distinct signals - including phase-locked oscillations, transient bursts, non-sinusoidal waveforms with sharp transitions, deterministic chaos, and filtered stochastic processes - can produce indistinguishable power spectra. In nonlinear neural systems, where phase relationships and temporal asymmetries define the underlying dynamics, this limitation is decisive. Because FOOOF operates entirely in amplitude space, it cannot distinguish genuine oscillatory interactions from spectral structure produced by waveform asymmetry or nonlinear mixing. For instance, by discarding phase, FOOOF cannot inherently identify harmonics, which are, by definition, integer, phase-coupled sine waves. What renders inverse Fourier reconstruction possible is the joint specification of amplitude and phase, not power alone; any method that attempts to infer generative mechanisms solely from the PSD, therefore, operates on an inherently underdetermined representation.

This concern echoes Herbert Jasper's warning about the “Fourier fallacy” (1948). Jasper did not argue against Fourier analysis itself, but against reifying its basis functions as physiological entities. Decomposing a signal into sinusoids is mathematically valid, but assuming those sinusoids correspond to independent neural generators is not. FOOOF reproduces this error in a modernized form. Because the PSD can be fit as a power-law component plus peaks, the method implicitly treats these terms as reflecting separable background activity and discrete oscillatory processes. However, the 1/f structure is the amplitude envelope of the same Fourier basis used to represent the peaks. Deviations from a fitted slope, therefore, do not by themselves establish the presence of independent oscillators.

From the Energy Cascade perspective, the core problem is diagnostic rather than interpretive. By treating spectral slope and peaks as independent causes, peak-based decomposition systematically misattributes changes in slope to changes in oscillatory power. When behavioral demand increases and the spectrum flattens, FOOOF interprets this reorganization as stronger theta or gamma activity superimposed on a stable background. In a cascade framework, the slope change itself reflects enhanced cross-scale energy transfer driven by increased input to the system.

In the hippocampus, theta acts as a forcing scale. Harmonics and broadband gamma activity emerge through nonlinear redistribution of this drive rather than through activation of discrete oscillators. Under these conditions, the spectral exponent is not a nuisance parameter to be removed, but a primary observable indexing the efficiency of energy flow across scales. Methods that subtract or normalize away the 1/f structure therefore discard the very dynamics that explain the emergence of harmonics, the coupling between theta and gamma, and the systematic reorganization of spectral structure with behavioral state. From this viewpoint, peak-centered spectral decomposition is expected to obscure, rather than clarify, the physical processes governing hippocampal population activity. For instance, what FOOOF would parameterize as separable peaks in the 20-50 Hz range tenably reflects a single theta process with harmonic structure (Sheremet et al., 2016).

### 3.7 Laminar anatomy and biophysical constraints of gamma routing

**Fig. 4:**
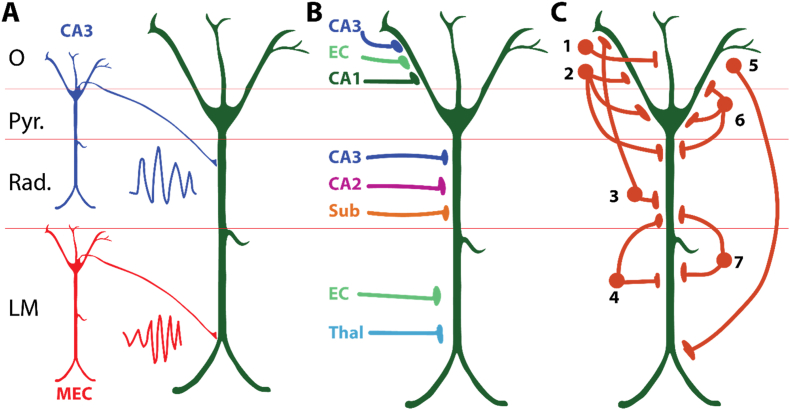
**(A)** Schematic illustrating the common interpretation that excitatory inputs from CA3 and the entorhinal cortex (EC) contribute to “slow gamma” in stratum radiatum (R) and “fast gamma” in lacunosum moleculare (LM), respectively (Colgin et al., 2009; Fernández-Ruiz et al., 2017; compare to Fig. 1B of Fernandez-Ruiz et al., 2023). **(B)** Diagram of excitatory projections across CA1 layers, all contributing to the synaptic transmembrane current. **(C)** Representative interneuron types across CA1. While not shown, rhythmic GABAergic and cholinergic inputs from the medial septum play a critical role in pacing hippocampal theta and modulating interneuron activity across layers (Freund and Antal, 1988). Key for interneurons: 1) axo-axonic, 2) trilaminar, 3) Schaffer collateral associated, 4) perforant pathway associated, 5) oriens-lacunsoum.moleculare, 6) basket cell, 7) neurogliaform. Panel content synthesized from published anatomical and physiological findings (Klausberger and Somogyi, 2008; Lawrence, 2010; van Strien et al., 2009).

Fernandez-Ruiz et al. (2023) illustrate the difficulty of reconciling circuit-based and frequency-based explanations for hippocampal gamma. A key assumption in their framework is that slow and fast gamma are relayed from specific anatomical inputs, with CA3 and medial entorhinal cortex (MEC) treated as distinct frequency sources (Fig. 4A). This mapping is widely implied in the literature (Colgin et al., 2009; Fernández-Ruiz et al., 2017; Schomburg et al., 2014; Tort et al., 2009; Zheng et al., 2016), yet direct evidence that upstream populations intrinsically oscillate at the attributed frequencies is rarely provided. The absence of such evidence is not disproof, but it leaves open a simpler possibility: gamma structure in CA1 reflects local circuit interactions rather than transmission of a carrier frequency from a designated source. The resulting gap motivates frameworks in which gamma is treated as a state-dependent product of synaptic drive interacting with local excitatory–inhibitory circuitry, not as a discrete entity tied to a single anatomical origin.

The anatomy itself already undermines one-to-one mappings between a frequency band and a pathway. CA3 axons densely innervate stratum radiatum but also project into stratum oriens, engaging both pyramidal dendrites and local interneurons (Takács et al., 2008). Entorhinal inputs, although prominent in lacunosum-moleculare, also reach stratum oriens and recruit pyramidal and interneuron populations there (Bell et al., 2021; Deller et al., 1996; Desmond et al., 1994). Stratum oriens additionally receives substantial inputs from medial septum, amygdala, and the contralateral hippocampus (Freund and Antal, 1988; Pikkarainen et al., 1999). These convergences indicate that the extracellular field structure within a lamina reflects the superposition of multiple driving and return currents, rather than a single isolated “channel” (Fig. 4B).

Spectral routing interpretations nevertheless propose that gamma sub-bands separate CA3-derived and entorhinal-derived information streams (Colgin et al., 2009; Schomburg et al., 2014; Fernández-Ruiz et al., 2021). That claim makes a concrete, testable prediction: if “slow gamma” in radiatum and “fast gamma” in lacunosum-moleculare are functional carriers for pathway-specific routing, then gamma-band activity should maintain frequency-matched, cross-layer phase relationships as signals propagate toward stratum pyramidale. Laminar coherence analyses test this directly. Across datasets, theta exhibits high coherence and low phase dispersion across hippocampal layers, whereas gamma coherence is spatially confined and becomes phase-dispersed across laminar boundaries (Berényi et al., 2014; Zhou et al., 2019, 2022).

**Fig. 5:**
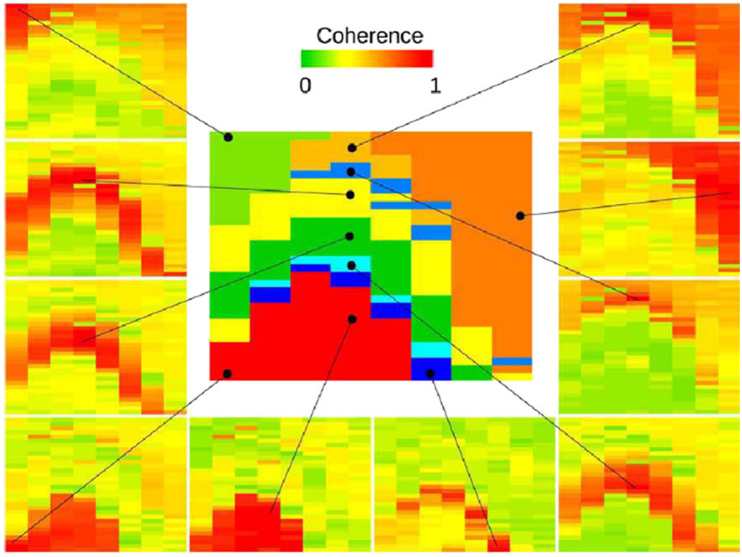
Laminar gamma coherence contradicts frequency-specific routing models. Reference sites (black dots) span hippocampal layers in CA1 (stratum oriens, lacunosum-moleculare, radiatum) through the dentate gyrus. Surrounding panels show coherence (frequency × position) computed between each reference site and all other recording locations on a 256-site silicon probe. Gamma-band (30–90 Hz) coherence clusters align with anatomical layer boundaries, with high coherence within laminae and sharp reductions across boundaries, a pattern that emerged without prior anatomical knowledge and was confirmed by histological reconstruction (Berényi et al., 2014). This laminar confinement is inconsistent with models positing frequency-specific routing across layers. Reproduced from Berényi et al. (2014), *Journal of Neurophysiology*, with permission.

Berényi et al. (2014) computed gamma-band (30–90 Hz) coherence across hippocampal layers with a 256-site probe while rats traversed a track or explored an open arena, observing high coherence within individual dendritic laminae but sharp drops across boundaries (Fig. 5). Zhou et al. similarly report that cross-layer and cross-regional gamma coherence remains low and highly phase-dispersed even when theta coherence is strong. Phase-offset analyses reinforce the point: gamma phase relationships show circular standard deviations exceeding ∼100°, inconsistent with entrainment and incompatible with millisecond-scale routing, whereas theta phase remains tightly clustered across layers and regions (Zhou et al., 2019, 2022). Most critically, recent laminar analyses show that gamma coherence between lacunosum-moleculare (entorhinal input zone) and stratum pyramidale (somatic output zone) approaches incoherent levels under control conditions, contradicting the prediction that “fast gamma” carries entorhinal information to CA1 output (Zhao et al., 2026).

Gamma coherence across hippocampal layers and regions is consistently low. Cross-regional magnitude squared coherence between CA3 and CA1 str. pyramidale peaks near 0.2–0.25 in the slow gamma band (Montgomery et al., 2008; Schomburg et al., 2014), corresponding to roughly 20–25% shared variance. This is modest by any standard and substantially lower than theta coherence across the same distances, which routinely exceeds 0.7 (Zhou et al., 2022). The picture is starker for the entorhinal–hippocampal pathway most frequently invoked in gamma communication models: EC3–CA1 str. pyramidale coherence fell below 0.1 above ∼60 Hz in every animal tested (Schomburg et al., 2014), placing shared variance under 10% at the very frequencies where layer 3 entorhinal input is supposedly routed to CA1. Rather than direct gamma-band communication, a more parsimonious explanation of these low coherence values is that a slower larger amplitude rhythm drives local gamma in the entorhinal cortex and CA1. This then results in small, correlated increases in gamma that are not directly coordinated.

Using a toy model, Zhou et al. (2022) demonstrated that independent local gamma generators, each modulated by a shared theta rhythm with consistent temporal offset, produce cross-regional gamma coherence in the 0.1–0.25 range (matching the empirical values without invoking any interregional gamma synchronization). The observed coherence is therefore explained as a confound of theta-organized drive rather than evidence for a dedicated gamma communication channel. Buzsáki and Schomburg (2015) cautioned that coherence values below 0.1 warrant particular scrutiny, and noted that the critical downstream test of gamma communication, whether CA1 pyramidal cells are entrained to upstream gamma, reveals coupling that is rare and negligible above ∼50 Hz. Fewer than 3% of CA1 pyramidal cells were significantly phase-locked to EC3 gamma above 80 Hz, effectively at chance (Schomburg et al., 2014), a limitation attributed to dendritic low-pass filtering of fast synaptic patterns arriving at distal dendrites (Vaidya and Johnston, 2013). Moreover, interregional gamma coupling is largely attributable to interneuron phase-locking rather than to principal cell synchronization (Schomburg et al., 2014). Schomburg et al. (2014) further demonstrated that even after ICA decomposition, coherence between components reflecting different synaptic pathways remained low regardless of recording site separation, whereas coherence between like components from different shanks decreased monotonically with distance. This pattern is consistent with spatially localized circuit activity that dissipates with distance rather than with pathway-specific signals maintaining coherent identity across space. Notably, the only regime in which cross-regional coherence was high (above 100–150 Hz) was attributable to muscle artifact contamination rather than neural coordination (Schomburg et al., 2014, Fig. S4). These findings pose a fundamental challenge to spectral parcellation models, which do not claim that gamma correlates with pathway activity but propose that gamma oscillations actively route information between regions (Fernández-Ruiz et al., 2021; Colgin et al., 2009). Fernández-Ruiz et al. (2021) describe gamma as enabling a “*target reader area to disambiguate convergent inputs*.” Yet the strongest channel in this proposed mechanism accounts for roughly one-quarter of the shared variance between sender and receiver, a value reproducible by theta-confounded alignment alone (Zhou et al., 2022), while failing to entrain the output neurons it supposedly coordinates. This does not meet the evidentiary standard the framework's own claims require. Together, these coherence patterns are more consistent with gamma reflecting locally generated excitatory–inhibitory dynamics within dendritic domains receiving common synaptic drive than with cross-layer propagation of a frequency-specific carrier. In line with this interpretation, optogenetic manipulations demonstrate that gamma-band activity can be generated locally via excitatory–inhibitory interactions without requiring frequency-specific routing across layers (Sohal et al., 2009).

As a counter, one might propose that low coherence reflects measurement limitations. That is, as gamma's spatially localized, lower-amplitude nature yields poorer signal-to-noise ratios than theta, depressing coherence estimates. But this objection defeats the communication hypothesis rather than rescuing it: if gamma is too spatially confined and noisy to produce reliable cross-regional coherence in the measurement, it is too spatially confined and noisy to reliably coordinate spike timing across those same regions in the brain. Similarly, the concession that “neuronal firing is determined by multiple factors” and that gamma would not be expected to account for all of them is precisely the argument against assigning gamma a privileged mechanistic role. If gamma is one influence among many, it is not the mechanism that “*determines the precise timing of action potential discharge*” (Fernandez-Ruiz et al., 2023). It is a correlate, and the language in the primary literature should be calibrated accordingly. Specifically, 20% shared variance is a real observation, and no one disputes this. However, this value may be an overestimate, as the toy model of Zhou et al. (2022) demonstrates that comparable coherence values can be reproduced with independent, non-communicating gamma generators aligned by theta alone. One would not endorse a multiplexer in which 80% of the signal is noise or unrelated activity, nor a router that loses 80% of its payload. The language used to describe gamma coherence across regions requires recalibration. The issue is not simply that 20% of explained variance is close to the floor of incoherence. It is that the rhetoric of “routing,” “disambiguating,” “controlling,” and “imposing” implies a high-fidelity channel, while the data describe a weak statistical dependency indistinguishable from a confound.

The stratum oriens represents a point of convergence that further complicates frequency-based routing interpretations. Medial septal projections provide cholinergic and GABAergic modulation essential for theta pacing and interneuron recruitment (Freund and Antal, 1988), while amygdala inputs influence network dynamics across both ventral and dorsal CA1 (Pikkarainen et al., 1999). This convergence creates a physiological environment in which oscillatory activity reflects the integration of multiple drives rather than the isolation of separable information streams. If gamma oscillations functioned as frequency-specific routing channels, coherence between stratum radiatum, stratum lacunosum-moleculare, and stratum oriens would be expected to support such segregation. Instead, the low coherence between these layers, combined with heterogeneous input convergence, argues against functional separation by frequency and favors a view in which gamma structure reflects local dissipation under shared constraints. The difficulties faced by spectral routing theories extend beyond laminar anatomy. The hippocampus is not homogeneous: connectivity and input strength vary along both longitudinal (dorsal–ventral) and transverse axes. Medial entorhinal cortex preferentially targets proximal CA1, whereas lateral entorhinal cortex targets distal CA1 (Witter et al., 2000). Oscillatory properties also vary systematically along this axis, including pronounced differences in theta power (Patel et al., 2012). These gradients further complicate attempts to assign invariant frequency functions to specific layers or pathways.

Interneuron diversity and dendritic integration impose additional constraints (Fig. 4C). Basket cells in stratum pyramidale generate gamma-range inhibitory volleys that shape somatic firing (Freund and Buzsaki, 1996; Lasztoczi and Klausberger, 2014), while axo-axonic cells regulate pyramidal output via axon initial segments (Somogyi et al., 1983). Computational models suggest that Oriens-lacunosum moleculare (O-LM) cells, which project to distal dendrites in stratum lacunosum-moleculare, can play a critical role in coordinating theta–gamma interactions at the circuit level (Tort et al., 2007). Importantly, interneuron classes fire at distinct phases of the theta cycle (Klausberger et al., 2003), generating temporally structured inhibition undermining the need for frequency routing. Pyramidal dendrites further complicate matters: their extended arbors integrate synaptic input in a layer-dependent manner (Spruston, 2008), while voltage-gated conductances support nonlinear integration and dendritic spikes that amplify distal inputs (Golding et al., 2002; Magee, 2000). Frequencies propagating through such structures are filtered and transformed via membrane dynamics, undermining assumptions that oscillatory bands remain intact carriers across dendritic space. Despite these constraints, frequency-based routing and multiplexing models are often invoked as solutions to the binding problem. Doing so commits the theory to two specific mechanistic requirements, rarely made explicit: 1) The Selection Problem (The “Gate”), and 2) The Readout Problem (The “Decoder”).

For frequency-based routing to be tenable, a biophysical mechanism must exist that selectively enables one frequency channel while suppressing another. However, no ionic or synaptic mechanisms have been identified that would restrict CA3-driven activity to the 30–50 Hz range. CA3 pyramidal neurons are modulated by theta and active on each theta cycle; if slow gamma is intrinsic to CA3 output, why is it not ubiquitously expressed in CA1 neurons whenever CA3 is active? Relatedly, when both CA3 and entorhinal inputs are concurrently active, what mechanism determines which input CA1 preferentially responds to at a given moment? This gap is particularly evident in studies reporting behavioral modulation of gamma sub-bands. For example, Kemere et al. (2013) reported that 30–50 Hz power decreased with increasing running speed, while higher-frequency gamma dominated during rapid locomotion. Kemere and colleagues invoked an upstream control signal, septal cholinergic modulation, to suppress CA3 input during high-drive states. However, this explanation displaces the control problem. If septal input determines routing, one must specify the mechanism that instructs the septum to switch modes. Without an explicit biophysical link between behavioral variables and frequency-specific suppression, the model relies on a deferred control structure, a “septal homunculus” or switchboard operator, rather than a self-organizing mechanism.

**Fig. 6:**
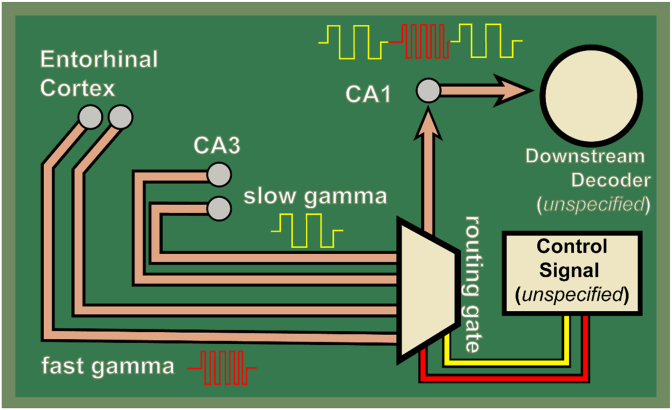
**What frequency-specific “gamma routing” implies as an explicit functional architecture.** Schematic depiction of a common spectral parcellation interpretation in which convergent hippocampal inputs are segregated into discrete, frequency-defined communication channels (e.g., CA3→CA1 “slow gamma” and entorhinal→CA1 “fast gamma”), and CA1 is modeled as a frequency-selective routing element that passes one channel preferentially depending on behavioral demands. Making this logic explicit highlights two mechanistic requirements that are often left implicit. First, a selection mechanism must exist that modulates routing (e.g., toggles gain or selectivity of the routing element) on task-relevant timescales while remaining separable from the signals being routed. Second, a downstream readout must exist that can infer the pathway of origin from population activity based solely on the frequency structure, despite the absence of intrinsic “frequency labels” at the level of spikes and synapses. The schematic therefore serves as a concrete statement of what the routing interpretation commits to: (i) sufficiently stable and separable gamma sub-bands tied to specific pathways, (ii) a biophysically plausible selector that implements channel preference, and (iii) readout mechanisms that can exploit those channel differences given observed coherence and state dependence. In the accompanying text, we evaluate these requirements against hippocampal physiology and show how a cascade/spectral dependence interpretation provides an alternative explanation without invoking independent frequency channels.

Spectral routing theories further imply that downstream targets can demultiplex frequency-coded signals, necessitating a mechanism for the readout of the “transmitted information”, that is, a “Decoder” of some sort. If CA1 segregates information into gamma sub-bands, efferent regions must possess a decoding mechanism capable of distinguishing spikes arriving on different rhythms. Yet spikes carry no intrinsic frequency labels at synapses. Without a verified decoding mechanism, the model encounters an infinite regress analogous to the Cartesian Theater: CA1 is implicitly treated as a final observer that sorts information, rather than as one node in a continuous dynamical system. If downstream neurons integrate spikes over behaviorally relevant windows, for example, tens to hundreds of milliseconds, largely insensitive to gamma phase (Histed and Maunsell, 2014; Mizuseki et al., 2009), any functional separation achieved by frequency routing collapses. In this case, gamma structure reflects local circuit state rather than a preserved communication code. By making these hidden assumptions explicit, Fig. 6 highlights the mechanistic vacuum at the core of spectral parcellation. The theory describes *what* is supposed to happen, “routing,” but leaves unspecified *how* routing is selected and *how* routed signals are decoded. Without these mechanisms, frequency-based routing remains a descriptive metaphor rather than an explanatory account.

A fundamental physical constraint on extracellular field potentials is the principle of electroneutrality (Buzsaki et al., 2012; LORENTE de NO, 1947). Any transmembrane current entering neuronal tissue must be balanced by a corresponding return current. As a result, extracellular field potentials necessarily reflect closed current loops distributed across space, rather than isolated generators confined to a single lamina. This constraint alone challenges interpretations that treat laminar localization of field activity as evidence for functionally segregated oscillatory channels. Current source density (CSD) analysis provides a spatial estimate of net transmembrane current flow by identifying sinks and sources associated with synaptic and return currents. Critically, CSD is a spatial derivative of the extracellular potential and is therefore agnostic to spectral independence. It cannot, by itself, establish the existence of a distinct oscillatory generator at a particular frequency band. Using laminar CSD profiles to validate the presence of a specific rhythm (e.g., “slow gamma”) conflates spatial localization of current flow with spectral independence of an underlying generator.

**Fig. 7:**
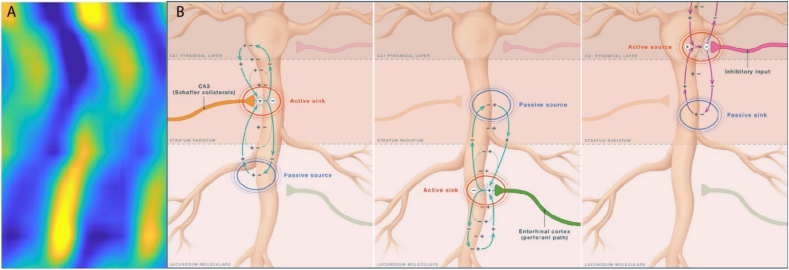
Current source density (CSD) illustrates the spatial distribution of transmembrane current flow during synaptic activation but does not identify oscillatory frequency generators. (A) Representative CSD heatmap across hippocampal layers, with warm colors indicating current sinks (net inward transmembrane current) and cool colors indicating sources (net outward current). (B) Schematic illustrations showing how excitatory and inhibitory synaptic inputs generate closed current loops spanning multiple layers. Active synaptic currents (red) are accompanied by passive or active return currents (blue), forming dipoles that extend beyond the site of synaptic input. CSD analysis localizes current flow but cannot distinguish active synaptic currents from passive return currents, nor does it measure oscillatory power or identify frequency-specific generators. Because current conservation requires that inward and outward currents balance, oscillatory activity, regardless of frequency, will be observable across neighboring layers with complementary polarity. As a result, laminar CSD profiles constrain where current flows, not where a particular oscillation is generated. Panel A CSD unpublished results.

Because CSD reflects the spatial distribution of current flow, it does not distinguish whether those currents arise from a unique oscillatory process or from waveform features of a dominant rhythm (Fig. 7). When signals are band-pass filtered in the presence of a non-sinusoidal carrier such as theta, high-frequency components can emerge from waveform asymmetries, sharp transitions, and burst timing rather than from an independent physiological oscillator. In such cases, filtered activity in the 30–50 Hz range primarily reflects harmonic structure rather than a separable gamma rhythm. Similar issues arise outside of theta states: during sharp-wave ripples, low-frequency gamma power often reflects the temporal envelope or fusion of ripple events rather than a distinct oscillatory carrier (Oliva et al., 2018). Time-locking analyses further exacerbate this problem. Triggering CSD or averaging procedures on peaks detected in a filtered gamma band (often from a reference electrode in stratum radiatum) enforces constructive interference at the trigger site while inducing relative phase cancellation at neighboring electrodes, given the imperfect coherence of oscillations across laminae. This procedure can generate steep local voltage gradients and apparent laminar sinks without implying a distinct synaptic generator localized to that layer.

Accordingly, laminar CSD patterns derived from band-limited signals constrain where current enters and exits the circuit at a given frequency, but they do not uniquely identify where that frequency is generated, nor do they distinguish between active synaptic currents and passive return currents (Einevoll et al., 2007, 2013; Pettersen et al., 2008; Rimehaug et al., 2023). As emphasized by Mann et al. (2005), alternating somatodendritic dipoles can arise from dendritic excitation, perisomatic inhibition, or combinations of both, rendering the underlying generator ambiguous. While previous studies have used CSD to identify layer-specific sinks (Belluscio et al., 2012), the presence of a sink does not establish a unique oscillatory channel.

Two additional observations reinforce this conclusion. First, although CSD analyses may identify maximal sinks in specific layers, coherence analyses show that gamma-band signals do not form a unified cross-laminar communication channel. Gamma coherence drops sharply at laminar boundaries (Berényi et al., 2014), consistent with local dissipation rather than routed signaling. Second, the apparent laminar location of a “gamma generator” is highly sensitive to referencing choices. Csicsvari et al. (2003) demonstrated that changing the reference electrode can reverse the apparent location of dominant gamma sinks, indicating that CSD profiles often reflect alignment choices rather than a unique anatomical origin. Together, these considerations place strong limits on what can be inferred from laminar CSD analyses. CSD is invaluable for identifying where current flows, but it is limited in providing evidence of frequency-specific generators or pathway-selective routing. When combined with low cross-laminar gamma coherence and the known effects of waveform asymmetry, laminar CSD evidence favors an interpretation in which gamma-band structure reflects local dissipation of synaptic drive constrained by circuit geometry, rather than discrete oscillatory channels relaying information across layers.

**Fig. 8:**
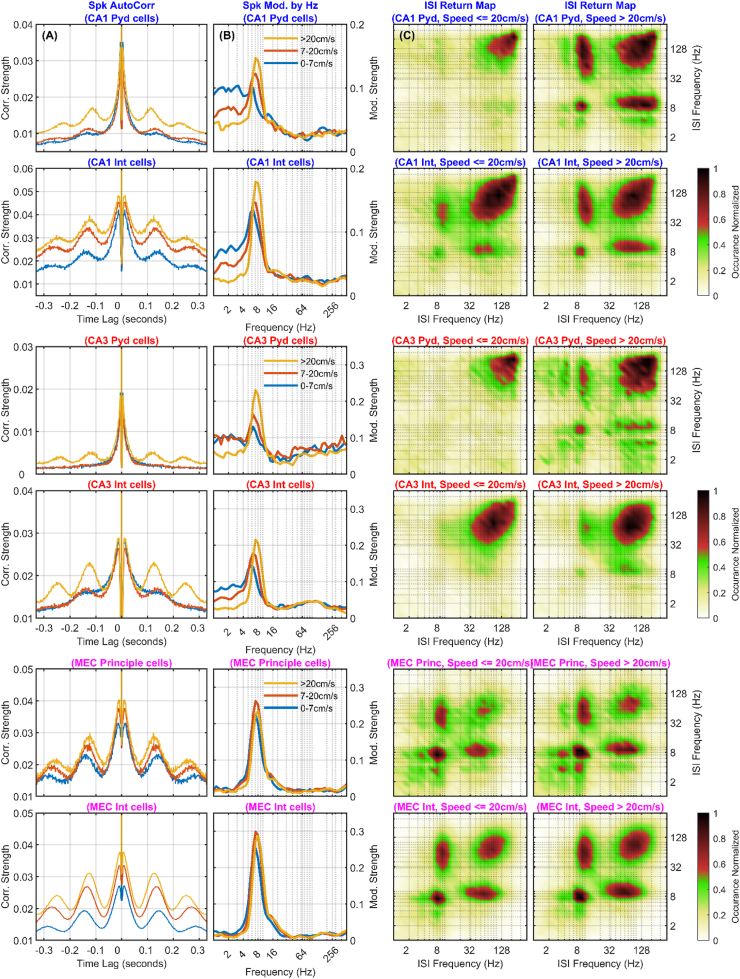
**Testing for intrinsic slow-gamma timescales in spike timing dynamics.** Analysis of single-unit activity from CA1, CA3, and MEC demonstrates that “slow gamma” timescales do not reflect an intrinsic neuronal rhythm. **(A)** Spike Autocorrelograms for Pyramidal (Pyd) and Interneurons (Int) show strong theta modulation (∼125 ms lag) and burst firing (0 ms lag), but lack consistent rhythmic structure in the slow gamma (25–50 ms) range. **(B)** Spike modulation strength by frequency summarizes how strongly neuronal firing is modulated by ongoing LFP rhythms across the frequency spectrum, shown separately for speed bins. Across CA1, CA3, and MEC, modulation strength increases robustly with running speed in the theta range and extends smoothly into higher frequencies as velocity increases. Notably, no distinct peak, plateau, or separable modulation band is observed in the putative slow-gamma range (25–50 Hz). If slow gamma constituted an independent rhythm governing spike timing, a distinct modulation peak or plateau would be expected in the 25–50 Hz range. Instead, modulation strength varies continuously with frequency and behavioral state, with no separable band emerging at any speed. **(C)** Inter-Spike Interval (ISI) Return Maps (plotted as instantaneous frequency). Activity clusters into two distinct regimes: intra-burst intervals (>100 Hz) and inter-burst intervals (Theta, ∼8 Hz). The 30–50 Hz range appears as a transition zone rather than a distinct oscillatory attractor. Had slow gamma reflected an independent oscillatory generator, a consistent attractor or clustering in this range would be expected across regions and behavioral states. No such structure is observed. Note well that this analysis serendipitously affirmed the prior observation of “theta skipping” in the entorhinal cortex (Jeffery et al., 1995; Desmond et al., 1994; Braitenberg and Schüz, 2013) demonstrating its utility in identifying spike modulation frequency**.***(N = [130 CA1 inter, 427 CA1 pyd; 102 CA3 inter, 369 CA3 pyd;* 40 EC *inter,* 46 EC *pyd;] cells; maps are peak-normalized per cell)*.

If slow gamma constituted a fundamental communication channel or independent oscillatory process, the intrinsic firing properties of hippocampal neurons would be expected to exhibit a corresponding temporal signature. Specifically, neurons participating in slow-gamma coordination should show preferred inter-spike intervals, autocorrelogram structure, or phase locking in the 20–50 ms range. This prediction follows directly from spectral routing models: a frequency used to organize communication must be reflected in spike timing statistics of the neurons it coordinates. To test this prediction, we analyzed spike timing dynamics from CA1, CA3, and medial entorhinal cortex (MEC) neurons using inter-spike interval (ISI) return maps, spike autocorrelograms, and point-process spectral density analyses (Fig. 8). Across regions, cell types, and behavioral states (slow versus fast running speed), firing dynamics exhibited a consistent bimodal structure. One regime corresponded to low-frequency theta-paced firing, with characteristic inter-spike intervals on the order of ∼100–150 ms. The second regime reflected high-frequency intra-burst firing, with inter-spike intervals below 10 ms. Critically, no clustering, attractor, or preferred timescale emerged in the 20–50 ms range corresponding to putative slow gamma (20-50 Hz).

[Methods for this figure. **Spike Modulation Strength by Hz.** spike trains were first segmented into 1-s segments, each categorized by locomotor speed into low (0–7 cm/s), moderate (7–20 cm/s), or high (>20 cm/s) speed bins. For each segment, the corresponding local field potential (LFP) was bandpass filtered at logarithmically spaced frequencies using a Fourier filter, extracting oscillatory components across the frequency spectrum. The instantaneous phase of the filtered LFP at each frequency was then computed using the Hilbert transform, and each spike was assigned a phase value based on its timing relative to the ongoing oscillation. The spike-phase relationship was then quantified by binning the phases into 24 equal bins and calculating the spike rate within each bin. Modulation strength for each frequency was determined using the circular concentration of the spike-phase distribution, providing a measure of the extent to which spikes were preferentially locked to particular oscillatory phases. The process was repeated for all cells, separately for each speed bin, and averaged across neurons within each region and cell type. **Spike Timing and ISI Analysis** Single-unit data were drawn from the HC6 dataset (Animals: Fra, Bon, Cor, Fiv). Inter-Spike Interval (ISI) return maps were computed to examine the intrinsic rhythmicity of firing. For each cell, ISIs were converted to instantaneous frequency (1000/ISI). Two-dimensional histograms of successive ISIs (ISI_n vs ISI_(n+1)) were computed for each speed bin. To ensure population averages were not dominated by high-firing-rate neurons, individual maps were peak-normalized (n/max(n)) and then averaged across the population using a capped weighting scheme: cells with firing rates <1 Hz were weighted by their rate, while cells with rates greater than 1 Hz were assigned a weight of 1.]

Spike autocorrelograms reinforced this conclusion (Fig. 8A). Both pyramidal cells and interneurons showed strong theta modulation and prominent zero-lag peaks reflecting burst firing but lacked periodic structure in the slow-gamma range. Point-process spectral density analyses similarly failed to reveal peaks in the 25–50 Hz band, regardless of running speed or behavioral state. Spike–LFP coupling analyses further support the interpretation of no slow gamma modulation. When spike–LFP coherence was computed across frequencies (Fig. 8B), coherence above the first theta harmonic (>18 Hz) fell below 0.05, a level indistinguishable from noise (Zhou et al., 2019). Neurons were strongly entrained to theta and its harmonics but statistically uncoupled from the 25–50 Hz band. This pattern is incompatible with models in which slow gamma provides a timing reference for spike coordination. Inter-spike interval return maps provide a complementary view (Fig. 8C). When ISIs were converted to instantaneous frequency and plotted as successive pairs, activity clustered into two distinct regimes: a theta-paced regime (∼8 Hz) and a high-frequency intra-burst regime (>100 Hz). The 30–50 Hz range appeared only as a transitional zone between these regimes, not as a stable oscillatory attractor. Importantly, this structure persisted across movement speed bins, brain regions, and cell types, indicating that the absence of slow-gamma rhythmicity is not state- or brain region-dependent.

Additional challenges to the feasibility of gamma routing arise from the biophysics of neurons and synapses. Neuronal membranes act as low-pass filters, attenuating high-frequency signals as they propagate through dendrites and across synapses (Ray and Maunsell, 2010; Vaidya and Johnston, 2013). Synaptic transmission is stochastic, introducing timing variability that accumulates across multi-synaptic pathways. These properties limit the fidelity with which high-frequency oscillations can serve as reliable carriers of information across distributed circuits (Akam and Kullmann, 2014). Consistent with these constraints, recent laminar and cell-type–resolved recordings show that inter-regional gamma coherence does not reliably translate into gamma-locked spiking in downstream pyramidal neurons. Instead, gamma synchronization is largely confined to fast-spiking interneurons in feedforward input layers (Spyropoulos et al., 2024). Because pyramidal neurons are the primary carriers of long-range excitatory synaptic activity, this dissociation poses a serious challenge for gamma-based routing models. In this context, coherence may reflect baseline circuit structure or excitability rather than active information transfer (Benchenane et al., 2010; Palanca and DeAngelis, 2005).

Coherence metrics themselves introduce additional interpretive challenges. Statistically significant coherence values often reflect only a small fraction of shared variance (Buzsáki and Schomburg, 2015). Volume conduction can inflate coherence estimates independently of true interaction strength (Miceli et al., 2017), and common input from a third source can generate coherence without direct communication (Bastos and Schoffelen, 2015). Signal-to-noise ratios further bias coherence estimates, particularly in gamma frequencies where power is low and non-stationary (Muthukumaraswamy and Singh, 2011). The low absolute magnitude of inter-regional gamma coherence, typically on the order of 0.2, further challenges its viability as a communication channel, especially when contrasted with theta coherence, which routinely exceeds 0.8 in the same circuits (Zhou et al., 2022).

Taken together, anatomical convergence, laminar coherence structure, spike timing dynamics, as well as biophysical and current-flow constraints converge on a single conclusion: hippocampal gamma activity does not satisfy the necessary conditions for gamma frequency-specific routing or spectral parcellation (**see** Table 2 **for summary**). This interpretation extends beyond the position of Buzsáki and Schomburg (2015) and Schneider et al. (2021), who argue that upstream gamma-paced inputs can be passively reflected in downstream LFPs. We do not dispute this in principle but note that upstream hippocampal populations do not fire rhythmically at the attributed gamma frequencies (Fig. 8), indicating that gamma-range structure in the CA1 LFP reflects local circuit dynamics rather than passively transmitted upstream rhythmicity. These failures are not peripheral. They strike at the core assumptions required for spectral parcellation to function as a coordination mechanism. The hippocampal data do not fail to support frequency-specific routing. Instead, they actively contradict its central predictions. The previously reported slow-gamma modulation is therefore parsimoniously explained by the irregular temporal spacing of theta-modulated bursts and by non-sinusoidal features of the theta waveform that project spectral power into higher frequencies when analyzed with band-limited or wavelet-based methods (Zhou et al., 2019; Sheremet et al., 2019). Within the Energy Cascade framework, gamma-band structure reflects the spectral footprint of local dissipation. Bursts generated by excitatory-inhibitory microcircuits dissipate synaptic drive on fast timescales, while slower global rhythms such as theta constrain the timing and probability of these bursts. Gamma therefore emerges as a consequence of nested circuit dynamics rather than as a dedicated intermediate oscillator mediating pathway-specific communication.

**Table 2:** Summary of limitations of spectral parcellation approaches.

Category of Critique	Specific Issue	Implication for Spectral Parcellation Models
**Epistemological**	**Reification of analytical constructs:** Outputs of spectral decompositions (e.g., Fourier or wavelet transforms) are often treated as evidence for distinct biological processes.	Spectral features such as peaks or “bumps” do not uniquely imply the existence of independent oscillatory generators; they may reflect different expressions of a single, multiscale dynamical process.
**Methodological**	**Waveform shape effects:** Non-sinusoidal slow rhythms (e.g., asymmetric theta waveforms) generate harmonic spectral power at higher frequencies.	Apparent gamma-band components and cross-frequency coupling can arise from waveform geometry rather than interactions between independent oscillators.
	**Referencing dependence:** The spatial localization of sinks and sources can vary with reference choice (Csicsvari et al., 2003).	Laminar CSD patterns may reflect analytical alignment choices rather than uniquely identifying anatomical origins of oscillatory activity.
	**Coherence inflation:** Coherence measures can be influenced by volume conduction or common input from a third region.	Statistically significant coherence does not, by itself, establish direct or selective inter-regional communication.
**Biophysical**	**Laminar confinement of gamma:** Gamma-band coherence is typically high within layers but drops sharply across laminar boundaries (Berényi et al., 2014; Zhao et al., 2026).	Gamma activity appears locally organized and does not reliably support coordinated signal transmission across layers.
	**Frequency-dependent attenuation:** Membrane and synaptic properties impose low-pass filtering on neural signals.	High-frequency activity is increasingly attenuated with distance, limiting its suitability for long-range communication.
	**Pervasiveness of synchrony:** Near-zero-lag phase alignment cannot establish directed communication in systems with finite conduction delays.	Synchrony is more parsimoniously interpreted as reflecting shared input or global state constraints rather than selective information transfer or routing.

The cascade framework does not require theta specifically as the organizing rhythm; gamma couples to whatever slow periodic drive is available, including respiration-entrained oscillations (Lockmann et al., 2016; Tort et al., 2018) and the neocortical slow oscillation (Isomura et al., 2006). The effects of entorhinal cortex lesion on hippocampal oscillatory dynamics reported by Bragin et al. (1995) are directly consistent with the present framework. In that study, bilateral removal of the entorhinal cortex eliminated fast gamma (40–100 Hz) activity in the dentate hilus while simultaneously giving rise to large-amplitude, lower-frequency (25–50 Hz) rhythmic activity in CA3–CA1 stratum radiatum. Theta power was concurrently reduced by 50–70%. The loss of dentate gamma is expected because this rhythm depends on feedforward recruitment of hilar interneurons by entorhinal afferents via the perforant path (Bragin et al., 1995; Buzsáki, 1984; Ewell and Jones, 2010). Perforant path stimulation preferentially drives fast-spiking interneurons over granule cells (Ewell and Jones, 2010; Scharfman, 1991), and the net effect of entorhinal input on granule cell population output is suppressive through di-synaptic feedforward inhibition (Ewell and Jones, 2010; Li et al., 2013; Pofahl et al., 2025). In vivo calcium imaging confirms that medial perforant path activation produces widespread inhibition across granule cells, with only ∼2% of the population recruited per event (Pofahl et al., 2025). Thus, removing the entorhinal cortex eliminates the primary afferent drive sustaining both dentate interneuron-generated gamma and feedforward inhibitory tone onto granule cells.

The emergence of high-power rhythmic activity in the stratum radiatum after entorhinal lesion should not, however, be interpreted as the unmasking of a second physiological gamma oscillator. Stratum radiatum is the termination zone for CA3 Schaffer collateral projections (Bragin et al., 1995), and the appearance of large-amplitude rhythmic currents in this layer indicates that CA3 recurrent circuitry has become pathologically activated. The mechanism is straightforward: loss of entorhinal input removes feedforward inhibitory control over dentate granule cells (Dasheiff and McNamara, 1982; Ewell and Jones, 2010; Tran et al., 2019), disinhibited granule cells deliver unfiltered excitatory drive to CA3 through mossy fiber “detonator” synapses (Henze et al., 2002), and the resulting hyperexcitation of the CA3 recurrent network generates hypersynchronous activity that propagates to CA1 via the Schaffer collaterals. This interpretation is supported by the dentate gate hypothesis, which holds that under normal conditions the dentate gyrus filters entorhinal input and prevents its propagation to CA3 (Heinemann et al., 1992; Lothman et al., 1992), and that gate failure permits epileptiform activity to invade CA3 (Behr et al., 1998; Krook-Magnuson et al., 2015). It is also consistent with the observation that electrolytic entorhinal lesions produce limbic seizure activity (Dasheiff and McNamara, 1982), and that traumatic brain injury, which similarly compromises feedforward activation of dentate parvalbumin-expressing interneurons, causes dentate hyperexcitability (Folweiler et al., 2020). In aged rats, selective loss of feedforward inhibition recruited by lateral entorhinal cortex input, rather than loss of direct excitatory drive onto granule cells, distinguishes cognitively impaired from cognitively intact individuals (Tran et al., 2019), further supporting the functional primacy of the feedforward inhibitory pathway. Bragin et al. (1995) themselves concluded that “*gamma oscillation in the CA3-CA1 circuitry is suppressed by either the hilar region or the entorhinal cortex.*” The present framework refines this interpretation: the entorhinal cortex, through feedforward inhibition, maintains the dentate gate that normally prevents pathological CA3 activation. Its removal produces exactly the oscillatory signature expected from gate failure, loss of organized, physiologically entrained rhythmic structure in the dentate and emergence of hypersynchronous activity in downstream CA3 networks.

This interpretation carries a broader implication for the cascade framework. Under normal conditions, the dentate gate functions as a dissipation bottleneck: feedforward inhibition ensures that only a small fraction of the entorhinal drive propagates to CA3, preventing energy from accumulating in the recurrent network. When this bottleneck is removed, activity is no longer dissipated at the gate but instead recirculates within CA3's dense recurrent collaterals, potentially accumulating and amplifying at a single network scale rather than propagating hierarchically toward dissipation. The resulting hypersynchronous state is the predicted consequence of removing one level of organized dissipation from the cascade. Notably, such pathological states are inherently self-limiting: synaptic vesicle depletion, extracellular potassium accumulation, and metabolic exhaustion impose the same thermodynamic endpoint that the intact cascade achieves through organized circuit dynamics (Lado and Moshé, 2008). The cascade, while offering a description of spectral structure, also describes a control architecture whose organized dissipation at each scale prevents energy from accumulating pathologically at any single scale.

Importantly, rejecting spectral routing does not imply that oscillatory activity is epiphenomenal or irrelevant. Rather, it necessitates a shift in interpretation. Gamma-band structure reflects how synaptic drive is locally redistributed and dissipated by circuit architecture, inhibitory feedback, and membrane time constants. Slower rhythms such as theta constrain the timing, probability, and spatial coordination of this dissipation across scales. Oscillatory structure thus emerges from nested circuit dynamics rather than acting as an independently programmable control signal. This conclusion motivates frameworks that treat oscillations as scale-dependent consequences of energy flow through recurrent networks, rather than as discrete communication channels assigned to specific frequencies. In the next section, we formalize this alternative perspective and articulate the predictions that distinguish spectral dependence from spectral parcellation.

### 3.8 The separability assumption and its consequences

Before evaluating more modest defenses of spectral parcellation, that gamma is layer specific and does not entrain downstream action potentials, it is worth stating what the strongest version of the framework claims. Fernández-Ruiz et al. (2021) proposed that ‘*interregional gamma-time-scale spike coordination is a mechanism of neuronal communication*’ and that ‘*pathway-specific gamma oscillations route task-relevant information between distinct neuronal subpopulations in the entorhinal-hippocampal circuit.*’ In their account, assemblies of neurons firing within gamma time frames in an upstream region *‘most effectively discharge their downstream partners*,’ and sending neuronal messages by ‘*segregated gamma-frequency carriers allows a target “reader” area to disambiguate convergent inputs*.’ This framework commits to several concrete claims: that upstream circuits generate distinct gamma frequencies, that these frequencies entrain specific downstream cell types during specific tasks, and that disrupting gamma spike timing impairs learning. These claims constitute a complete mechanistic account in which gamma oscillations serve as frequency-labeled carriers of pathway-specific information, with dedicated upstream generators, downstream readers, and task-specific routing. A more modest potential defense of spectral parcellation, however, proposes instead that band-limited activity serves as a proxy for pathway engagement. That is, gamma is an indirect readout of which upstream circuit is currently active, without requiring that the oscillation itself mediates communication or psychological function. Under this interpretation, different upstream circuits with different temporal properties impose spectrally distinguishable synaptic currents on a shared downstream target, and these components can be read out from the local field potential even if downstream neurons are not entrained to them.

The mechanistic and proxy interpretations are not interchangeable, but they are routinely treated as such. They posit different causal architectures: the mechanistic version places gamma inside the causal chain from afferent drive to spike output (drive produces gamma, gamma entrains downstream neurons), whereas the proxy version places gamma outside it (drive produces both gamma and spike output as parallel consequences, with gamma serving as a correlated indicator rather than a causal intermediary). These architectures generate different predictions. If gamma routes information, it must entrain downstream output neurons, a testable prediction that fails empirically (Section 3.7). If gamma is simply a proxy, disrupting it should not impair computation, and the framework reduces to a methodological convenience that theta-band laminar analysis already provides, as developed below. The persistent alternation between these positions insulates the framework from falsification: mechanistic language is used to motivate experiments and secure interpretive significance, while proxy language is deployed to deflect biophysical critiques. A framework that occupies both positions as needed, without specifying the conditions that distinguish them, cannot be evaluated as a scientific hypothesis.

The interpretation that gamma is simply a proxy or a readout of afferent input is not maintained in the primary literature. Fernandez-Ruiz et al. (2023) propose that the interplay between gamma frequency inputs ‘*determines the precise timing of action potential discharge of CA1 pyramidal cells*' and that gamma-paced spiking in a source region ‘*potentially entrain(s) target neurons.*’ Lasztoczi and Klausberger (2014) conclude that CA1 dynamics are ‘*structured by rapid, concerted dynamics imposed by converging gamma oscillatory networks.*’ Indeed, the title of Lasztoczi and Klausberger (2014) states that place cells “*couple to three different gamma oscillations*,” and the paper concludes that “*an individual CA1 place cell can combine and relay information from multiple gamma networks,*” language that commits to gamma shaping spike output, not serving as a spectral readout. The coherence data reviewed in Section 3.7, in which fewer than 3% of CA1 pyramidal cells are significantly phase-locked to upstream gamma above 80 Hz (Schomburg et al., 2014), directly challenge this claim.

The parcellation framework generates testable predictions, and these predictions have been evaluated. If gamma constitutes the activity motif through which computational operations are organized (Fernandez-Ruiz et al., 2023), then removing the pathway that generates a given gamma component should abolish the associated operations. Brun et al. (2008) removed CA3 input entirely and CA1 place fields persisted. Zutshi et al. (2022) silenced CA3 completely and place fields and stable assembly expression persisted. Zhao et al. (2026) found that when CA3 was inactivated, slow gamma was not selectively abolished, whereas entorhinal inactivation produced a broadband spectral reduction rather than the selective elimination of fast gamma. The computational operations that slow gamma supposedly ‘constitute’ and ‘determine’ continued without the pathway that supposedly generates them, and the spectral consequences of pathway removal were broadband rather than band-specific.

What potentially follows these falsifications is perhaps not a revision of the framework but a dilution of its claims. For instance, the computations that gamma reportedly supports and the operating modes it indexes potentially become less specified, each reformulation broader and less constrained than the last. 'Gamma routes information’ becomes ‘gamma reports underlying computations,’ which becomes ‘gamma reflects operating modes,’ which becomes ‘gamma indexes computational states' whose content is never defined. This pattern of retreat preserves the framework's vocabulary while emptying it of testable content. A framework that generates predictions, sees them challenged, and responds by redefining what gamma 'reports' has not been refuted in its proponents' eyes because the target keeps moving.

Even if every mechanistic and functional commitment were withdrawn, if gamma were proposed solely as a spectral signature indicating to the experimenter which afferent pathway is currently dominant, the resulting claim would concern methodological convenience rather than biological mechanism. As such, it must be evaluated against the methods already available for the same purpose. Theta phase and laminar anatomy already specify which pathway dominates at a given moment (Brankack et al., 1993; Buzsáki et al., 1986; Mizuseki et al., 2009), and because the local field potential is primarily a consequence of synaptic transmembrane current (Buzsaki et al., 2012), with theta power exceeding gamma by at least an order of magnitude (Sheremet et al., 2019), theta is the parsimonious candidate for examining excitatory drive into CA1 layers. Gamma-based pathway identification is therefore redundant. It is also circular: the claim assumes that band-limited activity in a given lamina originates from the afferent pathway projecting there, which is the conclusion the proxy framework is supposed to establish, not presuppose. Our ISI analysis bears directly on this premise (Fig. 8, developed in full below): CA3 neurons do not exhibit slow-gamma-frequency rhythmicity, undermining the foundational assumption on which even the purely methodological reading depends.

Optogenetic perturbation studies do not resolve this circularity. Fernández-Ruiz et al. (2021) imposed artificial 53 Hz stimulation on entorhinal interneurons, disrupting excitatory cell firing throughout the targeted region, and observed selective reduction of gamma sub-bands in the DG molecular layers receiving projections from the perturbed region. However, this selectivity is predicted by laminar anatomy alone: disrupting an upstream region reduces synaptic drive in its target layer, a result that theta-band CSD would equally reveal. The frequency-specific framing adds no information beyond what the known projection pattern already supplies. Moreover, as noted above (Section 3.6), imposing artificial rhythms forces circuits into regimes not naturally expressed by their intrinsic dynamics, and the resulting disruptions index changes in circuit state, not frequency as a causal variable. Gamma-as-proxy is thus redundant with theta-based laminar analysis and dependent on an unverified generator assumption that available evidence contradicts.

Lopes-dos-Santos et al. (2018) hypothesize that theta-nested spectral components reflect ‘*flexible switching of the hippocampal network between different operating modes, such as memory encoding and retrieval.*’ Their empirical demonstration that spectral content covaries with independently defined task stages (learning versus probe) is methodologically sound. The problem is not the correlation but its reification: throughout the paper, correlational findings (“associated with,” “correlated with”) are restated as ontological claims about the circuit's computational architecture. Spectral content that “reports” or “reflects” operating modes treats the existence of those modes as established and merely asks how we detect them.

The authors' own analysis works against the mode interpretation. They report that spectral component strengths *‘lie on a multidimensional continuum rather than clustering into non-overlapping subsets*,’ a finding more consistent with continuous spectral redistribution under varying drive than with discrete operating modes switchable on a cycle-by-cycle basis. Rather than following this finding to its natural conclusion, the paper absorbs it by softening the framework's terms: modes become ‘*dynamically weighted*’ rather than binary, the circuit ‘*can hold a collection of operating modes ranging from mainly reading (retrieving) to mainly writing (encoding)*.' But a continuum along which neither extreme is ever fully absent is not flexible switching between modes. It is a network that performs both operations continuously, with varying emphasis determined by current drive conditions. This distinction matters: “flexible switching” implies a computational architecture organized around discrete state transitions, whereas continuous variation under changing drive implies spectral redistribution governed by the network's transfer properties. The tension between the continuous data and the categorical interpretation is not confined to the discussion; it runs through the analysis itself. The authors extract continuous projections of each theta cycle onto each spectral component, confirm that these projections form a continuum without cluster boundaries, and then impose an arbitrary threshold to define ‘*strong tSC cycles*' for all subsequent analyses. The spiking correlates, SWR reactivation results, and task-stage modulation that constitute the paper's major findings are all computed from these thresholded categories. Because no natural boundaries exist in the data, the categories are products of the analytical pipeline rather than discoveries about neural computation. ICA applied to a continuously varying spectrum will extract axes of maximum independent variation, as expected, but those axes describe how the spectral distribution reshapes under changing conditions, not evidence that the circuit switches between discrete computational states. The downstream correlates characterize the bins created by the thresholding rather than providing independent evidence for the existence of the categories themselves.

Even the passage most frequently cited in support of the proxy interpretation, that gamma ‘does not per se implement any specific function (e.g., attention or memory recall), but rather reports the underlying computations and communication channels for information processing’ (Fernandez-Ruiz et al., 2023), replaces named cognitive functions with unnamed ones while retaining the same structural claim: that gamma indexes distinct computational operations. The same paper proposes to move ‘over and above’ frequency-based definitions of gamma toward circuit-level identification, treating gamma oscillations as “elementary units of collective neural activity” analogous to action potentials. Yet the paper's own analysis systematically strips gamma of every property this framing requires. Gamma amplitude is graded rather than all-or-none. Frequency varies with drive and behavioral state. Generating circuits overlap with those producing other oscillatory phenomena. Neuronal participation is stochastic across cycles. And gamma oscillations are, in the authors' own assessment, ‘not “real” physical units.’ Having documented that gamma lacks discreteness, stability, exclusivity, and identifiable boundaries, the paper nonetheless concludes that ‘gamma oscillations represent units of operating neural circuits.’ The same structure recurs throughout: the paper states that disrupting entorhinal-dentate gamma synchrony selectively impairs spatial or object learning (Fernández-Ruiz et al., 2021), then insists that gamma does not “implement” function but merely “reports” the circuit interaction that implements it, a distinction no available experiment could adjudicate, since disrupting gamma necessarily disrupts the excitatory-inhibitory dynamics that produce it. At a pivotal moment, the framework's central tension is compressed into the assertion that gamma oscillogenesis ‘*pertains to dynamical, yet well-defined, neural circuits*,’ where ‘dynamical’ absorbs every instability the paper has catalogued and ‘well-defined’ asserts the stable entity the framework requires, with the conjunction ‘yet’ doing the work of reconciliation without argument. Broadly, when a theory exhibits persistent alternation between positions, it is not an incidental ambiguity but a structural feature of the framework, one that complicates falsification by ensuring that no single formulation is held long enough to be tested.

Buzsáki (2020) diagnosed this failure mode in oscillation research, demonstrating that fifty years of attempting to assign behavioral correlates to hippocampal theta produced no convergence and no testable null hypothesis, only an ever-expanding list of competing psychological labels mapped onto the same brain rhythm (his Fig. 1). Buzsaki identified the core error as treating subjectively defined terms as independent variables and searching for their brain correlates, rather than deriving behavioral descriptors from brain mechanisms themselves. The gamma parcellation program recapitulates this logic at a finer spectral scale: ‘slow gamma mediates retrieval’ and ‘fast gamma supports encoding’ are precisely the kind of frequency-function assignments his analysis warns against. Replacing ‘retrieval’ with ‘operating mode’ or ‘underlying computations' does not escape this critique. Rather, it reproduces it with vaguer terminology while preserving the same structural commitment to frequency-function correspondence. To its credit, grounding the mapping in identified anatomical pathways constrains which assignments are plausible, and represents a genuine advance over unmotivated frequency-function correspondence. However, this constraint depends on stable pathway-frequency relationships, and as developed above, frequency scales continuously with drive rather than categorically with pathway identity. Anatomical grounding narrows the space of possible assignments but does not resolve the fundamental problem: the absence of a testable null hypothesis for what would constitute evidence against the proposed frequency-function correspondence.

The proxy and mechanistic interpretations, having both failed on their own terms, a more general dilemma confronts any version of the parcellation framework. Either 1) gamma is phase-locked to theta, in which case it is a dissipative consequence of theta-organized drive (local excitatory-inhibitory circuits responding to synaptic currents whose timing is constrained by theta) and gamma-band activity provides no additional information about pathway engagement that theta-band laminar analysis does not already supply. Under this interpretation, what has been called gamma-mediated coordination was theta-modulated local circuit activity all along. Or 2) gamma is independent of theta, carrying its own oscillatory timing structure not derivable from theta. For instance, consider the claim that distinct gamma bands appear selectively on individual theta cycles (Colgin et al., 2009; Lopes-dos-Santos et al., 2018). This claim requires both a biophysical mechanism capable of generating gamma-frequency timing independently of the theta-organized drive that dominates hippocampal field potentials by at least an order of magnitude (returning to the gate problem, Fig. 6, for which no solution has been identified) and a specification of what information independent gamma timing provides beyond what theta phase and laminar anatomy already supply. Neither requirement has been met. Bicoherence analyses bear directly on this dilemma: putative slow-gamma components in the 20-50 Hz range are phase-locked to theta at integer ratios (Sheremet et al., 2019; Zhou et al., 2019), placing available evidence on the first horn. Gamma is a consequence of theta-organized drive, and its coordination is organized by theta.

More fundamentally, both the proxy and mechanistic interpretations share a hidden assumption. Specifically, that gamma constitutes a separable component of circuit dynamics whose contribution can be isolated and evaluated independently of the processes that generate it. This assumption is a mereological error: the confusion of a part with an independently manipulable entity. Gamma-range spectral power is not a discrete signal added to theta, but rather an obligate consequence of the same synaptic currents, shaped by the same membrane time constants and excitatory-inhibitory interactions, that produce the theta waveform and its nonlinear features. Mizuseki et al. (2009) proposed that theta cycles create temporal windows permitting ‘a considerable degree of computational independence in subdivisions of the EC-hippocampal loop.’ We endorse the independence but note that it is regional, not computational in the sense implied by parcellation models. Within each such window, CA1 pyramidal cells integrate concurrent excitatory drive from multiple afferents, feedback and feedforward inhibition from diverse interneuron classes, dendritic nonlinearities, and neuromodulatory state. The output emerges from the nonlinear interaction of all these factors, not from any single spectral component.

When an upstream pathway is activated within a theta window, synaptic drive arrives at the target layer, local E-I circuits respond with gamma-range activity, and pyramidal cells adjust their firing. These are parallel consequences of the same cause (increased drive), not a causal chain in which drive produces gamma, which then produces spiking. Asking whether gamma ‘contributes to’ spike timing within a theta cycle is analogous to asking whether the whitecap ‘contributes to’ the ocean wave from which it emerges, or ‘produces further information beyond’ what the larger wave provided. The whitecap is not separable from the wave's energy; it is the wave's energy at a particular scale. This is not to say that gamma-range activity is causally inert (local inhibitory circuits operating at gamma timescales certainly shape spike timing), but that gamma is not independently manipulable. Independent causal efficacy requires that a variable can be varied while its generative context is held fixed (Woodward, 2003) and this condition is not met. Interventions that preferentially reshape gamma-range dynamics, such as altering inhibitory time constants, receptor distributions, or recurrent connectivity, do not isolate gamma. They alter the network's spectral transfer function, changing the ratio of recurrent amplification to dissipation across scales. These are changes to the medium, not to a signal carried within it.

Apparent dissociations between gamma and theta power reinforce rather than challenge this conclusion: when network hyperexcitability increases recurrent amplification while afferent drive decreases, the spectral redistribution reflects a shift in the medium's transfer properties, not independent variation of a separable signal. The same logic extends to seizure, where recurrent amplification overwhelms dissipation entirely, producing spectral reorganization that no parcellation framework can describe because the separable components it depends on cease to exist. Attributing a separable causal role to gamma, in any version of the parcellation framework, mistakes a scale-dependent description of circuit dynamics for an independently manipulable causal agent.

The parcellation framework requires that upstream populations generate gamma-frequency output distinguishable from theta-organized activity. This requirement faces a biophysical dilemma. Either CA3 neurons produce theta-modulated bursts whose temporal structure falls in the gamma range, in which case gamma is not an independent signal but a description of what theta-organized firing looks like at a finer timescale, and the communication attributed to gamma is organized by theta. Or CA3 possesses a dedicated mechanism that generates 30-50 Hz rhythmicity independent of theta pacing. The latter requires specifying the mechanism.

GABA_A_-mediated feedback from PV + basket cells is the primary candidate for gamma generation, but gamma frequency in such networks scales continuously with excitatory drive (Traub et al., 1996). The parcellation framework does not require a perfectly fixed frequency, but it does require that different pathways produce reliably distinguishable frequency ranges. Continuous scaling undermines this requirement: as drive varies across the physiological range (e.g., with changes in running speed), a single pathway's output traverses frequencies the framework assigns to different functional categories. For pathway-frequency mapping to hold, each pathway's frequency range would need to remain non-overlapping with every other pathway's range across all physiological drive levels, a condition that continuous frequency-drive scaling makes implausible. Bicoherence analyses confirm this: spectral content scales continuously with theta power (Sheremet et al., 2019), not categorically with pathway identity, and the 20-50 Hz range behaves as a region of the spectrum governed by the overall energy budget rather than as a discrete channel tied to a specific afferent source. No mechanism has been identified that would lock CA3 output to a specific gamma frequency independent of drive magnitude.

Even if such a mechanism existed, the signal faces progressive degradation at every stage of transmission: probabilistic synaptic release reduces temporal precision (Lisman, 1997), dendritic cable properties impose frequency-dependent attenuation that preferentially filters high frequencies (Vaidya and Johnston, 2013), and postsynaptic currents exhibit decreasing ability to generate oscillatory extracellular potentials at higher frequencies (Schomburg et al., 2012). The biophysics of neural tissue works against gamma transmission at every step from generator to target.

The ISI analysis presented here (Fig. 8) resolves this dilemma empirically: CA3 neurons fire in two regimes, theta-paced and intra-burst, with no preferred structure at 20-50 ms intervals. The gamma generator that the parcellation framework requires does not appear in the spiking data of the neurons it is attributed to. The alternative, treating oscillatory structure as an emergent consequence of multiscale energy redistribution under biophysical constraints, is developed in the following section.

By contrast, the energy cascade framework specifies concrete conditions under which it would be falsified (Section 4.4), offering the field a path forward that the parcellation framework, in its current form, cannot provide.

## 4 Spectral dependence models of neural coordination

The limitations of spectral parcellation-based theories motivate a re-evaluation of how neural coordination is conceptualized. This section examines alternative frameworks that move beyond frequency-specific assignments of cognitive function, focusing on two complementary perspectives: (1) the energy cascade model and (2) theta as a coordinating scaffold. These approaches provide mechanistic accounts of neural dynamics without invoking millisecond-scale synchrony as a primary explanatory principle. Here, *synchrony* refers specifically to the precise temporal alignment required by binding-by-synchrony (BBS) and communication-through-coherence (CTC) models; broader notions of co-activation or rate covariance are not under critique. By emphasizing biophysical constraints and firing-rate modulation, these frameworks describe how activity is coordinated across spatial and temporal scales without requiring discrete, frequency-labeled communication channels.

### 4.1 The energy cascade model for neural coordination

Extracellular electrodes detect voltage fluctuations arising from transmembrane synaptic currents. Spectral power reflects the variance of these voltages and is commonly used as a proxy for the magnitude of underlying synaptic activity. Although energy is not measured directly, the term is used here as a compact, biophysically grounded description of how the magnitude and timing of synaptic transmembrane currents are redistributed across temporal scales. Within this framework, phenomena often labeled as “oscillations,” “bursts,” or “cross-frequency coupling” are not functionally separable units that can be selectively generated, maintained, and decoded by dedicated mechanisms (as implicitly required when frequency bands are treated as independent communication channels) but structured patterns reflecting how circuit dynamics redistribute synaptic drive across temporal scales. These currents propagate through a neural substrate characterized by fractal organization across spatial scales. The hippocampal formation exhibits self-similar structural complexity (Gutierrez Aceves et al., 2018), consistent with broader findings that cortical morphology follows universal fractal scaling with dimension df ≈ 2.5 (Wang et al., 2024). A fractal dimension substantially above 2.0 indicates that neural tissue contains structural complexity across spatial scales (e.g., branching dendrites, nested circuit loops, and hierarchically organized connectivity), providing the physical substrate through which activity can redistribute across temporal scales. The specific value is less important for the cascade framework than the confirmation that the substrate is not smooth or homogeneous but contains structure at every measurable scale. Fractal dimensionality increases systematically during development, reflecting progressively more space-filling dendritic and axonal arbors. This geometry has direct consequences for neural dynamics. As Buzsaki (2006) noted, power spectra exhibit scale-invariant 1/f characteristics because signals propagate through fractal networks where dissipation occurs simultaneously across multiple spatial scales. The energy cascade thus reflects a geometric reality: activity flows from large-scale coordination to local circuit dissipation.

The energy cascade framework builds on earlier formulations (Buzsaki and Draguhn, 2004; Buzsáki and Watson, 2012; Freeman, 2000), drawing inspiration from Kolmogorov's theory of turbulence. In neural systems, this cascade reflects *a hierarchical flow and dissipation* of energy, in other words, the redistribution of ionic currents: slow rhythms such as theta inject energy into the network, which is progressively transformed through circuit interactions rather than transmitted as frequency-labeled signals. The result is a directional flow across temporal scales, from global coordination to local dissipation (Sheremet et al., 2018). At intermediate scales, this drive conditionally recruits local excitatory–inhibitory feedback loops, whose kinetics shape higher-frequency components. Gamma-band activity reflects the local circuit response to increased synaptic drive, not a separate communication channel. Interneuron classes such as PV^+^ and SST^+^ cells do not transmit frequency-specific signals but instead determine how excitatory input is locally balanced and dissipated. Differences in membrane time constants, synaptic kinetics, and subcellular targeting shape inhibitory feedback timescales and, consequently, the spectral content of the extracellular signal. At the smallest scales, energy is ultimately dissipated through neuronal spiking and other microscopic processes. From this perspective, gamma oscillations mark circuit activation shaped by excitatory–inhibitory balance rather than specialized cognitive signals (Merker, 2013). Cross-frequency coupling follows naturally: slow rhythms modulate excitability, and faster local dynamics emerge in a phase-dependent manner without invoking an additional coordinating mechanism.

Energy dissipation in neural tissue occurs through multiple scale-dependent processes. Synaptic transmission is inherently stochastic, with release probabilities varying widely across synapse types (Lisman, 1997), acting as a filter on high-frequency energy. Dendritic cable properties impose frequency-dependent attenuation, with higher frequencies experiencing greater loss as signals propagate through fractal arbors (Golding et al., 2005; Vaidya and Johnston, 2013). Metabolic constraints further limit sustained high-frequency activity, while inhibitory feedback and refractory dynamics introduce history-dependent temporal filtering at the circuit level. Sustained high-frequency firing is metabolically costly due to the energetic demands of Na^+^/K^+^-ATPase activity required to restore ionic gradients after each action potential (Attwell and Laughlin, 2001). Inhibitory feedback imposes refractory periods on population activity, and short-term synaptic depression introduces history-dependent gain reduction at excitatory synapses (Zucker and Regehr, 2002), both of which act as temporal filters limiting the propagation of high-frequency structure.

### 4.2 Theta oscillations: large-scale coordination and computational independence in neural networks

Theta oscillations (4–12 Hz) reflect large-scale rhythmic activity that supports coordination across distributed neural populations. Rather than assigning specific cognitive functions to theta, it is more accurate to view it as a biophysical scaffold for transmembrane currents that organize network dynamics across space and time (Buzsaki, 2002). Theta does not encode task variables; instead, it reflects the global state of network engagement. The large-scale coordinating influence of theta follows directly from physical constraints imposed by neural tissue. Membrane time constants and axonal conduction delays favor slower rhythms when coordinating activity across distant brain regions (Kopell et al., 2000). Axonal conduction velocities in entorhinal-hippocampal pathways span 0.6–3 m/s (Andersen et al., 1969; Empson and Heinemann, 1995), and the temporal delays between population activity in successive stages of the EC-hippocampal loop extend to approximately half a theta cycle (∼60 ms), substantially exceeding passive conduction time (Mizuseki et al., 2009). These delays render gamma-period timescales (∼10–20 ms) ill-suited for precise phase alignment across the full circuit. By contrast, the ∼120 ms period of theta provides a temporal window sufficiently wide to tolerate these delays, enabling coordination across extended anatomical loops (Buzsáki et al., 2004; Zhou et al., 2022).

Importantly, theta does not impose uniform entrainment across regions. Instead, it can support computational independence by defining broad temporal windows within which local circuits operate semi-autonomously. Mizuseki et al. (2009) proposed that theta cycles provide temporal offset windows of approximately 50–80 ms, allowing subregions of the hippocampus to engage in local processing without requiring moment-to-moment synchronization with afferent input. Consistent with this view, CA1 place cells and neuronal assemblies continue to exhibit structured activity even when both CA3 and medial entorhinal inputs are silenced (Zutshi et al., 2022). These findings indicate that local circuit dynamics can persist independently of upstream drive, with theta providing global temporal organization rather than direct control.

The interaction between theta and gamma further clarifies how the hippocampus supports both global coordination and local computation. Phase–amplitude coupling between theta phase and gamma-band activity (Canolty and Knight, 2010) reflects the hierarchical organization of network dynamics rather than frequency-based multiplexing. Within the Energy Cascade framework, theta and gamma occupy distinct roles: theta reflects slow dynamics generated by extended anatomical loops and long integration times, while gamma reflects fast, state-dependent excitation–inhibition kinetics within local circuits that scale with synaptic drive. Coordination therefore arises from macro-scale temporal structure rather than millisecond-scale synchrony. This distinction reframes classical binding accounts without invoking precise spike synchrony. Theta provides a permissive temporal reference that supports iterative computation across circuits, while leaving the content and timing of local spiking determined by circuit-specific dynamics (Bland et al., 2007; McNaughton et al., 2006). In this sense, theta organizes when computation can occur, not what is computed.

Although theta power varies systematically with behavioral state, reliably increasing during active behaviors relative to quiescence (Vanderwolf, 1969), disrupting theta, or even removing the hippocampus, does not abolish those behaviors. This dissociation demonstrates that theta neither causes nor represents specific actions but instead reflects a shift in large-scale network coordination associated with active states (McFarland et al., 1975; Sławińska and Kasicki, 1998). Theta is therefore best understood as a marker of global temporal organization rather than a carrier of task-specific information.

### 4.3 Energy cascade and the power spectral density

The energy cascade framework motivates a different interpretation of hippocampal power spectral density (PSD) than conventional oscillator models. Oscillator accounts treat the PSD as a sum of separable rhythmic generators operating in parallel (Colgin, 2016). The cascade view instead treats spectral structure as an expression of activity flow through a multiscale, driven, dissipative circuit. When synaptic drive is injected at large spatial and temporal scales, for example via medial septal and entorhinal inputs (Buzsaki, 2002), it need not remain confined to the theta band. Interactions across nested circuit scales redistribute activity toward faster timescales, in qualitative analogy to cross-scale transfer in other driven systems (Kolmogorov, 1941; Richardson, 1922; Zakharov et al., 1992). This redistribution terminates at microscopic scales where synaptic transmission and action potential generation impose metabolic cost.

A direct consequence is that high-frequency activity, including gamma, is not treated as an independent oscillator or a pathway-specific communication channel. In the cascade interpretation, gamma is a dissipative signature whose magnitude is constrained by low-frequency drive. Gamma power is therefore expected to covary statistically with variables that index energetic input, including theta power and behavioral engagement, while retaining substantial variability driven by local architecture, inhibitory kinetics, and stochastic synaptic dynamics. The observable signature of this process is a systematic reshaping of PSD form with behavioral state. During low engagement, hippocampal LFP often approximates a power-law spectrum, $S\left(f\right)\propto f^{-\alpha }$, with α≈2, consistent with scale-free background structure that has been discussed in the context of criticality and related operating regimes (Bak et al., 1988; Beggs and Plenz, 2003). With increasing behavioral drive, theta power increases and the PSD slope flattens, with α decreasing from roughly 2 toward 1.4–1.7 (Sheremet et al., 2019). In this view, the tilt reflects redistribution of activity from slow forcing scales into progressively faster structure, extending through the gamma range and toward higher-frequency dissipation processes (e.g., spiking above 200 Hz).

**Fig. 9:**
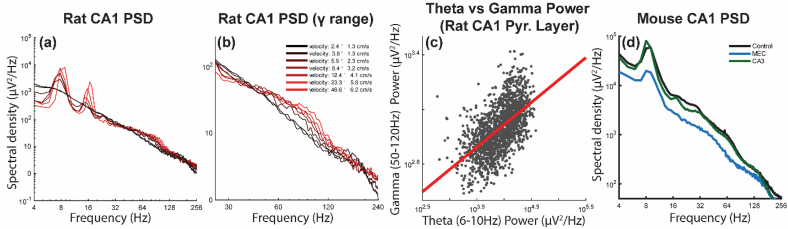
**Energy cascade predictions, quantification, and falsification.** (a,b) Prediction. Power spectral density (PSD) of rat CA1 local field potentials recorded from the pyramidal layer during locomotion. As behavioral drive increases with running speed, the spectral slope systematically flattens, reflecting redistribution of power from low frequencies into theta harmonics and broadband gamma. Data adapted from Zhou et al. (2019). (c) Quantification. Scatter plot of theta (6–10 Hz) versus gamma (50–120 Hz) power in the rat CA1 pyramidal layer. Gamma amplitude scales linearly with theta power (p = 0.02), consistent with high-frequency activity reflecting the magnitude of low-frequency energetic drive rather than an independent oscillatory generator. Data adapted from Sheremet et al. (2019). (d) Falsification. Mouse CA1 power spectra during pathway-specific optogenetic inactivation. Silencing CA3 input fails to reduce power in the 20–50 Hz range commonly labeled “slow gamma” (p > 0.2), contradicting models that assign this band to a dedicated CA3 routing channel. In contrast, removal of entorhinal input produces a broadband reduction of spectral power, including the 20-50 Hz range, consistent with reduced energetic drive rather than frequency-specific gating. Data adapted from Zhao et al. (2026).

Within this framing, gamma can be described as a spectral front that advances into higher frequencies as drive increases. This is consistent with a bottleneck in which activity accumulates at intermediate frequencies faster than it is dissipated at the smallest scales. The result contrasts with models in which gamma appears as a discrete oscillator that switches on at a specific frequency or pathway. Instead, it reflects continuous, state-dependent reshaping of the spectrum by multiscale circuit dynamics (Fig. 9).

**Fig. 10:**
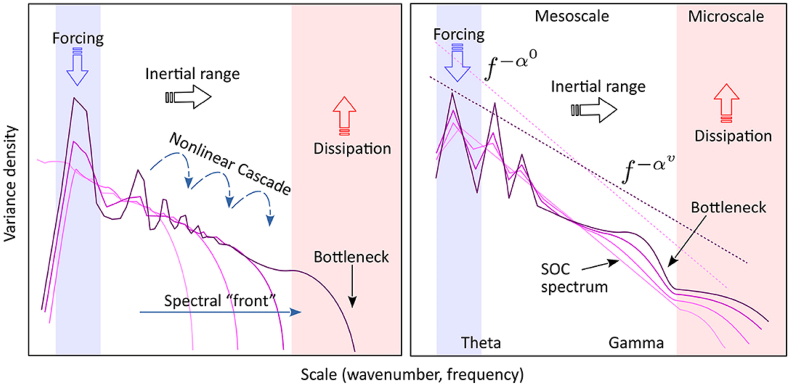
**Energy cascade interpretation of hippocampal power spectra.** Left: Conceptual schematic of an energy cascade in neural tissue. Energy is injected at large spatial and temporal scales through network-level inputs, redistributed across intermediate scales via nonlinear circuit interactions, and ultimately dissipated at small scales through synaptic transmission and action potential generation. Limited dissipation capacity at the smallest scales can lead to transient accumulation of power at intermediate frequencies, producing a spectral bottleneck. Right: Schematic depiction of hippocampal PSD across behavioral states. Low-activity conditions are characterized by a steep, approximately power-law spectrum consistent with a background driven by weak input. Increasing behavioral engagement enhances low-frequency drive, producing a progressive redistribution of power toward higher frequencies and a flattening of the spectral slope. Broadband gamma emerges as part of this continuous redistribution, consistent with a spectral front advancing toward dissipation scales rather than activation of discrete oscillatory channels. The spectral slope reflects the state of cross-scale energy transfer rather than a separable background noise process. Reproduced with permission from Sheremet et al. (2018).

These predicitons have been quantitatively tested previously (Fig. 9). In freely behaving rats, gamma power scaled linearly with theta power, with theta explaining approximately 26% of the variance in gamma amplitude in stratum radiatum (r=0.51, p<0.05; Sheremet et al., 2019). Under causal perturbation in mice, changes in theta harmonic structure following entorhinal inactivation accounted for approximately 53% of the variance in gamma reduction (r=0.73, p=0.0006; Zhao et al., 2026). We interpret the stronger coupling under perturbation as a consequence of imposing large changes in energetic input, which amplifies the cross-scale dependence expected in a driven, dissipative system (Fig. 10).

Zhou et al. (2019) further reported that state transitions reshape spectral power in a manner consistent with coupled-oscillator and entrainment principles (S. H. Strogatz, 1994; Wiener, 1965, 1966). At low velocities, entrainment is weak and power is broadly distributed across the 20–50 Hz range that is often labeled “slow gamma.” With increasing running speed, septal and entorhinal drive increases entrainment, concentrating power into theta and its harmonics and reducing power in inter-harmonic intervals. Under this interpretation, reductions in 20–40 Hz power need not reflect selective suppression of a dedicated channel. They can follow the system moving from a weakly entrained regime to a more strongly ordered regime.

This framing provides an explanation for state-dependent spectral changes without invoking an external frequency-selective controller. For example, Kemere et al. (2013) report that power in the 20–50 Hz range decreases during running, a finding they attribute to cholinergic suppression of CA3 inputs by the medial septum. However, observations of the spectral evolution of hippocampal LFP across running speeds reveal a simpler account (Sheremet et al., 2019). At low speeds, the LFP spectrum approximates a featureless power-law background (e.g., an approximate relationship where Amplitude = 1/f), consistent with a self-organized critical state in which neural activity is weakly correlated and energy is distributed broadly across all frequencies. As running speed increases, neurons become progressively entrained to theta, concentrating spectral energy into the theta peak and discrete harmonics while depleting the broad background that previously filled the inter-harmonic frequencies. Concurrently, interneuron-network oscillation frequency increases with excitatory drive, shifting gamma-range energy from ∼25 to 30 Hz toward ∼60–80 Hz as drive strengthens (Traub et al., 1996). The 20–50 Hz range is thus caught between these two reorganizing processes (theta and gamma in this instance): it loses its broadband background energy as neurons entrain to organized rhythms (neurons are modulated by both the slow theta and the faster gamma rhythms; Zhou et al., 2019). The net result, an apparent decrease in 20–50 Hz band power during fast running, is a predictable consequence of spectral reorganization under the energy cascade (Sheremet et al., 2019), not evidence for a frequency-selective suppression mechanism. This is a simple extension of the Wiener-Strogatz model. In their model, allowing neurons to interact with each other, a general frequency of entrainment is selected, accompanied by accretion of power in one band with erosion of power in the adjacent bands (Wiener, 1965, 1966; Strogatz, 1994, Strogatz, 1994). In our extension, neurons are heavily entrained to the macro scale (theta) and the local dynamics (gamma, 50-120 Hz), with the power spectra potentially eroding at 25-50 Hz. It is worth noting that Kemere et al. (2013) report a decrease in slow gamma with velocity. This observation creates additional difficulties for the spectral parcellation framework. If the interplay between gamma frequency inputs determines spike timing precision (Fernandez-Ruiz et al., 2023), then decreasing slow gamma power during fast running should degrade that precision. The opposite is observed: place fields sharpen, phase precession tightens, and theta sequences become more organized with increasing running speed (Maurer et al., 2012). Furthermore, the Kemere et al. (2013) result undermines the proxy interpretation of slow gamma. CA3 neurons remain active during fast running, the CA3-CA1 anatomical projection is intact, and CA1 spatial coding improves. Yet the signal supposedly indexing CA3-CA1 engagement weakens. Slow gamma cannot serve as a reliable proxy for CA3-CA1 communication if it fails during the behavioral condition where that pathway is most functionally relevant.

Cholinergic modulation clearly shapes circuit dynamics, but the cascade framework predicts that increased energetic drive and increased entrainment will reshape the power spectral density in a structured way, including changes that appear as selective reductions in intermediate bands, even without a dedicated routing mechanism. Notably, reports of 20–50 Hz power changes with running speed are contradictory across laboratories - increasing (Chen et al., 2011), decreasing (Kemere et al., 2013), or unchanged (Zheng et al., 2015) - as expected for a measure that conflates broadband background energy, theta harmonic structure, and shifting gamma frequencies within a fixed spectral window. Ahmed and Mehta (2012) proposed that a single gamma frequency shifts continuously upward with running speed, consistent with the cascade prediction and inconsistent with discrete, pathway-specific gamma bands.

### 4.4 Testable predictions and falsifiability of the energy cascade framework

The energy cascade framework imposes explicit, testable constraints on how spectral structure behaves across circuit states, behavioral conditions, and experimental perturbations. Unlike frequency-parcellation or multiplexing accounts, which often accommodate a wide range of outcomes post hoc, the cascade framework makes concrete predictions about when gamma activity emerges, how it scales with network state, and how perturbations propagate through the spectrum. These predictions differ in kind, not degree, from those of independent-oscillator frameworks, and each is falsifiable using existing experimental tools.

#### 4.4.1 Prediction 1: Gamma power covaries with low-frequency energetic drive

If gamma activity reflects cross-scale redistribution rather than an independently generated rhythm, its amplitude should covary with power at other frequencies. In hippocampus, this typically appears as scaling with slow activity such as theta during exploration or sharp-wave activity during rest. The framework does not require a strictly unidirectional cascade, only that gamma depends on energetic input from larger-scale processes. This input is typically organized by slow oscillations in intact circuits, but the framework does not require the drive to be oscillatory. Tonic excitation, such as pharmacological activation in vitro (Fisahn et al., 1998; Whittington et al., 1995) or sustained sensory input in visual cortex, is sufficient to recruit local E/I dynamics and produce gamma-range activity. The critical variable is the magnitude of synaptic drive, not its temporal structure.

Regions characterized by strong, punctate synaptic drive, such as CA3 and the dentate gyrus, exhibit greater theta waveform asymmetry and consequently more prominent harmonic power in the 20–50 Hz range. This accounts for the prevalence of ‘slow gamma’ reports in these regions (Zhou et al., 2019) without requiring the postulation of region-specific low-frequency oscillators. In dentate gyrus, theta and harmonic coupling extends as high as 64 Hz (Sheremet et al., 2020), reflecting the energetic cascade from asymmetric theta waveforms rather than independent gamma generation.

*Empirical* support*.* In freely behaving rats, gamma power scales linearly with theta power (Fig. 9C; r = 0.51, p < 0.05; Sheremet et al., 2019). Under causal perturbation, this relationship strengthens: changes in theta harmonic structure following entorhinal inactivation account for a substantial fraction of gamma variance (r = 0.73, p = 0.0006; Zhao et al., 2026). Across conditions, gamma does not appear in isolation but covaries with activity at other frequencies.

*Falsification.* The cascade framework would be falsified if sustained, high-amplitude, narrow-band gamma reliably emerged across trials and behavioral states while low-frequency power and total system energy were reduced or absent. Reproducible expression of gamma as an independent dominant mode, rather than transient or state-dependent events, would contradict an energy-dependent account and support independent-oscillator models.

#### 4.4.2 Prediction 2: Gamma properties shift continuously with circuit state

If gamma-band activity reflects the dissipation of synaptic drive through local circuits rather than the activation of discrete oscillators, then its spectral expression should shift smoothly as a function of network state. Manipulations of excitability or inhibitory kinetics should produce continuous changes in gamma frequency, bandwidth, or amplitude. Discrete appearance or disappearance of fixed gamma bands is not expected.

Apparent gamma “bands” should vary continuously with input strength and excitation–inhibition balance across behavioral conditions, arousal states, and task demands. Reports of slow-, mid-, or fast-gamma should collapse onto a continuous distribution when analyzed across conditions rather than forming sharply separable clusters (Douchamps et al., 2024).

*Empirical* support*.* Optogenetic silencing of medial entorhinal cortex produces proportional reductions across the spectrum rather than selective elimination of a putative “fast gamma” band (60–100 Hz; Zhao et al., 2026). Conversely, CA3 inactivation, predicted by routing models to abolish “slow gamma” (30–50 Hz), produces no significant reduction in any gamma range (Zhao et al., 2026). Natural variations in behavioral state yield continuous, correlated modulation of theta and gamma power rather than band switching (Zhou et al., 2022).

*Falsification.* Selective elimination of one gamma band with preservation of another following manipulation of a single pathway would contradict continuous-state predictions and support discrete oscillator accounts.

#### 4.4.3 Prediction 3: Cross-frequency coupling scales with network energetic state

If gamma activity reflects local circuit responses to structured synaptic drive rather than independent oscillatory generators, then changes in gamma power, bandwidth, and intermittency should systematically covary with nonlinear interactions between slow and fast timescales. Cross-frequency coupling strength should vary continuously with behavioral and metabolic state, not switch categorically between coordination modes. Specifically, gamma structure should strengthen when slow rhythms exhibit increased asymmetry, sharper transitions, or enhanced cross-scale coupling, and weaken when such structure is reduced, even when mean firing rates or overall spectral power are held constant. The circuit-level mechanism underlying this prediction has been characterized: increased behavioral drive elevates excitatory input from CA3 and entorhinal cortex to CA1, increasing interneuron firing rates and shifting gamma frequency upward in proportion to drive strength (Ahmed and Mehta, 2012; Traub et al., 1996). Under this framework, gamma is not explained by waveform shape alone but is constrained by the same nonlinear dynamics that shape it.

*Empirical* support*.* Theta–gamma coupling strength increases monotonically with running speed (Sheremet et al., 2019; Zhou et al., 2022). High-drive states such as running exhibit stronger theta–gamma coupling than low-drive states such as immobility.

*Falsification.* Reproducible switching between high and low coupling states that is unrelated to behavioral drive or metabolic demand would contradict an energy-dependent account.

#### 4.4.4 Prediction 4: Perturbations propagate hierarchically across frequencies

If oscillatory structure reflects hierarchical energy transfer, then experimental or pathological perturbations should affect multiple frequency scales in a coordinated manner rather than selectively abolishing isolated bands. Experimental manipulations that flatten or desynchronize low-frequency activity (such as disrupting theta, alpha, or beta structure) should reduce or destabilize gamma-band organization, even if local circuitry remains intact. In contrast, models that posit independent gamma channels predict relative preservation of gamma structure under such manipulations. As a concrete example, consider optogenetic silencing of the medial septum, which abolishes theta while leaving CA3 and entorhinal circuits intact. The cascade predicts proportional broadband degradation of gamma with no selective preservation of individual bands. Parcellation models predict that pathway-specific gamma signatures should persist, albeit reduced, because their upstream generators remain intact. Similarly, replacing rhythmic septal input with tonic excitation should, under the cascade framework, produce broadband increases across all frequencies without the emergence of discrete pathway-specific bands.

*Empirical* support*.* Systematic energy reduction via barbiturate overdose produces hierarchical spectral degradation, with gamma degrading first followed by progressively slower frequencies and correlated decay rates across the spectrum (Zhou et al., 2021). Optogenetic circuit perturbations similarly affect theta and gamma proportionally rather than selectively (Zhao et al., 2026).

*Falsification.* Conditions that selectively abolish gamma activity while preserving normal theta amplitude, theta–spike relationships, and population firing would falsify hierarchical organization.

#### 4.4.5 Prediction 5: Species-level scaling preserves cascade dynamics while shifting characteristic frequencies

A common challenge to theta-based coordination models is the reduced prominence of continuous, high-amplitude theta oscillations in primates compared to rodents (Ekstrom et al., 2005; Jutras et al., 2013; Killian et al., 2012). Within the energy cascade framework, this difference is expected and reflects anatomical scaling rather than mechanistic divergence. As brain size and conduction distances increase, the dominant low-frequency organizing rhythm must shift toward slower timescales to maintain large-scale coordination. Gamma-band activity, constrained by local synaptic kinetics and excitation–inhibition balance, remains comparatively conserved across species.

The framework predicts that in primates, gamma activity should preferentially couple to slower low-frequency structure, such as delta or low-alpha rhythms, rather than to a fixed rodent-defined theta band. The apparent reduction of continuous theta in primates does not reflect a loss of the organizing principle, but a biophysical rescaling of the temporal window required for coordination in larger brains. The hierarchical relationship between large-scale organization and local dissipation is preserved, even as the characteristic frequencies shift to satisfy the constraints imposed by anatomy.

*Falsification.* If gamma structure in primates remains robust and stable in the absence of ANY slow-frequency organization, or if gamma-slow frequency coupling does not increase with task demands or arousal state, the energy-dependent account would be challenged. To falsify the cascade, it must be demonstrated that gamma can be selectively amplified or modulated by a mechanism that does not involve increasing energy throughput. However, such a demonstration would require identifying a source of increased synaptic drive that leaves no signature in slower frequency bands.

## 5 Extension beyond hippocampus: constraints, predictions, and open questions

The arguments developed in this review are grounded in hippocampal physiology because its laminated architecture and well-characterized circuitry allow biophysical constraints to be evaluated with unusual clarity. This focus does not imply that the hippocampus operates under unique coding principles. The central claim of the energy cascade framework is that spectral structure reflects how synaptic energy is distributed and dissipated under conserved physical constraints. That claim does not depend on hippocampus-specific mechanisms. It rests on principles that apply to all excitable tissue, including current conservation, impedance, synaptic time constants, and excitatory–inhibitory balance. This framing yields a clear expectation; if oscillatory structure arises from generic circuit physics rather than frequency-specific communication channels, then certain features such as cross-frequency coupling, harmonic structure, and laminar source–sink organization should generalize across brain regions. At the same time, their precise expression should vary systematically with anatomy, connectivity, and input statistics. In this section, we outline how the energy cascade framework extends beyond the hippocampus, identify predictions that distinguish it from spectral parcellation models, and highlight open experimental questions that follow directly from this view.

### 5.1 The cortical binding by rate enhancement theory

Consistent with the energy cascade theory, the binding by rate enhancement (BBRE) theory addresses several persistent anomalies that challenge synchronization-based and spectral parcellation models. While gamma-based communication theories struggle to account for low inter-areal coherence values, inconsistent frequency ranges across species, and weak causal evidence, BBRE provides a parsimonious alternative grounded in firing rate modulation rather than precise temporal alignment. In this framework, features are bound when their neural representations exhibit enhanced firing rates (Roelfsema and Houtkamp, 2011).

We interpret this proposed “labeling” not as a symbolic tag or frequency marker, but as synaptic leverage. Rate enhancement increases temporal summation within the integration window of downstream neurons, thereby increasing transmembrane current flow and the probability of postsynaptic firing (Histed and Maunsell, 2014). This mechanism allows distributed neural populations to influence downstream targets without requiring stable phase locking or millisecond-precision spike timing, avoiding the biophysical constraints imposed by axonal conduction delays and synaptic variability.

BBRE rests on the well-established observation that meaningful neural signaling depends primarily on firing rates rather than precise spike timing (Shadlen and Movshon, 1999). Experimental evidence demonstrates that cortical populations integrate synaptic inputs over extended temporal windows of approximately 100 ms. In a series of causal experiments, Histed and Maunsell (2014) showed that behavioral detection performance depended solely on total spike count within this window. Concentrating spikes into brief synchronous bursts of 1–3 ms conferred no behavioral advantage over distributing the same number of spikes across the full integration period, even when stimuli were delivered at frequencies spanning beta through gamma (10–50 Hz). These results indicate that downstream neurons sum inputs based on aggregate activation rather than oscillatory phase or temporal compression.

Critically, the behavioral effects in these experiments arose from small rate increases distributed across large neuronal populations, on the order of approximately 1 spike per second per neuron, rather than from strong synchronous events. This pattern supports a rate-based coding scheme in which population-level covariance reflects shared input and network state, not precise synchrony. Consistent with this view, Romo and Salinas (2003) demonstrated that in somatosensory decision-making tasks, firing rates across multiple cortical areas varied systematically with stimulus properties and decision demands. These rate modulations reflect circuit-level responses to task constraints rather than the encoding of abstract variables through temporal codes.

BBRE does not posit global or indiscriminate gain increases. Rate enhancement is conditional on existing feedforward activation and is selectively gated by top-down feedback and local disinhibitory circuits, including VIP-mediated motifs. As a result, increased firing remains confined to neurons already participating in task-relevant representations, supporting object- and feature-specific binding without reliance on millisecond-scale synchrony (Roelfsema, 2023). This conditional gating avoids the loss of specificity that would accompany uniform gain modulation.

This framework builds on Hebbian principles in which coincident activation strengthens functional connectivity between neurons (Gotts et al., 2012; Hebb, 1949; von der Malsburg, 1999). Attention-dependent increases in firing rates are associated with changes in effective connectivity, typically measured as trial-to-trial covariation in firing rather than precise spike synchrony. Reynolds and Desimone (2003) showed that attention modulates response gain in area V4, effectively biasing functional interactions between neurons representing attended versus unattended stimuli. Similarly, object-based attention studies demonstrate that enhancing the firing rate of neurons representing one feature of an object leads to enhanced processing of its other features, consistent with integrated representations achieved through coordinated rate modulation (O'Craven et al., 1999; Roelfsema et al., 2000).

BBRE also aligns naturally with the Energy Cascade framework described in Section 4.1. Increased energetic drive at large scales leads to elevated firing rates across local circuits, rather than to the selective activation of frequency-specific channels (Buzsáki et al., 2004). Empirically, firing rates increase with arousal, attention, and locomotion, paralleling broad increases in oscillatory power (McGinley et al., 2015; McNaughton et al., 1983; Shen et al., 1997). These changes reflect shifts in global excitability and synaptic drive rather than discrete computational operations assigned to particular frequencies.

The cascade from slow to fast dynamics facilitates the emergence of locally elevated firing rates through recurrent excitation and feedback inhibition (Douglas et al., 1995; Sheremet et al., 2019). Both BBRE and the energy cascade emphasize coincident activation arising from shared input and network state, rather than sustained phase locking across time. Coincidence in this context refers to transient co-activation within broad integration windows, not the millisecond-precision simultaneity required by synchrony-based theories (Shadlen and Movshon, 1999; Buzsáki et al., 2004).

The relationship between firing rate and oscillatory structure follows directly from this hierarchy. Slow network-driven rhythms such as theta reflect large-scale synaptic current flow. When excitatory postsynaptic potentials fluctuate rhythmically, firing rates increase in sufficiently driven neurons, producing correlations between spiking and oscillatory phase without requiring phase-coded information transmission. Within this framework, correlations between firing rate and memory performance reflect increased circuit engagement rather than temporal coding per se (Carr et al., 2011; Lepage et al., 2000).

BBRE therefore resolves several longstanding problems faced by binding-by-synchrony and communication-through-coherence models. These models require near-zero phase lag or sustained phase relationships to support binding and routing, assumptions explicitly stated in early formulations (Crick & Koch, 1990, 2003; Singer and Gray, 1995) but rarely demonstrated consistently in vivo (Ray and Maunsell, 2015). BBRE accounts for binding without invoking precise spike timing, consistent with critiques of temporal binding that emphasize its limited explanatory power (Di Lollo, 2012; Roskies, 1999).

By emphasizing firing rate modulation, conditional gating, and circuit-level integration, BBRE provides a biologically plausible mechanism for binding, attention, and perceptual organization that does not depend on discrete oscillatory channels or millisecond-scale synchrony. In conjunction with the energy cascade framework, it supports a view in which oscillatory structure reflects the state-dependent redistribution of synaptic drive across scales, rather than serving as a symbolic code or routing label for information.

### 5.2 Conserved biophysical constraints on oscillatory structure

Neurons, regardless of location or function, are excitable membranes embedded in conductive tissue and governed by the same physical laws of current flow, charge conservation, and synaptic kinetics. From this perspective, oscillatory structure should not be understood as a collection of region-specific codes, but as an emergent consequence of how energy enters, propagates through, and is dissipated by neural circuits. Current conservation and electroneutrality impose fundamental limits on how activity can be localized. Any synaptic current entering a neuron must be balanced by return currents distributed across the membrane and extracellular space. As a result, oscillatory activity, whether generated dendritically or perisomatically, necessarily produces spatially distributed field patterns with complementary source–sink structure. This constraint applies equally to hippocampus, neocortex, thalamus, and cerebellum. It precludes the existence of oscillations that are both frequency-specific and anatomically isolated in the manner often implied by spectral parcellation models.

Extending the energy cascade framework beyond hippocampus requires confronting a measurement problem, not a mechanistic one. The hippocampus is often treated as a model system because its architecture makes certain dynamics unusually accessible to extracellular recording. The highly aligned dendritic geometry of pyramidal neurons, combined with laminar segregation of afferent pathways, produces large, coherent open-field dipoles that sum efficiently in the extracellular space. This alignment enables relatively unambiguous current source density (CSD) analyses and facilitates precise localization of synaptic drive. Neocortical circuits operate under different geometric and organizational constraints. Cortical columns are distributed across sulci and gyri with diverse orientations, such that dipoles generated by neighboring populations may partially cancel in aggregate field recordings (Lindén et al., 2011; Nunez and Srinivasan, 2006). Extracellular signals in cortex reflect the superposition of multiple interacting processes rather than a single dominant generator. This does not imply weaker coordination. Coordination is simply less likely to manifest as sharply defined spectral peaks (S. Strogatz, 1994; Wiener, 1965, 1966). Hippocampal theta provides a strong, continuous, behaviorally coupled temporal scaffold that organizes higher-frequency activity and promotes harmonic structure. Neocortical rhythms have been described as more spatially localized, intermittent, and strongly modulated by task demands, attention, and arousal (Wang, 2010). Without a persistent global envelope or strong synaptic drive, higher-frequency components exhibit less stable phase relationships and fewer narrow-band spectral signatures, even when governed by identical excitatory–inhibitory dynamics (Churchland et al., 2010; Nauhaus et al., 2009; Raut et al., 2021).

Conserved cascade-like dynamics express themselves in cortex as broadband spectral tilts, scale-free fluctuations, or transient population events (Beggs and Plenz, 2003; Petermann et al., 2009) rather than sustained oscillations with fixed frequency boundaries. This reflects a difference in observability, not in underlying principles (a distinction often collapsed in studies that equate the absence of spectral peaks with the absence of coordination). The relevant question is not whether a region implements an energy cascade, but whether circuit geometry and dynamical state allow that cascade to be resolved spectrally.

Apparent differences between hippocampal and neocortical rhythms therefore arise from differences in circuit architecture and measurement access rather than from fundamentally different coding strategies. Researchers must distinguish between biological mechanism and observability. A lack of narrow-band peaks in the neocortex does not necessarily invalidate the energy cascade; rather, geometrically complex tissue may simply hinder the recording of coherent field potentials.

## 6 Conclusions, open questions and future directions

The study of neural oscillations rests on assumptions that require critical examination. Frequency-specific interpretations, particularly the characterization of gamma rhythms as distinct communication channels, often rely on phenomenological correlations that lack sufficient biophysical grounding. “Spectral phrenology” assigns cognitive roles to isolated frequency bands without specifying the mechanisms that would selectively generate, maintain, or decode those bands (Buzsáki et al., 2004). Theories such as Binding by Synchrony and Communication Through Coherence identify correlations between rhythmic alignment and interregional coupling but correlation does not establish mechanism. The characterization of “slow gamma” as a distinct rhythm may result from artifacts introduced by analysis windows or decomposition techniques. Current interpretations often neglect biophysical realities, dendritic filtering, electroneutrality, impedance gradients, that challenge simplistic mappings of oscillatory phenomena to discrete layers or pathways.

The energy cascade framework provides an alternative grounded in biophysical constraints. Energy flows from low-frequency oscillations such as theta to higher frequencies like gamma, with cross-frequency coupling emerging as a natural consequence of this redistribution (Sheremet et al., 2019). Theta oscillations coordinate neural dynamics at large scales while gamma reflects local dissipation under excitation–inhibition balance (Buzsaki, 2002). Binding by rate enhancement theory complements this view, emphasizing firing rate modulations as a robust mechanism for feature binding and network integration without requiring precise synchrony (Roelfsema and Houtkamp, 2011). These perspectives converge on the brain's emergent, multiscale dynamics rather than on frequency labels. Reframing oscillatory structure as an emergent consequence of energy dissipation sharpens inquiry rather than closing it off. The energy cascade framework generates testable experimental questions that are mechanistically grounded in biophysics rather than centered on frequency labels and highlight future avenues of empirical interrogation.

Neuromodulatory systems strongly alter membrane conductances, synaptic gain, and inhibitory tone, the parameters that govern cascade dynamics. Do changes in neuromodulatory state reshape spectral structure continuously or toggle discrete oscillatory modes? The framework predicts continuous shifts: power distributions and harmonic organization change gradually as cholinergic, noradrenergic, or dopaminergic input modulates excitatory–inhibitory balance. Systematic manipulation of neuromodulatory input while tracking waveform geometry and nonlinear coupling tests whether oscillatory “bands” reflect circuit state transitions or gradual rebalancing under constraint.

Septal manipulations offer a direct test of the cascade framework as the medial septum is one of the primary pacemakers of hippocampal theta. The framework makes specific predictions for two classes of manipulation. Inhibiting septal cholinergic drive, which reduces theta amplitude, should produce proportional broadband reductions in gamma power without selective preservation of individual gamma bands. Graded septal cooling, which progressively slows and weakens theta (Petersen and Buzsáki, 2020), should produce hierarchical spectral degradation consistent with reduced energetic throughput. In each case, the cascade predicts coordinated, proportional changes across the spectrum, whereas independent-oscillator models predict relative preservation of gamma-band structure. We note, however, that forced-frequency paradigms such as optogenetic septal pacing require cautious interpretation, as artificial stimulation can produce field potentials that resemble physiological theta while profoundly disrupting the underlying cellular dynamics (Scarlett et al., 2004).

The energy cascade framework ultimately requires metabolism and bioenergetics as its source. If oscillatory structure reflects energy redistribution through neural tissue, then spectral dynamics should couple to metabolic demand and respiratory oscillations that follow oxygen delivery. Recent evidence demonstrates that hippocampal theta phase-locks to respiration (Lockmann et al., 2016; Tort et al., 2018), and respiratory-locked modulation extends to gamma-band activity and single-unit firing (Yanovsky et al., 2014). This coupling is not incidental. It provides a direct mechanistic link between the abstract concept of “network energy” and the concrete biochemistry of ATP synthesis, glucose metabolism, and oxygenation. The framework predicts that experimental manipulations of metabolic state (hypoxia, glucose availability, metabolic inhibitors) should produce graded, hierarchical changes in spectral structure consistent with reduced energetic throughput. Such experiments test whether “energy cascade” is metaphorical or reflects actual thermodynamic constraints on neural activity.

What is the simplest network motif capable of producing cascade-like redistribution of spectral energy? Does such behavior require full recurrent inhibition, specific synaptic delays, or generic nonlinear feedback? Targeted perturbations in vitro circuits or computational models address whether cascade dynamics represent a specialized feature or a generic property of excitable networks under constraint. The answer to this question would clarify how broadly the framework applies across brain regions and species.

This framework has direct implications for understanding cognitive function and psychiatric disease. Oscillatory biomarkers are increasingly proposed as diagnostic tools and therapeutic targets for conditions including Alzheimer's disease and schizophrenia (Palop and Mucke, 2016; Uhlhaas and Singer, 2010). If the field's understanding of oscillatory organization is built on analytical artifacts and unfalsifiable frequency-function assignments, then clinical interventions derived from that understanding – specifically, attempts to restore specific gamma bands, enhance particular cross-frequency coupling motifs, or target isolated spectral features - risk pursuing mechanisms that do not exist. The cascade framework suggests that therapeutic interventions should instead target the restoration of energy flow across scales or the rebalancing of excitatory-inhibitory interactions, acknowledging the nonlinear, emergent properties of brain dynamics rather than treating frequency bands as discrete, static entities. The obligation to get the basic science right before translating it is not abstract.

Nested recurrent structure is not unique to neural systems. It is the inevitable consequence of sustained drive coupled with dissipation in any nonlinear medium. Richardson's observation, originally applied to atmospheric turbulence, formalizes a general constraint: energy injected at large scales propagates through coupled subsystems until dissipation terminates further structure formation. In neural tissue, excitation represents ongoing activity (spiking, depolarization, recurrent amplification); coupling links smaller loops to larger ones through shared circuitry and membrane dynamics; dissipation encompasses synaptic failures, subthreshold responses, cable filtering, and metabolic limits. This framework makes no assumptions about oscillatory function, coding schemes, or frequency-specific mechanisms. It predicts only that if recurrent loops exist across spatial scales with finite conduction velocity and local energy loss, then measured field potentials will reflect hierarchically coupled dynamics rather than independent narrowband generators. This prediction is falsifiable: it would be contradicted by scale-invariant coherence, temporally stable high-frequency rhythms independent of slower dynamics, or frequency bands that operate as autonomous functional modules.

## CRediT author contribution statement

Cristina Besosa: Conceptualization; Literature search; Synthesis and interpretation of literature; Visualization; Writing – Original Draft; Writing – Review & Editing.

Yu Qin: Formal analysis; Visualization; Writing – Review & Editing.

Sara Burke: Supervision; Conceptualization; Literature search; Funding acquisition; Writing – Review & Editing.

Andrew Maurer: Supervision; Conceptualization; Literature search; Visualization; Funding acquisition; Writing – Original Draft; Writing – Review & Editing.

All authors approved the final version of the manuscript.

## Declaration of generative AI and AI-assisted technologies in the writing process

During the preparation of this work the authors used Claude.ai by Anthropic in order to improve readability. After using this tool/service, the authors reviewed and edited the content as needed and take full responsibility for the content of the publication.

## Funding source

This work was supported by the McKnight Brain Research Foundation the UF Center for Cogntive Aging and Memory, and NIH grants- Grant Sponsor: National Institute of Mental Health; Grant Number: R01MH126236 and a Diversity Supplement to NIH grant 3R01MH126236-03S1.

## Declaration of competing interest

The authors declare that they have no known competing financial interests or personal relationships that could have appeared to influence the work reported in this paper.

## Data Availability

Data will be made available on request.
