# Supplementary material for: Spectral dependence as a framework for neural coordination
Source: Curr Res Neurobiol. 2026 May 11;10:100159. doi: 10.1016/j.crneur.2026.100159 (PMC13202281; doi:10.1016/j.crneur.2026.100159)
Supplement: Multimedia component 1 [file mmc1.docx]

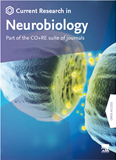
Peer Review Overview

**Manuscript Title:** Spectral Dependence as a Framework for Neural Coordination

| Received |  | Dec 10, 2025 |
| --- | --- | --- |
| 1st Decision |  | Jan 15, 2026 |
| 1st Revision Submitted |  | Feb 13, 2026 |
| 2nd Decision |  | May 27, 2026 |
| 2nd Revision Submitted |  | Apr 16, 2026 |
| Accepted |  | Apr 27, 2026 |

1st Decision letter

### **Reference: CRNEUR-D-25-00019**

### **Title:** Spectral Dependence as a Framework for Neural Coordination

### **Journal:** Current Research in Neurobiology

Dear Ms Besosa,

Thank you for submitting your manuscript to Current Research in Neurobiology. 

I have completed my evaluation of your manuscript. The expert reviewers recommend reconsideration of your manuscript following major revision and have provided extensive supportive comments to address. I invite you to resubmit your manuscript after addressing the comments below. Please resubmit your revised manuscript by **Feb 14, 2026**. 

When revising your manuscript, please consider all issues mentioned in the reviewers' comments carefully: please outline every change made in response to their comments and provide suitable rebuttals for any comments not addressed. Please note that your revised submission may need to be re-reviewed.  

To submit a revision, go to https://www.editorialmanager.com/crneur/ and log in as an Author. You will see a menu item call Submission Needing Revision. You will find your submission record there.

When you submit the revised manuscript, please provide a separate document, uploaded as 'Detailed Response to Reviewers,' that carefully details, point-by-point, the list of changes made in response to the reviewers' comments. You may also include a suitable rebuttal to any specific request for change that you have not made. The journal strongly encourages you to submit two versions of your revised manuscript in order to facilitate the evaluation process: one with changes in the manuscript marked (upload as file type: Manuscript (REVISED, text with changes Marked)), in addition to an unmarked, production-ready version (required) (upload as file type: Manuscript).

Research Elements (optional)
This journal encourages you to share research objects - including your raw data, methods, protocols, software, hardware and more – which support your original research article in a Research Elements journal. Research Elements are open access, multidisciplinary, peer-reviewed journals which make the objects associated with your research more discoverable, trustworthy and promote replicability and reproducibility. As open access journals, there may be an Article Publishing Charge if your paper is accepted for publication. Find out more about the Research Elements journals at https://www.elsevier.com/authors/tools-and-resources/research-elements-journals?dgcid=ec_em_research_elements_email.


Current Research in Neurobiology values your contribution and I look forward to receiving your revised manuscript.

Kind regards,  

Anna S Mitchell, Ph.D. 
Editor in Chief 
Current Research in Neurobiology

**Comments from Editors and Reviewers:**

**Reviewer #1:**

The origin and function of gamma oscillations remain a major source of speculation in systems neuroscience, with theories ranging widely in both mechanism and functional attribution. This review takes an important step back to reassess these ideas by explicitly asking where gamma may or may not come from, and what it may or may not do. The authors make a clear and forceful argument in favor of a spectral dependence / energy cascade framework, and systematically lay out biophysical and conceptual challenges to models that rely on anatomical and frequency-specific parcellation. This is a contentious position in the field, but an important one to articulate clearly and rigorously. Overall, I find the central thesis compelling and timely, and I support publication of this manuscript. That said, the current presentation sometimes attempts to span too many conceptual levels at once, which can obscure the core message. With improved focus and clearer schematic communication of the proposed model, this review could have substantial impact.

Major comments

1. A recurring issue throughout the manuscript is a tension between broad, cortex-wide claims about neural coordination and a much more detailed, mechanistically grounded discussion centered on the hippocampus. While the hippocampus is clearly used as an exemplar system, this distinction is not always made explicit, and at times the manuscript appears to oscillate between general principles and region-specific arguments without fully reconciling the two.
In its current form, this can feel conceptually muddled. Different mechanisms may plausibly dominate in different brain regions, and the hippocampus (given its strong lamination, prominent theta rhythm, and well-characterized circuitry) may be particularly well-suited to an energy cascade framework. I would encourage the authors to more explicitly foreground the hippocampus as the primary focus of the review, and then include a dedicated section toward the end discussing how similar principles might extend to neocortex, along with open questions and potential caveats.

2. At present, several figures are more effective at critiquing existing models than at positively conveying the authors' own framework. If the energy cascade / spectral dependence model is the key take-home message, it should be made more visually prominent.

I strongly encourage the authors to revise or add schematics that more explicitly illustrate:
* How energy flows across scales and frequencies
* How gamma emerges as a local, state-dependent consequence
* How this framework differs qualitatively from parcellation-based views
Clearer visual intuition would significantly improve the accessibility and impact of the manuscript.

For example, for the graphical abstract, Is the orange 'Y' supposed to be gamma? Personally, I find the schematic on the right describing the energy cascade quite hard to understand. Also, the left schematic is very specific for hippocampal pyramidal cells, while the right on seems to be generic across regions. It might be worthwhile to contrast the two within the same network (e.g., hippocampus)

3. Lines 357-359 make a central argument about low coherence between hippocampal layers. Given how important this point is for undermining spectral routing models, it would strengthen the manuscript to either show this directly or reproduce representative data from prior studies.
Even a brief figure panel or citation to an explicit coherence analysis would help anchor this claim empirically.

4. Does the energy cascade framework posit that, given variables such as running speed, theta power, or theta waveform shape, the emergent gamma power and frequency should be predictable? If so, it could be interesting to demonstrate this explicitly (e.g., via regression or variance-explained analyses).
Alternatively, if gamma cannot be fully predicted from these factors, it would be useful to clarify what the authors view as the dominant sources of variability. Making this explicit would help readers understand the limits and testable predictions of the framework.

5. Lines 590-592 state that "theta may provide a global temporal framework." Conceptually, this seems similar to roles that have previously been attributed to gamma in the literature. It would be helpful if the authors could more explicitly clarify how their proposal differs in substance, rather than simply shifting the coordinating role from gamma to theta.
This clarification would help avoid the impression that the same functional logic is being reassigned to a different frequency band.

6. Given the much lower and less consistent theta power in humans and non-human primates, it would be interesting to see whether the framework makes specific predictions about how gamma should differ across species. Showing that gamma is altered in predictable ways based on differences in theta structure could substantially strengthen the argument.

7. What determines the specificity of rate gain modulation in the BBRE framework? Clarifying how selective rate enhancement arises (particularly in the absence of precise synchrony) would help readers understand how BBRE avoids becoming overly global or nonspecific.

8. Given the manuscript's emphasis on falsifiability as a weakness of spectral parcellation-based theories, it would strengthen the framework to more explicitly state what empirical observations would challenge or falsify an energy cascade account. Clarifying these limits would help sharpen the proposal and guide future experimental tests.

**Reviewer #2:**

This manuscript presents an ambitious theoretical review that critically evaluates gamma frequency-specific models of neural communication and promotes an energy-cascade interpretation, in which slow, large-scale rhythms organize neural activity by cascading energy into faster, local dynamics rather than functioning as dedicated communication channels. The authors should be applauded for this effort. Constructive criticism of influential theories is crucial for progress in the field, particularly when those theories have become deeply embedded in how data are interpreted.

I believe this manuscript has the potential to become an important reference on the topic and to stimulate constructive debate. However, in its current form, some sections feel overly superficial, while others rely on citations in a way that comes across as somewhat cursory. Some key concepts are introduced in a rather hand-wavy manner, which may be challenging for readers who are not already deeply familiar with the literature. While I don't believe this is the authors' intention, parts of the discussion risk giving the impression that alternative frameworks are addressed in a somewhat straw-man fashion. Below, I go into more detail on these points.

Major comments

Major 1. Section '4.1. The Energy Cascade Model for Neural Coordination." is arguably the most important part of the manuscript, and I'd strongly encourage the authors to invest more effort in presenting this framework in a concrete and accessible way. As it stands, the discussion feels somewhat abstract and is not clearly aimed at a broad neuroscience audience, which may limit its impact. For example, sentences such as "draws from concepts in physics, particularly Kolmogorov's theory of turbulence (1941), suggesting that oscillations reflect a hierarchical process where activity propagates from larger, slower oscillations to smaller, faster ones in a cascade-like manner" are conceptually appealing, but remain difficult to translate into neural terms. Readers who are not already familiar with turbulence theory are left without a clear sense of what this cascade looks like in terms of neuronal activity and circuit interactions. It'd greatly strengthen the manuscript if the authors could provide a simple, tangible model (perhaps a schematic or worked example) illustrating how this cascade operates in a neural circuit.

Major 2. The authors frame the frequency-parcellation framework as being difficult to falsify, which is an important and well-taken point. However, this argument would benefit from being developed more explicitly. The best way to strengthen it would be to propose concrete, falsifiable tests of the theory. For example, the authors note that interareal coherence is often weak, largely observed at the LFP level, and potentially artifactual. Recent work showing that downstream pyramidal cells don't reliably follow gamma-paced inputs (PMC7616856) already challenges a strong interpretation of gamma-based "routing." If slow and mid gamma oscillations do reflect CA3->CA1 and EC->CA1 interactions, respectively, one would predict that spike-to-spike raw cross-correlations between these regions should exhibit clear components at the corresponding frequencies. Similarly, disrupting one pathway should lead to a selective reduction of its associated gamma band in the downstream circuit. The authors may have additional ideas for such tangible tests. Importantly, the same kind of falsifiable predictions should also be articulated for the proposed energy-cascade framework.

Major 3. In the section "Historical perspectives on the role of gamma rhythms in cognition," the authors raise an important concern about the risks of disconnecting oscillations measured at the electrode from the microcircuits that generate them. In the final sentence of that section, they write: "Foundational notions of gamma as a signature of E/I volleys in local networks has been overshadowed by more contemporary ideas regarding the role that gamma may play in coordinating disparate brain regions or routing the transfer of neural activity between distinct anatomical pathways." While this is an important point, it is not developed in sufficient detail, and I was left unsure about what, exactly, the authors intend by this contrast.
If proponents of frequency parcellation argue that distinct anatomical pathways give rise to different gamma frequencies, this would seem, at least in principle (albeit very superficially), to link oscillatory phenomena to underlying microcircuit properties. For example, if one region exhibits resonance properties due to its local E/I dynamics at a given frequency, while another region has a distinct microcircuit with a different resonance frequency, wouldn't the postsynaptic potentials imposed by these two inputs produce different frequency components in the LFP measured at their shared downstream target? In that sense, pathway-specific frequency hypotheses do not obviously abandon microcircuit grounding. My impression is that the authors do not intend to suggest that frequency-parcellation models ignore microcircuitry altogether, but rather that they are often imprecise or under-specified with respect to how specific circuit mechanisms give rise to specific frequencies. If this is the intended critique, it would benefit from being stated more explicitly. If instead this reflects a misunderstanding on my part, the authors may need to clarify more clearly what point is being made, as the current framing reads as somewhat vague.
A recent paper (PMC9069800) shows that suppressing SST interneurons in DG-CA3 shifts gamma-band activity in CA1 away from slow gamma, whereas suppressing DG-CA3 PV cells has the opposite effect and enhances what they call "fast gamma" (defined as 50-100Hz, which overlaps more closely with what other groups refer to as mid gamma and often associate with EC inputs). Wouldn't this represent an initial step toward grounding these ideas mechanistically? While the link to CA3 versus EC drive remains interpretational, the results suggest that different interneuron classes contribute differently to distinct gamma bands. This seems like a key piece of work that would be worth discussing in this section of the manuscript. Further, Lasztóczi & Klausberger (PMID: 24607232) have made some effort in characterizing how different interneurons within CA1 relate to different gamma bands.
This issue becomes more pronounced in light of Section 4.1 (The Energy Cascade Model for Neural Coordination), where the authors introduce the framework they favor. That section relies on language that is quite abstract for most readers, as mentioned in Major 1. For example, the statement that "In this context, 'energy' refers to the aggregate power of synaptic currents and transmembrane conductance driving oscillatory activity. The result is a directional flow of energy across temporal scales—not simply co-occurring rhythms, but a cascade from a global input (source) to a localized dissipation (sink)" is considerably less concrete than the pathway-based hypotheses it is meant to replace. I'm not arguing that the energy-cascade framework is in less physiologically grounded, but rather that the manuscript does little to explain why it should be considered more directly connected to known circuit mechanisms than "frequency-parcellation" accounts. As a result, the claimed contrast between mechanistic grounding and abstraction remains insufficiently justified and, at times, even contradictory.

Major 4. I did not find the arguments presented in Figure 1 and the associated text particularly convincing. The authors suggest that variability in reported gamma-band definitions across studies reflects a tendency for other groups to flexibly adjust frequency boundaries to fit their theoretical interpretations. However, this variability seems more likely to reflect the lack of a single, objective way to define gamma bands across studies.
In practice, the cited papers use quite different analytical approaches. For example, Fernandez-Ruiz et al. (2017) seem to visually inspect power spectra from ICA-extracted components in multichannel recordings; Lasztoczi & Klausberger define gamma bands based on CSD signals decomposed as amplitude modulations across theta phase (also visual inspection it seems); Scheffer-Teixeira & Tort focus on modulation index (MI) plots; and Lopes-dos-Santos et al. (2018) primarily report peak frequencies of spectrogram IC components rather than frequency bands. Of note, Lopes et al. define fast gamma largely above 100 Hz (tSC5), rather than around 80 Hz as implied in your Figure 1.
At the same time, some features are fairly consistent across studies. For example, several reports place "mid" gamma near the theta peak and associate it with LM. Much of the apparent variability instead seems to arise from loosely defined bands, often based on visual inspection of data processed in different ways, and in some cases it is not even clear how band boundaries are determined. I agree this is a real problem. However, the figure risks overstating how arbitrarily or strategically band boundaries are chosen in order to make these theories appear less falsifiable. I may be missing something here, and if so I'd welcome clarification from the authors.
Relatedly, when discussing Fernández-Ruiz et al. (2021), the authors note that slow gamma is associated with routing object-related information and present this as a contradiction to earlier theories. However, I could not find any claim in that work that this "slow gamma" refers to the same slow gamma proposed by Colgin and others in the context of CA3-CA1 interactions. Rather, Fernández-Ruiz et al. analyze a rhythm linked to LEC-DG communication. This reflects a lack of clarity in how oscillations are labeled, rather than a genuine contradiction of Colgin's framework.

Major 5. The arguments outlined in figs 2 and 3 are really crucial, but they have to be less superficial. For example, one could argue that because CA3 provides its strongest input to stratum radiatum, and much less so to stratum oriens, LFP components recorded in radiatum would naturally be more coherent with CA3 activity. If CA3 pyramidal cells do oscillate in a "slow gamma" band, the EPSPs they impose in radiatum would be expected to reflect that frequency (as argued in detail in PMC8691951 and PMC4803441), whereas signals recorded in oriens would be less so and more easily contaminated by other inputs. In fact, the sharp-wave component associated with ripples has long been attributed to CA3 input precisely because it has maximum energy within radiatum. More generally, it has been common practice to identify dendritic layers by electrically stimulating specific inputs (e.g., PMC6578273, Fig. 11). If inputs from different circuits weren't at least partially localized in their postsynaptic subdomain targets, such approaches wouldn't be feasible. Thus, it seems plausible that if indeed CA3 projection neuron spikes oscillate at a particular frequency, that frequency would be reflected in the LFP recorded from the CA1 dendritic subdomain they innervate. Further, it is also not entirely clear why the authors are so confident in arguing that 'the assertion that "... a large body of converging evidence supports the existence of
distinct slow gamma oscillations localized to CA1 stratum radiatum" (Fernandez-Ruiz et al.,
2023) is inconsistent with the biophysical reality'. At least a few groups have reported a slow gamma component that appears in radiatum (e.g., Schomburg et al., 2014; Lasztóczi & Klausberger, 2016). While I agree that these studies don't demonstrate a CA3 origin, they do report this laminar pattern empirically. It would be helpful if the authors could offer an alternative explanation for why this gamma band is observed in radiatum. Another group has also characterized the laminar profiles of gamma oscillations (PMC5545523; PMID: 22079925), showing that some gamma bands are anatomically organized along the dendritic axis. How the authors reconcile these findings with their argument is not entirely clear.
Crucially, in line 346 the authors write: "Moreover, optogenetic experiments in cortical circuits demonstrate that gamma oscillations can arise solely from local excitatory/inhibitory dynamics (Lasztoczi & Klausberger, 2014; Sohal et al., 2009), reinforcing that gamma may not represent a globally routed signal." However, Lasztóczi & Klausberger (2014) do not include any optogenetic experiments; their conclusions are based on electrophysiology and laminar CSD analyses. While they argue that fast ("perisomatic") gamma can be generated locally, they explicitly attribute slower gamma components to CA3 and entorhinal inputs, which is more consistent with frequency parcellation framework.
Still on this point, in line 393 the authors write: "Data from Belluscio et al. (2012) demonstrate that when the signal is filtered for either slow (30-50 Hz) or fast (50-90 Hz) gamma, both frequency ranges exhibit complementary source-sink distributions across all hippocampal layers." This is not entirely accurate. In Figure 4C of Belluscio et al, slow gamma is clearly strongest in stratum radiatum, whereas fast gamma is more localized to the pyr layer. However, I agree that what is often referred to as mid gamma shows CSD components across multiple layers, which would indeed contradict a strict localization to SLM.

Major 6. Line 429. The authors state that "theories of neural coordination that rely on spectral parcellation are challenged by a lack of direct empirical support that does not require inverse inference." It would be useful to discuss studies that move beyond purely inverse inference. For example, work showing that inhibition of EC inputs selectively disrupts mid-gamma in CA1 (PMC10894649; PMID: 24768692), as well as studies demonstrating that CA3-CA1 and EC-CA1 coherence is preferentially biased toward slow and mid-gamma bands, respectively (Colgin et al., 2009; PMID: 24023818).

Major 7. The methodological concern raised in Figure 4 is extremely interesting and deserves further development. As written, the argument risks coming across as "because theta harmonics exist, gamma oscillations cannot be real," which I don't think does justice to the point the authors are trying to make. The case would be much stronger if the authors proposed concrete, falsifiable tests to distinguish genuine gamma oscillations from harmonics. For example, one simple test would be to show that the slow-gamma component carries more energy than the first theta harmonics (16, 24 Hz). If the slow gamma is meaningful as a legit rhythm it should be at least more prominent than these harmonics; if it merely reflects higher-order theta harmonics, this would not be the case. In addition, do the authors predict that theta oscillations in CA3 are more non-sinusoidal, potentially explaining why this region often shows stronger low-frequency gamma components, as reported by PMID: 19934062 and Schomburg et al. (2014)? Similarly, PMC5585287 reports that the dentate gyrus is dominated by what is often labeled "slow gamma"; would this also be expected to correspond to less sinusoidal theta waveforms? Making such predictions explicit would help clarify the authors' position. More generally, it would be useful to complement Fourier-based analyses with approaches that do not rely on spectral decomposition. For instance, are there neurons in CA3 or DG whose ISI distributions or spike autocorrelograms show clear rhythmic structure at slow-gamma timescales? Evidence at the level of spiking would go a long way toward clarifying whether these components reflect genuine oscillatory processes or arise from waveform nonlinearities.
In this context, it would also be relevant to understand how the authors interpret claims that slow gamma power decreases with running speed (Kemere et al., 2013, Fig. 3c), while theta nonlinearity is known to increase with speed (e.g., PMID: 27076421). Relatedly, what is their interpretation of reports of slow gamma activity in conditions where theta is weak or absent (e.g., PMID: 22920260)?

Major 8. Below, I point out a few places where the citations do not quite line up with the claims being made, and where closer attention would strengthen the manuscript.

1. In line 60, the manuscript states: "The gamma band has often been further subdivided into slow (30-50 Hz) and fast (60-100 Hz) frequencies as well as slow-, mid-, and fast-variants (Belluscio et al., 2012; Lopes-dos-Santos et al., 2018)". However, the two studies cited here both divide hippocampal gamma into slow, mid, and fast components, with fast gamma extending largely above 100 Hz. As written, it is therefore unclear which literature the authors are referring to when they introduce the slow versus fast gamma dichotomy in the middle of the sentence. It could be Colgin et al. (2009), which claims "a slow gamma range (∼25-50 Hz) and a fast gamma range (∼65-140 Hz)". More generally, given the use of the term "often," I would expect this statement to be supported by citations from multiple laboratories or by a review. Clarifying the intended distinction and strengthening the citation base would help avoid confusion at this early stage of the manuscript.

2. Line 62. The sentence reads: "Some propose that slow gamma coordinates memory recall and hippocampal-cortical interactions, while fast gamma supports sensory processing and memory encoding (Bieri et al., 2014; Colgin et al., 2009; Wang et al., 2025)." This appears to be an unintended misstatement. The cited studies do not claim that slow gamma coordinates hippocampal-cortical interactions; rather, they relate slow gamma to CA3->CA1 interactions. Unless the authors are referring to the "slow gamma" band described by Fernández-Ruiz et al. (2021), which is presumably related to LEC->DG interactions. However, this is not the classically defined slow gamma presumably associated with CA3->CA1 interactions and, to my knowledge, has not yet been replicated. I suspect the authors intended to attribute hippocampal-cortical interactions to fast gamma instead, which would be more consistent with the cited literature.

3. Paragraph starting at line 152. In this paragraph, the authors list three limitations of the BBS theory. The first two points are well taken: (1) gamma activity in macaque V1 is strongly dependent on stimulus properties (as also shown in PMC3001273), and (2) concerns about generalizability. The third point, however, concerning spike-leakage artifacts, seems conceptually misplaced in this list. Jia et al. (2013) (as well as PMC3075230) show that high-frequency LFP components are often heavily contaminated by broadband, spike-related signals. This is an important methodological caution, but it is not a direct critique of the BBS theory itself. Rather, it highlights the need to distinguish genuine narrow-band gamma oscillations from broadband high-frequency power when interpreting LFP data, and therefore challenges how gamma is measured rather than the core theoretical claim that synchrony could support binding. More broadly, several substantive criticisms of BBS are not discussed here. For example, much of the foundational BBS literature was conducted in anesthetized animals using highly artificial stimuli, such as drifting gratings optimized for the recorded neurons, and gamma synchrony is often weak or absent under more naturalistic conditions. These limitations seem more directly relevant for assessing the explanatory scope of BBS than concerns about spike-leakage artifacts per se.

4. In the Spectral Multiplexing model, I was extremely confused. Consider the sentence: "This organizes the temporal windows within which faster oscillations (e.g., gamma, 30-100 Hz) can flexibly encode stimulus-specific information, effectively multiplexing signals within the same circuit to support cognitive functions (Bieri et al., 2014; Colgin et al., 2009; Lisman & Jensen, 2013)." None of the cited papers mention 'multiplexing' at all. In Colgin et al. (2009), "routing" refers to frequency-dependent biasing inputs to CA1, typically alternating across theta cycles (they claim a negative correlation). They claim this reflects a form of state-dependent input selection rather than multiplexing or the simultaneous routing of multiple independent information streams that require explicit demultiplexing mechanisms at the receiver (as in frequency-division coding schemes).
Akam & Kullmann (2014), which is cited immediately prior, is an influential review that does discuss oscillatory multiplexing. However, in that context they do not cite Colgin's or Jensen's papers; instead, they refer primarily to their own model (PMC3125699), which is widely known as the multiplexing model. Importantly, that framework does not rely on cross-frequency comodulation, making the remainder of the paragraph read awkwardly. My impression is that the authors are labeling Colgin-style frequency-dependent routing as multiplexing, but doing so creates confusion, since a distinct theory explicitly referred to as multiplexing exists and is cited in the same paragraph. Moreover, the authors write: "A key criticism of spectral multiplexing theories is that they risk being descriptively appealing but mechanistically underspecified." However, PMC3125699 explicitly presents a model showing how such multiplexing could, in principle, be implemented. The authors are of course welcome to critique this model or to argue that it lacks biological plausibility, but this would need to be done explicitly rather than in a vague or generic manner.

5. The sentence "Kemere et al. (2013) found that both slow and fast gamma power increased during memory encoding" is not precise. Kemere et al. show that power in both gamma bands is elevated during exploration of novel environments relative to familiar ones, which is not equivalent to demonstrating a direct increase during memory encoding. That said, the broader point the authors are making remains valid, but the support from this study is more indirect than the current wording implies.

6. Line 296. "Multiple influential studies propose that CA3 and MEC generate distinct gamma frequencies (Colgin et al., 2009; Fernández-Ruiz et al., 2017; Schomburg et al., 2014; Zheng et al., 2016), yet none demonstrate that these upstream cells intrinsically oscillate at the attributed frequencies." The first work reporting slow-frequency gamma in CA3 (they call it 'low' gamma) is not acknowledged (PMC2791641). That said, I agree with the authors' main point: these studies rely largely on LFP analyses and do not show that spiking in upstream populations intrinsically expresses rhythms at those frequencies.

7. Line 373. The sentence "Oriens-lacunosum moleculare (O-LM) cells … are essential for theta-gamma coupling (Tort et al., 2007)." cites a purely computational (modeling) study as if it provided experimental evidence.


Minor comments:

Minor 1. In the CTC paragraph the authors overlook several important criticisms regarding this hypothesis. They do mention the evidence is correlational but they don't spell out. In a few sentences they could mention that much work has shown that coherence is very likely a consequence of communication rather than the cause (explicitly studied here PMC8691951).

Minor 2. InSection 4.3, the authors should highlight studies that have shown experimentally that downstream neurons integrate inputs over fairly long temporal windows, challenging the realistic function of spike synchrony (PMC3890892).

1st Author Response Letter

**Response to comments from Editors and Reviewers:**

**Comments from Reviewer 1**:

Dear Editors and Reviewers,

We are pleased to submit the revised version of our manuscript. We would like to express our sincere gratitude for the exceptionally thorough and constructive reviews we received. The reviewers' insights have substantially strengthened the manuscript, and we found their feedback both intellectually stimulating and genuinely helpful in clarifying our arguments and expanding the scope of our work. We have carefully considered each suggestion and recommendation, and made every effort to meet or exceed the expectations articulated in the reviews.

The revised manuscript represents a substantial reworking of the original submission. We have refined the argumentation, improved clarity, and incorporated additional citations where appropriate. Importantly, the tone of the reviews allowed us to approach this revision not defensively, but constructively. The manuscript is stronger and more disciplined as a result of the reviewers’ engagement, and we are truly appreciative of their role in improving the work.

We have now made the energy cascade / spectral dependence framework more accessible by including figures and making our mechanistic groundings clearer. The manuscript is now more centered on the energy cascade theory rather than primarily critiquing existing models. We incorporated new figures providing critical empirical support, including laminar gamma coherence data, spike timing analysis showing absence of slow gamma in ISI structure, and schematics illustrating the problems that any frequency-specific routing mechanism must solve. Responding to requests for more explicit mechanistic claims and testability, we added a new subsection providing specific, falsifiable predictions of the energy cascade framework, each with explicit empirical tests. We added a major new section extending the framework beyond the hippocampus to neocortex, articulating conserved biophysical constraints and expanding the manuscript’s scope conceptually and empirically. In summary, the revision includes substantial expansions to existing sections and a more systematic articulation of our theoretical framework.

We are deeply appreciative of the time and expertise the reviewers devoted to our manuscript as their constructive engagement has made this a significantly stronger contribution. We hope that the revisions satisfy all concerns and that the manuscript is now suitable for publication. Thank you again for your thoughtful evaluation and for the opportunity to revise and resubmit.

Sincerely,

The authors

**Reviewer comments:**

**Reviewer 1: The origin and function of gamma oscillations remain a major source of speculation in systems neuroscience, with theories ranging widely in both mechanism and functional attribution. This review takes an important step back to reassess these ideas by explicitly asking where gamma may or may not come from, and what it may or may not do. The authors make a clear and forceful argument in favor of a spectral dependence / energy cascade framework, and systematically lay out biophysical and conceptual challenges to models that rely on anatomical and frequency-specific parcellation. This is a contentious position in the field, but an important one to articulate clearly and rigorously. Overall, I find the central thesis compelling and timely, and I support publication of this manuscript. That said, the current presentation sometimes attempts to span too many conceptual levels at once, which can obscure the core message. With improved focus and clearer schematic communication of the proposed model, this review could have substantial impact.**

We sincerely thank the reviewer for their encouraging assessment of our manuscript and for recognizing the timeliness and importance of articulating the 'Energy Cascade' framework. We are particularly grateful for the reviewer’s support of the central thesis and their constructive feedback regarding the manuscript’s organization and visual communication.

We agree that the initial submission attempted to span too many conceptual levels, potentially diluting the core message. In response to the reviewer’s guidance, we have significantly revised the manuscript to improve focus and accessibility:

1. Refined Scope: As suggested, we now explicitly foreground the hippocampus as the primary exemplar system. We have restructured the text to first build the Energy Cascade model using the well-characterized lamination and theta rhythm of the hippocampus, before discussing its potential extension to the neocortex in a dedicated section.

2. Visual Clarity: We have developed new schematics (detailed in response to Major Comment 2) to clearly illustrate the biophysical transition from 'macro scale' theta energy to 'micro-scale' gamma dissipation, moving away from abstract metaphors toward concrete neural mechanisms.

3. Empirical Grounding: To address the need for rigor, we have incorporated prior quantitative analyses (including log-log power scaling and optogenetic falsification tests; New Figure 9) to substantiate the energy cascade model with empirical data rather than purely theoretical arguments.

We believe these revisions have sharpened the manuscript’s focus and significantly enhanced the communication of the proposed model.

**Major comments 1.**

**A recurring issue throughout the manuscript is a tension between broad, cortex-wide claims about neural coordination and a much more detailed, mechanistically grounded discussion centered on the hippocampus. While the hippocampus is clearly used as an exemplar system, this distinction is not always made explicit, and at times the manuscript appears to oscillate between general principles and region-specific arguments without fully reconciling the two.**

**In its current form, this can feel conceptually muddled. Different mechanisms may plausibly dominate in different brain regions, and the hippocampus (given its strong lamination, prominent theta rhythm, and well-characterized circuitry) may be particularly well-suited to an energy cascade framework. I would encourage the authors to more explicitly foreground the hippocampus as the primary focus of the review, and then include a dedicated section toward the end discussing how similar principles might extend to neocortex, along with open questions and potential caveats.**

We thank the reviewer for this important comment. We have revised the manuscript to explicitly foreground the hippocampus as the primary exemplar system for evaluating biophysical constraints on spectral organization, motivated by its laminar architecture, well-characterized circuitry, and prominent rhythms. To address the reviewer’s concern about generalization, we have added a new Section 5 (“Extension Beyond Hippocampus—Constraints, Predictions, and Open Questions”), which discusses how the same biophysical constraints (energy dissipation, inhibition, and fractality) apply to the neocortex, arguing that differences in spectral expression likely reflect differences in anatomical geometry rather than fundamental operating principles. We believe these revisions clarify the scope of the manuscript and resolve the tension noted by the reviewer.

**2. At present, several figures are more effective at critiquing existing models than at positively conveying the authors' own framework. If the energy cascade / spectral dependence model is the key take-home message, it should be made more visually prominent.**

**I strongly encourage the authors to revise or add schematics that more explicitly illustrate:**

*** How energy flows across scales and frequencies**

*** How gamma emerges as a local, state-dependent consequence**

*** How this framework differs qualitatively from parcellation-based views Clearer visual intuition would significantly improve the accessibility and impact of the manuscript.**

**For example, for the graphical abstract, Is the orange 'Y' supposed to be gamma? Personally, I find the schematic on the right describing the energy cascade quite hard to understand. Also, the left schematic is very specific for hippocampal pyramidal cells, while the right on seems to be generic across regions. It might be worthwhile to contrast the two within the same network (e.g., hippocampus)**

We thank the reviewer for this constructive suggestion and agree that the energy cascade / spectral dependence framework should be made more visually prominent and intuitively accessible.

In response, we have completely redesigned the graphical abstract and added a new schematic figure (Figure 1) that places the energy cascade framework at the center of the manuscript, rather than primarily critiquing existing models. The revised graphical abstract now focuses exclusively on the cascade logic itself.

Specifically, the new schematics:

• Illustrate energy flow across scales and frequencies. Excitation is shown entering at large, slow recurrent loops and cascading through progressively smaller, faster circuit motifs, terminating at dissipation. This process is represented as continuous scale-to-scale transfer reflected in the spectral slope, rather than as discrete frequency bands.

• Clarify gamma as a local, state-dependent consequence. Gamma-range activity is depicted as emerging from local recurrent circuitry embedded within larger scale dynamics, not as an independent oscillator or pathway-specific communication channel.

• Ground the cascade within the specific hippocampal circuit. The cascade framework is now explicitly mapped onto the hippocampal network, addressing the reviewer’s concern that earlier schematics used generic depictions. Ambiguous elements in the original graphical abstract (e.g., the orange “Y”) have been removed, and the visual language has been simplified to emphasize scale dependence.

Finally, we revised the accompanying figure captions and the Introduction to foreground the biophysical constraints and organizing principles of the cascade framework. To avoid clutter, the conceptual schematics are presented separately from the quantitative tests; the empirical contrast between cascade and routing predictions is provided across multiple new figures.

**3. Lines 357-359 make a central argument about low coherence between hippocampal layers. Given how important this point is for undermining spectral routing models, it would strengthen the manuscript to either show this directly or reproduce representative data from prior studies. Even a brief figure panel or citation to an explicit coherence analysis would help anchor this claim empirically.**

We thank the reviewer for this suggestion and agree that this claim warrants explicit empirical anchoring. To address this point, we have revised the text and added a new figure (Figure 5) that directly illustrates laminar gamma-band coherence in the hippocampus. Specifically, we now include gamma-band (30–90 Hz) coherence maps from Berényi et al. (2014), who used high-density laminar recordings to compute coherence between reference sites and all other locations spanning hippocampal layers during exploration.

As shown in Figure 5, gamma coherence is consistently high within individual laminae but drops sharply across laminar boundaries, including between stratum radiatum, stratum lacunosum-moleculare, and stratum oriens. This pattern demonstrates that gamma synchrony is spatially confined and does not exhibit the cross-layer coherence that would be required for frequency-specific routing of CA3 versus entorhinal inputs. We have also tightened the surrounding text to explicitly state the falsifiable prediction of spectral routing models—namely, that frequency-matched cross-layer coherence should be present if gamma bands function as communication channels—and to clarify that this prediction is not supported by the data.

Importantly, this conclusion is not based on a single dataset. We now explicitly reference convergent laminar and coherence analyses from multiple studies (e.g., Berényi et al., 2014; Zhou et al., 2019; 2022; Zhao et al., 2025), which collectively show that while theta coherence remains robust across hippocampal layers, gamma coherence is low, spatially confined, and highly phase-dispersed across laminar boundaries. Together, the revised text and added figure empirically anchor the claim that inter-laminar gamma coherence in hippocampus is low and reinforce our conclusion that gamma oscillations are better understood as locally generated, layer-constrained dynamics rather than as globally routed frequency channels.

**4. Does the energy cascade framework posit that, given variables such as running speed, theta power, or theta waveform shape, the emergent gamma power and frequency should be predictable? If so, it could be interesting to demonstrate this explicitly (e.g., via regression or variance-explained analyses). Alternatively, if gamma cannot be fully predicted from these factors, it would be useful to clarify what the authors view as the dominant sources of variability. Making this explicit would help readers understand the limits and testable predictions of the framework.**

To address this point explicitly, we have added a new regression analysis (Figure 9c) and revised the text to clarify the **scope and limits of predictability** implied by the energy cascade framework.

The framework predicts that gamma-band activity should be **statistically coupled to the magnitude and structure of low-frequency energetic drive**, but not that gamma power or frequency should be fully determined by a small set of macroscopic variables.

Consistent with this prediction, we now show that gamma power covaries with theta band energy in two complementary contexts. First, in rat CA1 during natural behavior (Sheremet et al., 2019), theta power significantly predicts gamma-band amplitude (Figure 5c), explaining a modest but reliable fraction of the variance (up to ~25% in stratum radiatum), consistent with gamma reflecting local dissipation driven by slower network dynamics. This relationship demonstrates **scaling predictability**, rather than deterministic control.

Second, in mouse CA1 during optogenetic perturbation (Zhao et al., 2026), alterations in theta waveform structure—quantified via harmonic power—account for a substantially larger fraction of the variance in gamma power (~50%). We interpret this increase not as evidence of precise decoding, but as a consequence of experimentally imposing large changes in energetic input, which amplifies the coupling predicted by the cascade framework.

Importantly, the framework does not predict that gamma activity should be fully specified by running speed, theta power, or waveform shape alone. The remaining variance reflects intrinsic properties of a driven, dissipative neural system, including stochastic synaptic dynamics, laminar-specific circuit architecture, and state-dependent modulation of excitation–inhibition balance. We have clarified this point in the revised text to emphasize that the energy cascade predicts **covariation and constrained scaling across frequencies**, rather than complete predictability of high-frequency features.

**5. Lines 590-592 state that "theta may provide a global temporal framework." Conceptually, this seems similar to roles that have previously been attributed to gamma in the literature. It would be helpful if the authors could more explicitly clarify how their proposal differs in substance, rather than simply shifting the coordinating role from gamma to theta.**

**This clarification would help avoid the impression that the same functional logic is being reassigned to a different frequency band.**

We thank the reviewer for raising this important conceptual concern. We agree that, if framed purely in terms of frequency labels, assigning a “coordinating” role to theta could appear similar to roles previously attributed to gamma. Our proposal, however, differs in substance because it is grounded in **physical scale and circuit hierarchy**, not a reassignment of functional labels.

In the Energy Cascade framework, theta and gamma are not treated as equivalent oscillators capable of exchanging roles. Instead, they occupy **distinct positions in a hierarchical dynamical process**. Theta reflects large-scale, slow dynamics generated by extended anatomical loops and conduction delays spanning millimeters to centimeters. Because of this physical scale, theta is capable of organizing activity across distributed regions by providing a permissive temporal window that tolerates transmission delays and supports sequential integration.

By contrast, gamma reflects **local, meso-scale dynamics** generated by excitation inhibition microcircuits. In this view, gamma is not a global coordinating signal but a **local, state-dependent dissipative feature** whose magnitude and structure depend on the underlying low-frequency drive. Assigning global coordination to gamma therefore imposes biophysical requirements (precise millisecond simultaneity across long distances) that are difficult to reconcile with known conduction delays and the typically low interregional gamma coherence.

Thus, we are not shifting the same coordinating logic from gamma to theta. Rather, we redefine coordination itself: from millisecond-scale simultaneity (as assumed by gamma centric binding models) to **ordered temporal organization at a slower scale**, consistent with phase precession and compression phenomena. We have clarified this distinction explicitly in the revised manuscript to avoid the impression of a semantic relabeling.

Section 4.2: “Rather than assigning specific cognitive functions to theta, it is more accurate to view it as a biophysical scaffold for transmembrane currents that organize network dynamics across space and time (Buzsaki, 2002). Theta does not encode task variables; instead, it reflects the global state of network engagement.”

**6. Given the much lower and less consistent theta power in humans and non human primates, it would be interesting to see whether the framework makes specific predictions about how gamma should differ across species. Showing that gamma is altered in predictable ways based on differences in theta structure could substantially strengthen the argument.**

We thank the reviewer for this important comparative question. The Energy Cascade framework does make specific, testable predictions about cross-species differences, and this comment provides an opportunity to clarify a key point: *the organizing rhythm is not defined by a fixed frequency band (e.g., rodent 8 Hz theta), but by its role as a large-scale carrier constrained by anatomical geometry (we have amended paragraphs/included new text in section 4.4.* ***Testable Predictions and Falsifiability of the Energy Cascade Framework*** *to address this").*

**7. What determines the specificity of rate gain modulation in the BBRE framework? Clarifying how selective rate enhancement arises (particularly in the absence of precise synchrony) would help readers understand how BBRE avoids becoming overly global or nonspecific.**

We thank the reviewer for raising this point and agree that clarifying the source of specificity in the BBRE framework is important. In BBRE, rate enhancement is not global but highly conditional. Specificity arises from the intersection of bottom-up feature activation and top-down feedback signals: only neurons that are already active due to feedforward input receive gain modulation from feedback, preventing nonspecific excitation.

In addition, BBRE relies on incremental grouping mechanisms mediated by horizontal and feedback connections, such that enhancement propagates selectively along connected elements of an object (e.g., during curve tracing) and terminates at perceptual boundaries. Local inhibitory circuitry further sharpens specificity, with disinhibitory pathways (e.g., VIP interneurons) enhancing figure representations while other inhibitory populations suppress background activity. Finally, BBRE posits a degree of temporal seriality, whereby objects are grouped sequentially rather than simultaneously, preventing uncontrolled binding across multiple objects.

We have revised the manuscript to briefly summarize these mechanisms in the new section 5.1:

“BBRE does not posit global or indiscriminate gain increases. Rate enhancement is conditional on existing feedforward activation and is selectively gated by top-down feedback and local disinhibitory circuits, including VIP-mediated motifs. As a result, increased firing remains confined to neurons already participating in task-relevant representations, supporting object- and feature-specific binding without reliance on millisecond-scale synchrony (Roelfsema, 2023). This conditional gating avoids the loss of specificity that would accompany uniform gain modulation.”

**8. Given the manuscript's emphasis on falsifiability as a weakness of spectral parcellation-based theories, it would strengthen the framework to more explicitly state what empirical observations would challenge or falsify an energy cascade account. Clarifying these limits would help sharpen the proposal and guide future experimental tests.**

We thank the reviewer for raising this important point. We fully agree that for the Energy Cascade framework to be scientifically meaningful, it must make explicit, falsifiable predictions rather than serving as a descriptive metaphor.

In response, we have added a dedicated subsection to the manuscript (new Section 4.4, “Testable Predictions and Falsifiability of the Energy Cascade Framework”) that explicitly delineates empirical observations that would challenge or falsify the model.

Briefly, the Energy Cascade framework would be falsified by observations that violate its core assumption of hierarchical energetic dependence across scales. These include: (1) sustained, high-amplitude gamma oscillations emerging independently of low-frequency drive; (2) experimental manipulations in which gamma power increases or remains stable while low-frequency power and total network energy are reduced, violating predicted scaling relationships; and (3) evidence that local gamma-frequency perturbations can entrain or organize global low-frequency rhythms, reversing the proposed direction of influence.

We further note that these falsification criteria are not merely hypothetical. For example, systematic energy withdrawal experiments (Zhou et al., 2021) demonstrate hierarchical spectral degradation in which high-frequency components collapse before low frequency rhythms, consistent with cascade predictions and inconsistent with independent-oscillator accounts. Similarly, circuit perturbations that selectively target proposed “gamma generators” fail to abolish specific gamma bands while preserving others (Zhao et al., 2026), directly contradicting discrete parcellation models. We have revised the manuscript to make these limits and predictions explicit, thereby sharpening the empirical testability of the framework and clarifying how it can be evaluated against competing theories.

**Reviewer 2: This manuscript presents an ambitious theoretical review that critically evaluates gamma frequency-specific models of neural communication and promotes an energy-cascade interpretation, in which slow, large-scale rhythms organize neural activity by cascading energy into faster, local dynamics rather than functioning as dedicated communication channels. The authors should be applauded for this effort. Constructive criticism of influential theories is crucial for progress in the field, particularly when those theories have become deeply embedded in how data are interpreted**.

**I believe this manuscript has the potential to become an important reference on the topic and to stimulate constructive debate. However, in its current form, some sections feel overly superficial, while others rely on citations in a way that comes across as somewhat cursory. Some key concepts are introduced in a rather hand wavy manner, which may be challenging for readers who are not already deeply familiar with the literature. While I don't believe this is the authors' intention, parts of the discussion risk giving the impression that alternative frameworks are addressed in a somewhat straw-man fashion. Below, I go into more detail on these points.**

First, we wish to express our genuine gratitude to Reviewer 2 for what we consider an exceptionally rigorous, insightful, and constructive review. It is rare to receive feedback that engages so deeply with the theoretical and biophysical substance of a manuscript. The reviewer’s precise critiques regarding falsifiability, their encyclopedic command of the literature, and their insistence on mechanistic concreteness have forced us to sharpen our arguments substantially.

We are especially grateful for the reviewer’s efforts to ensure that alternative frameworks were treated precisely and fairly. While our conclusions remain unchanged, the manuscript is materially stronger as a result of this exchange. We consider this review a model of constructive scientific critique and sincerely appreciate the time and expertise that went into it.

In response, we have undertaken a careful revision to ensure that our arguments are both mechanistically grounded and accurately framed. Specifically, we have:

1. **Refined theoretical distinctions:** We now explicitly distinguish between frequency-based routing (e.g., Colgin et al., 2009) and multiplexing frameworks (e.g., Akam & Kullmann, 2014), addressing the reviewer’s concern that these positions were previously treated too generically.

2. **Operationalized the energy cascade framework**: To reduce abstraction and improve accessibility, we have grounded the cascade model in concrete biophysical terms and incorporated quantitative analyses from prior work (Sheremet et al., 2019) demonstrating systematic scaling between low- and high frequency activity.

3. **Clarified the scope of our critique:** We emphasize that our argument is not that alternative frameworks ignore microcircuit mechanisms, but that they often assume functional independence between oscillatory components that may be inconsistent with known energetic and circuit constraints.

To make these changes concrete, we now treat routing-based (CTC) and multiplexing models in distinct, dedicated sections (Sections 3.2 and 3.4), explicitly outlining each framework’s strongest mechanistic commitments before identifying where independence assumptions enter. We have also operationalized the energy cascade framework by explicitly defining “energy” in biophysical terms (synaptic transmembrane currents) and by incorporating quantitative spectral-slope analyses from Sheremet et al. (2019), which demonstrate systematic, state-dependent scaling between low- and high frequency activity. These additions replace abstract description with explicit mechanisms and measurable quantities, directly addressing the concern about hand waving and oversimplification.

We believe these revisions directly address the reviewer’s concerns by strengthening the mechanistic grounding of the manuscript and clarifying the precise points of theoretical disagreement.

**Major comments**

**Major 1. Section '4.1. The Energy Cascade Model for Neural Coordination." is arguably the most important part of the manuscript, and I'd strongly encourage the authors to invest more effort in presenting this framework in a concrete and accessible way. As it stands, the discussion feels somewhat abstract and is not clearly aimed at a broad neuroscience audience, which may limit its impact. For example, sentences such as "draws from concepts in physics, particularly Kolmogorov's theory of turbulence (1941), suggesting that oscillations reflect a hierarchical process where activity propagates from larger, slower oscillations to smaller, faster ones in a cascade-like manner" are conceptually appealing, but remain difficult to translate into neural terms. Readers who are not already familiar with turbulence theory are left without a clear sense of what this cascade looks like in terms of neuronal activity and circuit interactions. It'd greatly strengthen the manuscript if the authors could provide a simple, tangible model (perhaps a schematic or worked example) illustrating how this cascade operates in a neural circuit.**

We thank the reviewer for this important comment and agree that the original presentation relied too heavily on analogy without sufficiently explicit translation into neural mechanisms. In the revised manuscript, we have substantially rewritten Section 4.1 to define the energy cascade directly in biophysical terms.

Specifically, we now clarify that “energy” refers to the magnitude and timing of synaptic transmembrane currents, both excitatory and inhibitory, that generate extracellular field potentials (Buzsáki et al., 2012). In this framework, the cascade does not describe the propagation of a signal across frequencies, but a directional process by which slow, large-scale rhythms such as theta modulate network excitability and synaptic current flow, thereby conditionally recruiting fast local excitatory–inhibitory feedback loops.

To make this mechanism tangible, we have added a new schematic figure (**Figure 1**) illustrating the specific neural instantiation of the cascade.

The figure visualizes the hierarchy explicitly:

1. **Macroscale (Energy Injection):** Large reentrant loops with long conduction delays generate the slow organizing rhythm (e.g., theta).

2. **Mesoscale (Redistribution):** This slow drive recruits local, faster excitatory inhibitory microcircuits (i.e., gamma) within the cytoarchitectural layers.

3. **Microscale (Dissipation):** The cascade terminates at the synaptic level, where energy is dissipated as transmembrane currents and action potentials.

Furthermore, we have revised the text to 'translate' the physics analogies into concrete neurophysiology. For example, rather than relying solely on the Richardson-Kolmogorov turbulence metaphor, we now explain that '**Forcing**' corresponds to large-scale synaptic drive (e.g., theta modulation), **'Inertial Range'** corresponds to the redistribution of that drive through nested local circuits (gamma), **'Dissipation'** corresponds to the biophysical limits of the neuron (leak currents, synaptic failure, and refractory periods) that prevent infinite signal propagation.

These revisions ensure that the cascade framework is accessible to a broad neuroscience audience and grounded in familiar circuit elements rather than abstract fluid dynamics.

**Major 2. The authors frame the frequency-parcellation framework as being difficult to falsify, which is an important and well-taken point. However, this argument would benefit from being developed more explicitly. The best way to strengthen it would be to propose concrete, falsifiable tests of the theory. For example, the authors note that interareal coherence is often weak, largely observed at the LFP level, and potentially artifactual. Recent work showing that downstream pyramidal cells don't reliably follow gamma-paced inputs (PMC7616856) already challenges a strong interpretation of gamma-based "routing." If slow and mid gamma oscillations do reflect CA3->CA1 and EC->CA1 interactions, respectively, one would predict that spike-to-spike raw cross correlations between these regions should exhibit clear components at the corresponding frequencies. Similarly, disrupting one pathway should lead to a selective reduction of its associated gamma band in the downstream circuit. The authors may have additional ideas for such tangible tests. Importantly, the same kind of falsifiable predictions should also be articulated for the proposed energy cascade framework**.

We thank the reviewer for this thoughtful and constructive comment. We agree that the critique of frequency-parcellation frameworks is strongest when grounded in explicit, falsifiable predictions, and we have revised the manuscript accordingly to clarify both (i) how parcellation models can be empirically tested and (ii) how the proposed energy cascade framework makes distinct, testable predictions (New section 4.4).

First, the reviewer correctly notes that if discrete gamma bands reflect selective routing of information between pathways (e.g., CA3→CA1 versus EC→CA1), then several concrete outcomes should follow. Specifically, one would predict (a) robust spike-to spike or spike-to-field correlations at the corresponding gamma frequencies between upstream and downstream pyramidal populations, and (b) selective attenuation of the associated gamma band following disruption of a specific pathway.

Recent work directly challenges these predictions. As the reviewer noted, Spyropoulos et al. 2024 (now cited in revised manuscript) demonstrate that although inter-areal LFP LFP gamma coherence can be robust, downstream excitatory neurons do not reliably phase-lock to upstream gamma rhythms. Instead, gamma-phase locking is confined primarily to fast-spiking interneurons in feedforward input layers, while pyramidal neurons (those required for information transmission) show little or no gamma-locked spiking (Schomburg et al., 2014). Moreover, decoding analyses reveal that firing rates, rather than gamma phase relationships, carry substantially more information about behavioral state. These findings undermine a strong interpretation of gamma-based routing that relies on coherent spike transmission across areas.

Second, with respect to hippocampal “slow” and “fast” gamma distinctions, we have revised the text to clarify that our critique is not aimed at any single anatomical attribution (e.g., CA3 versus LEC), but at the shared assumption that separable oscillators exist in narrowly defined gamma sub-bands. Whether slow gamma is attributed to CA3 input (Colgin et al., 2009) or to LEC input (Fernández-Ruiz et al., 2021), both accounts require that a distinct 30–50 Hz oscillator can be isolated from the underlying theta dynamics. We argue that this assumption is problematic. In particular, non-sinusoidal theta waveforms naturally generate harmonic structure in the gamma range, and commonly used short-window spectral analyses cannot reliably distinguish such harmonics from independent oscillations. As detailed in our response to Major 7, we clarify that violations of the time–frequency uncertainty can lead to spurious dissociation of theta harmonics, which may then be misinterpreted as separate gamma bands.

Finally, we now articulate explicit falsifiable predictions for both frameworks. For frequency-parcellation models, selective pathway disruption should only abolish the corresponding gamma band in downstream pyramidal spiking and spike–spike correlations. By contrast, in the energy-cascade framework, gamma power should scale predictably with low-frequency drive and excitability, persist primarily in inhibitory circuitry, and degrade globally when cross-scale flow is disrupted rather than disappearing selectively. These predictions are now summarized in a new subsection on testable consequences.

Together, these revisions clarify that our critique is not philosophical but empirical: the parcellation framework makes strong predictions about spike-level transmission that are increasingly difficult to reconcile with laminar, cell-type–resolved data, whereas the energy-cascade framework remains consistent with both existing results and explicit falsification criteria.

**Major 3. In the section "Historical perspectives on the role of gamma rhythms in cognition," the authors raise an important concern about the risks of disconnecting oscillations measured at the electrode from the microcircuits that generate them. In the final sentence of that section, they write: "Foundational notions of gamma as a signature of E/I volleys in local networks has been overshadowed by more contemporary ideas regarding the role that gamma may play in coordinating disparate brain regions or routing the transfer of neural activity between distinct anatomical pathways." While this is an important point, it is not developed in sufficient detail, and I was left unsure about what, exactly, the authors intend by this contrast.**

**If proponents of frequency parcellation argue that distinct anatomical pathways give rise to different gamma frequencies, this would seem, at least in principle (albeit very superficially), to link oscillatory phenomena to underlying microcircuit properties. For example, if one region exhibits resonance properties due to its local E/I dynamics at a given frequency, while another region has a distinct microcircuit with a different resonance frequency, wouldn't the postsynaptic potentials imposed by these two inputs produce different frequency components in the LFP measured at their shared downstream target? In that sense, pathway-specific frequency hypotheses do not obviously abandon microcircuit grounding. My impression is that the authors do not intend to suggest that frequency-parcellation models ignore microcircuitry altogether, but rather that they are often imprecise or under-specified with respect to how specific circuit mechanisms give rise to specific frequencies. If this is the intended critique, it would benefit from being stated more explicitly. If instead this reflects a misunderstanding on my part, the authors may need to clarify more clearly what point is being made, as the current framing reads as somewhat vague.**

**A recent paper (PMC9069800) shows that suppressing SST interneurons in DG CA3 shifts gamma-band activity in CA1 away from slow gamma, whereas suppressing DG-CA3 PV cells has the opposite effect and enhances what they call "fast gamma" (defined as 50-100Hz, which overlaps more closely with what other groups refer to as mid gamma and often associate with EC inputs). Wouldn't this represent an initial step toward grounding these ideas mechanistically? While the link to CA3 versus EC drive remains interpretational, the results suggest that different interneuron classes contribute differently to distinct gamma bands. This seems like a key piece of work that would be worth discussing in this section of the manuscript. Further, Lasztóczi & Klausberger (PMID: 24607232) have made some effort in characterizing how different interneurons within CA1 relate to different gamma bands.**

**This issue becomes more pronounced in light of Section 4.1 (The Energy Cascade Model for Neural Coordination), where the authors introduce the framework they favor. That section relies on language that is quite abstract for most readers, as mentioned in Major 1. For example, the statement that "In this context, 'energy' refers to the aggregate power of synaptic currents and transmembrane conductance driving oscillatory activity. The result is a directional flow of energy across temporal scales—not simply co-occurring rhythms, but a cascade from a global input (source) to a localized dissipation (sink)" is considerably less concrete than the pathway-based hypotheses it is meant to replace. I'm not arguing that the energy-cascade framework is in less physiologically grounded, but rather that the manuscript does little to explain why it should be considered more directly connected to known circuit mechanisms than "frequency-parcellation" accounts. As a result, the claimed contrast between mechanistic grounding and abstraction remains insufficiently justified and, at times, even contradictory.**

We thank the reviewer for this thoughtful and technically sophisticated comment. We agree that the distinction we intended to draw between foundational microcircuit accounts of gamma and more recent frequency-parcellation frameworks was not articulated with sufficient precision in the original draft. We appreciate the opportunity to clarify this point and to integrate the important interneuron-specific studies highlighted by the reviewer.

**Clarifying the Intended Contrast**

The reviewer is correct that, in principle, frequency-parcellation models can be grounded in microcircuit mechanisms. Indeed, the idea that distinct interneuron populations or pathway-specific inputs might favor different resonance frequencies is not inherently incompatible with circuit physiology. Our critique is not that parcellation frameworks ignore microcircuits altogether.

Rather, our concern is that, in practice, many parcellation models implicitly **reify frequency bands as independent oscillatory entities** (that is, as separable communication channels that can be selectively routed, gated, or multiplexed) without sufficiently constraining these claims by the biophysical limits of synaptic integration, neuronal filtering, and spectral decomposition. We have revised the manuscript to make this distinction explicit.

**Interneuron-Specific Contributions to Gamma**

We appreciate the reviewer’s suggestion to consider PMC9069800 and related work reporting interneuron-class-specific modulation of gamma-band power. We agree these studies reinforce that inhibitory microcircuit properties strongly shape the spectral expression of hippocampal field activity.

However, we do not view these results as grounding frequency-based routing mechanisms for two reasons. First, the operational definition of “slow gamma” in such datasets is not specific to an independent oscillatory generator. Band-limited power in the 30–50 Hz range can be contaminated when quantified during sharp-wave/ripple rich epochs or using short-window time–frequency methods (PMC6310484), where transient waveform structure and event envelopes generate broadband and harmonically related spectral power that can appear as sub-band shifts. Under these conditions, apparent changes in “slow gamma” can reflect changes in event statistics (e.g., SPW/R incidence, overlap, or morphology) and spectral leakage rather than modulation of a separable rhythm.

Second, even when band-limited measures are robust, interneuron perturbations do not isolate oscillatory frequency as a causal variable. PV⁺ and SST⁺ manipulations necessarily co-modulate synaptic drive, excitability, inhibitory kinetics, and population firing statistics, that is, the network state generating the field potential. Frequency changes are therefore most parsimoniously interpreted as state-dependent consequences of altered circuit impedance and decay constants. Given circuit degeneracy, a spectral shift does not uniquely identify a routing mechanism, nor does it establish that frequency functions as an independently selectable communication channel.

For these reasons, we interpret interneuron-class specificity as consistent with an energy-cascade or local-resonance framework in which interneuron kinetics and targeting determine how shared synaptic drive is locally dissipated and expressed in the LFP, rather than as evidence for pathway-specific frequency channels. We have now included a discussion of PMC9069800 alongside Yamamoto et al., 2014 and Liu et al., 2022. While PMC9069800 frames slow and fast gamma as signatures of internal versus external drive, the experiments themselves demonstrate circuit-dependent modulation of spectral structure rather than frequency-selective communication

**Methodological Constraints on Identifying Distinct Gamma Bands**

A central issue underlying this debate—and one that directly bears on the interpretation of the cited interneuron studies—is the **spectral identifiability of putative gamma sub-bands.**

As noted by Schomburg et al. (2014), modulation in the 20–30 Hz and 30–50 Hz ranges may reflect **non-sinusoidal features and harmonics of theta**, rather than independent oscillatory processes. This concern is not merely conceptual but arises from well established limits in time–frequency analysis. Wavelet-based approaches, particularly those optimized for temporal precision, necessarily trade frequency resolution for time resolution (Gabor–Heisenberg limit). Under these conditions, higher-order harmonics and waveform asymmetries of slow rhythms can be mathematically dissociated from their parent oscillation and appear as distinct “bands.”

We now explicitly discuss this issue in the revised manuscript and cite prior methodological work demonstrating that commonly used wavelet and EEMD approaches can produce the appearance of distinct slow-gamma bands even in synthetic signals containing only theta and its harmonics (Zhou et al., 2019, eNeuro). Importantly, this critique applies equally to studies attributing slow gamma to CA3, MEC, or LEC inputs: the anatomical label changes, but the underlying spectral ambiguity remains.

Thus, while interneuron manipulations can shift spectral power between frequency ranges, such shifts do not, by themselves, demonstrate the existence of multiple independent gamma oscillators or routed frequency channels. Instead, they are fully consistent with a model in which **the same broadband synaptic drive is redistributed across temporal scales depending on which inhibitory loops are available to dissipate it**.

**Why the Energy Cascade Framework Is More Mechanistically Constrained**

The energy cascade framework differs from frequency-parcellation accounts not by invoking less physiology, but by invoking **more constraints**. In our revised presentation, “energy” is defined concretely as aggregate synaptic current and conductance entering a local circuit. The cascade refers to the transformation of slow, large-scale excitatory drive into faster inhibitory feedback, with oscillatory frequency emerging as a **consequence** of circuit kinetics rather than a symbolic carrier of information.

This framework makes explicit, falsifiable predictions about cross-scale covariation, continuous frequency shifts with circuit state, and hierarchical degradation under perturbation—predictions that do not follow naturally from independent-channel models. We believe this clarification resolves the apparent contradiction identified by the reviewer and strengthens the mechanistic grounding of the proposed framework.

**Major 4. I did not find the arguments presented in Figure 1 and the associated text particularly convincing. The authors suggest that variability in reported gamma band definitions across studies reflects a tendency for other groups to flexibly adjust frequency boundaries to fit their theoretical interpretations. However, this variability seems more likely to reflect the lack of a single, objective way to define gamma bands across studies.**

**In practice, the cited papers use quite different analytical approaches. For example, Fernandez-Ruiz et al. (2017) seem to visually inspect power spectra from ICA-extracted components in multichannel recordings; Lasztoczi & Klausberger define gamma bands based on CSD signals decomposed as amplitude modulations across theta phase (also visual inspection it seems); Scheffer-Teixeira & Tort focus on modulation index (MI) plots; and Lopes-dos Santos et al. (2018) primarily report peak frequencies of spectrogram IC components rather than frequency bands. Of note, Lopes et al. define fast gamma largely above 100 Hz (tSC5), rather than around 80 Hz as implied in your Figure 1. At the same time, some features are fairly consistent across studies. For example, several reports place "mid" gamma near the theta peak and associate it with LM. Much of the apparent variability instead seems to arise from loosely defined bands, often based on visual inspection of data processed in different ways, and in some cases it is not even clear how band boundaries are determined. I agree this is a real problem. However, the figure risks overstating how arbitrarily or strategically band boundaries are chosen in order to make these theories appear less falsifiable. I may be missing something here, and if so I'd welcome clarification from the authors.**

**Relatedly, when discussing Fernández-Ruiz et al. (2021), the authors note that slow gamma is associated with routing object-related information and present this as a contradiction to earlier theories. However, I could not find any claim in that work that this "slow gamma" refers to the same slow gamma proposed by Colgin and others in the context of CA3-CA1 interactions. Rather, Fernández-Ruiz et al. analyze a rhythm linked to LEC-DG communication. This reflects a lack of clarity in how oscillations are labeled, rather than a genuine contradiction of Colgin's framework.**

We appreciate the reviewer’s point that variability in gamma-band definitions reflects methodological diversity rather than strategic boundary adjustment, and we have revised the text accordingly. We would like to clarify, however, that our primary concern is not variability per se, but the absence of a corresponding mechanistic account.

In much of the frequency-parcellation literature, the identification of “slow,” “mid,” or “fast” gamma rests primarily on spectral decomposition, with limited discussion of how multiple discrete gamma oscillations would be generated and maintained by known hippocampal circuitry. If such bands reflect distinct biological oscillators, then a mechanistic explanation is required: Are they driven by distinct afferent pathways, by separable interneuron networks, or by intrinsic membrane resonances? For example, while Colgin et al. (2009) and Fernández-Ruiz et al. (2021) associate activity in the 20 50 Hz range with different anatomical inputs (CA3 versus LEC), neither study proposes a circuit mechanism that would distinguish these rhythms biophysically. In the absence of such a mechanism, differences in labeling alone do not establish the existence of distinct oscillatory entities, any more than observing theta in multiple regions implies multiple types of theta oscillation.

Similarly, while interneuron-specific manipulations can shift gamma spectral content, existing models do not specify how GABA𝐴-mediated feedback—with relatively constrained synaptic time constants (Wang & Buzsáki, 1996)—would support multiple stable gamma bands rather than continuous frequency shifts. Our critique is therefore not that spectral analyses are inconsistent, but that frequency labels are often treated as explanatory endpoints rather than as observations that themselves require mechanistic grounding.

We have revised the manuscript (section 3.5 *Hippocampal Gamma as an Exemplar of Spectral Parcellation Models*) to make this distinction explicit and to emphasize that the energy cascade framework addresses this gap by linking frequency structure to circuit constraints and continuous state-dependent dynamics, rather than to independent oscillatory channels.

**Major 5. The arguments outlined in figs 2 and 3 are really crucial, but they have to be less superficial. For example, one could argue that because CA3 provides its strongest input to stratum radiatum, and much less so to stratum oriens, LFP components recorded in radiatum would naturally be more coherent with CA3 activity. If CA3 pyramidal cells do oscillate in a "slow gamma" band, the EPSPs they impose in radiatum would be expected to reflect that frequency (as argued in detail in PMC8691951 and PMC4803441), whereas signals recorded in oriens would be less so and more easily contaminated by other inputs. In fact, the sharp-wave component associated with ripples has long been attributed to CA3 input precisely because it has maximum energy within radiatum. More generally, it has been common practice to identify dendritic layers by electrically stimulating specific inputs (e.g., PMC6578273, Fig. 11). If inputs from different circuits weren't at least partially localized in their postsynaptic subdomain targets, such approaches wouldn't be feasible. Thus, it seems plausible that if indeed CA3 projection neuron spikes oscillate at a particular frequency, that frequency would be reflected in the LFP recorded from the CA1 dendritic subdomain they innervate. Further, it is also not entirely clear why the authors are so confident in arguing that 'the assertion that "... a large body of converging evidence supports the existence of distinct slow gamma oscillations localized to CA1 stratum radiatum" (Fernandez Ruiz et al., 2023) is inconsistent with the biophysical reality'. At least a few groups have reported a slow gamma component that appears in radiatum (e.g., Schomburg et al., 2014; Lasztóczi & Klausberger, 2016). While I agree that these studies don't demonstrate a CA3 origin, they do report this laminar pattern empirically. It would be helpful if the authors could offer an alternative explanation for why this gamma band is observed in radiatum. Another group has also characterized the laminar profiles of gamma oscillations (PMC5545523; PMID: 22079925), showing that some gamma bands are anatomically organized along the dendritic axis. How the authors reconcile these findings with their argument is not entirely clear. Crucially, in line 346 the authors write: "Moreover, optogenetic experiments in cortical circuits demonstrate that gamma oscillations can arise solely from local excitatory/inhibitory dynamics (Lasztoczi & Klausberger, 2014; Sohal et al., 2009), reinforcing that gamma may not represent a globally routed signal." However, Lasztóczi & Klausberger (2014) do not include any optogenetic experiments; their conclusions are based on electrophysiology and laminar CSD analyses. While they argue that fast ("perisomatic") gamma can be generated locally, they explicitly attribute slower gamma components to CA3 and entorhinal inputs, which is more consistent with frequency parcellation framework. Still on this point, in line 393 the authors write: "Data from Belluscio et al. (2012) demonstrate that when the signal is filtered for either slow (30-50 Hz) or fast (50 90 Hz) gamma, both frequency ranges exhibit complementary source-sink distributions across all hippocampal layers." This is not entirely accurate. In Figure 4C of Belluscio et al, slow gamma is clearly strongest in stratum radiatum, whereas fast gamma is more localized to the pyr layer. However, I agree that what is often referred to as mid gamma shows CSD components across multiple layers, which would indeed contradict a strict localization to SLM.**

We thank the reviewer for this rigorous biophysical critique and for the precise corrections regarding the cited literature. We agree that laminar inputs dictate where synaptic currents enter the circuit, and we apologize for the inaccuracy regarding the methods in Lasztóczi & Klausberger (2014), which we have corrected in the revised text.

However, on the central issue of interpreting laminar CSD profiles, we maintain that anatomical localization of a current sink does not confirm the existence of a frequency specific upstream oscillator. We have revised Section 3.5 to address the reviewer's points as follows:

1. **Anatomy constraints location, not frequency** We agree entirely that because CA3 projects to stratum radiatum, EPSPs driven by CA3 spikes will generate sinks in that layer. However, this only tells us where the current flows, not why it oscillates at a specific frequency. A sink in radiatum at 40 Hz could arise from:

• A true 40 Hz oscillator in CA3 (the Parcellation view).

• A broadband/stochastic CA3 drive that is filtered by the local radiatum impedance at 40 Hz (the Cascade view).

• A harmonic of a non-sinusoidal theta waveform that simply has sharp slopes in that layer. Thus, observing a sink in radiatum is necessary but not sufficient to claim CA3 transmits a 'slow gamma' code.

2. **Reference-Dependent Amplification in CSD Analyses (Addressing the 'Converging Evidence')** The reviewer asks why we question the 'converging evidence' for radiatum-specific gamma. A critical but often overlooked factor is the dependence of CSD amplitude on the alignment reference. As demonstrated by Csicsvari et al. (2003), changing the reference electrode from the pyramidal layer to the granule cell layer reverses the apparent location of the dominant gamma sink (p. 313). If a study aligns LFP traces to the 'slow gamma' troughs on a radiatum electrode (forcing perfect constructive interference at that site), the resulting average will mathematically necessitate a maximal sink in radiatum, regardless of the true generator structure. Because many cited studies do not control for this 'self-fulfilling' alignment artifact, we remain skeptical that these profiles prove a unique layer-specific oscillator.

**3. Alternative Explanation for Radiatum Gamma** The reviewer asked for an alternative explanation for why gamma is observed in radiatum. In the Energy Cascade framework, this is interpreted as dissipation. The strong excitatory drive from CA3 (modulated by theta) creates substantial transmembrane current flow in radiatum dendrites. This drive recruits local feedforward and feedback inhibition. The resulting gamma-band field potential reflects the time constants of these local synaptic interactions (impedance) responding to the strong energetic drive, rather than the passive receipt of a 'gamma message' from CA3.

**4. Citation Corrections**

• **Lasztóczi & Klausberger (2014):** We thank the reviewer for catching our error. We have removed the claim that this study used optogenetics and have adjusted the text to correctly reflect that their conclusions were based on high-resolution spike-timing and CSD analyses.

• **Belluscio et al. (2012)**: We acknowledge the reviewer’s point that 'slow gamma' appears strongest in radiatum in their Figure 4C. We have refined our discussion to focus on the broader finding that gamma-band coherence is low across layers (as shown in our new Figure 5 from Berényi et al., 2014), which challenges the idea of a coherent channel, regardless of where the amplitude peaks."

Section 3.5 has been rewritten to explicate this information.

**Major 6. Line 429. The authors state that "theories of neural coordination that rely on spectral parcellation are challenged by a lack of direct empirical support that does not require inverse inference." It would be useful to discuss studies that move beyond purely inverse inference. For example, work showing that inhibition of EC inputs selectively disrupts mid-gamma in CA1 (PMC10894649; PMID: 24768692), as well as studies demonstrating that CA3-CA1 and EC-CA1 coherence is preferentially biased toward slow and mid-gamma bands, respectively (Colgin et al., 2009; PMID: 24023818).**

We thank the reviewer for raising these critical references. We agree that studies using optogenetic inhibition (e.g., Yamamoto et al., 2014) and optogenetic drive (e.g., Liu et al., 2023) represent an important methodological step beyond purely inverse inference. However, we argue that moving from correlation to perturbation does not automatically grant mechanistic insight if the perturbation forces the circuit into a non-physiological operating regime.

We have added a dedicated discussion of these studies in Section 3.6, arguing that they often conflate pathway necessity with frequency necessity. Our response to the specific papers is as follows:

1. **Pathway necessity versus frequency necessity (Yamamoto et al., 2014).** Yamamoto et al. employed continuous optogenetic silencing of MECIII terminals in CA1. This manipulation simultaneously removes (a) the informational content carried by MEC neurons, (b) the synaptic drive to CA1 pyramidal cells, and (c) oscillatory synchrony. While the resulting behavioral deficit demonstrates that the MECIII→CA1 pathway is necessary for task performance, it does not isolate oscillatory frequency as the causal variable. Removing a pathway necessarily eliminates both information transfer and its associated dynamics. As we now state explicitly in the revised text: “This manipulation removes informational content and synaptic drive alongside gamma synchrony, making it impossible to isolate the oscillatory component as the causal agent.”
2. **Forced non-physiological (“supernatural”) circuit states (Liu et al., 2023).** Liu et al. drove MEC interneurons at a fixed 53 Hz and interpreted the resulting disruption of endogenous mid-gamma as evidence for frequency-specific communication. We raise a fundamental biophysical concern with this interpretation. As articulated by Humphries (2017) and demonstrated empirically by Scarlett et al. (2004), forcing circuits to operate at fixed, externally imposed frequencies can dissociate field oscillations from natural cellular dynamics. Scarlett et al. showed that artificial stimulation produced apparently normal theta field potentials while profoundly disrupting the firing patterns of theta-ON and theta-OFF cells. We therefore argue that driving the circuit at a fixed frequency may force it into an unnatural operating mode (a 'supernatural' state), and that observed behavioral deficits may reflect this dissociation rather than the loss of a specific communication channel.
3. **Absolute coherence magnitude (Colgin et al., 2009).** Finally, regarding the reported preferential bias of coherence, we agree that MEC–CA1 coupling is biased toward higher gamma frequencies relative to CA3–CA1. However, the absolute magnitude of this coherence is typically low (≈0.2–0.25), indicating that approximately 80% of the variance remains independent across regions. We have revised the manuscript to explicitly question whether such weak coupling can support the proposed mechanism of frequency-specific routing.

Crucially, if coherence magnitude is accepted as the primary index of coordination, we note that **theta-band coherence** between these same regions is consistently much higher (>0.6–0.8; **Zhou et al., 2022**). This disparity reinforces our central argument: while gamma coherence is detectable, it is the slow rhythms (theta) that provide the dominant, high-fidelity coordinating structure across the network.

**Major 7. The methodological concern raised in Figure 4 is extremely interesting and deserves further development. As written, the argument risks coming across as "because theta harmonics exist, gamma oscillations cannot be real," which I don't think does justice to the point the authors are trying to make. The case would be much stronger if the authors proposed concrete, falsifiable tests to distinguish genuine gamma oscillations from harmonics. For example, one simple test would be to show that the slow-gamma component carries more energy than the first theta harmonics (16, 24 Hz). If the slow gamma is meaningful as a legit rhythm it should be at least more prominent than these harmonics; if it merely reflects higher-order theta harmonics, this would not be the case. In addition, do the authors predict that theta oscillations in CA3 are more non sinusoidal, potentially explaining why this region often shows stronger low frequency gamma components, as reported by PMID: 19934062 and Schomburg et al. (2014)? Similarly, PMC5585287 reports that the dentate gyrus is dominated by what is often labeled "slow gamma"; would this also be expected to correspond to less sinusoidal theta waveforms? Making such predictions explicit would help clarify the authors' position. More generally, it would be useful to complement Fourier-based analyses with approaches that do not rely on spectral decomposition. For instance, are there neurons in CA3 or DG whose ISI distributions or spike autocorrelograms show clear rhythmic structure at slow gamma timescales? Evidence at the level of spiking would go a long way toward clarifying whether these components reflect genuine oscillatory processes or arise from waveform nonlinearities.**

**In this context, it would also be relevant to understand how the authors interpret claims that slow gamma power decreases with running speed (Kemere et al., 2013, Fig. 3c), while theta nonlinearity is known to increase with speed (e.g., PMID: 27076421). Relatedly, what is their interpretation of reports of slow gamma activity in conditions where theta is weak or absent (e.g., PMID: 22920260)?**

We thank the reviewer for this deep and constructive methodological critique. We agree that our original framing risked being interpreted as 'because theta harmonics exist, gamma oscillations cannot be real,' which does not reflect our position. We have revised the manuscript to clarify that we do not deny the existence of high-frequency activity. Rather, our concern is with spectral parcellation—the interpretation of band-limited energy in the 20–50 Hz range as a discrete, independently generated oscillator in theta dominated states.

**1. Clarifying the claim** Our position is not that gamma is 'fake,' but that LFP power in the 20–50 Hz range is not, by itself, sufficient evidence for an independent slow-gamma generator. In non-sinusoidal regimes, harmonic structure and waveform asymmetry naturally produce spectral power in this band. Thus, harmonic peaks are not optional 'extra rhythms,' but the inevitable Fourier representation of waveform shape—creating an identifiability problem that requires explicit falsification.

**2. Concrete falsification tests** We agree that this distinction must be tested empirically. In the revised manuscript (**Section 3.6**), we propose two complementary, falsifiable criteria:

• **(i) Field-level test (Bicoherence):** The appropriate spectral test for distinguishing harmonics from independent oscillations is bicoherence, which assesses whether candidate components participate in stable phase-coupled frequency triplets. Power-based measures cannot make this distinction. We have added text noting that bicoherence analyses confirm that low-frequency 'gamma' structure shows strong harmonic phase coupling to theta, consistent with a non sinusoidal carrier rather than an independent generator (Sheremet et al., 2019).

• **(ii) Spike-level test (Non-spectral):** If slow gamma reflects a genuine rhythm relevant to computation, it should impose a corresponding neuronal timescale ('clock') observable in spike timing. in Figure 8, we analyze ISI distributions and spike autocorrelograms across CA1, CA3, and MEC. While we observe robust theta-timescale organization and fast intra-burst spiking, we find no consistent neuronal timescale in the slow-gamma range (20–50 ms), even in conditions where slow-gamma LFP power is prominent. This falsifies the hypothesis that slow gamma acts as a rhythmic pacer for these neurons.

**3. Regional predictions (CA3 and Dentate Gyrus)** The reviewer asks if our framework predicts that CA3 and DG should exhibit more non-sinusoidal theta. Yes. Because these regions receive stronger, more punctate synaptic drive than CA1, we predict they will exhibit sharper waveform asymmetries and thus richer harmonic structure extending into the 20–40 Hz range. This is fully consistent with reports of prominent low-frequency 'gamma' in these regions (e.g., Schomburg et al., 2014) and supports our view that such power reflects waveform shape rather than a distinct oscillator.

1. **The 'Kemere Paradox' and Non-Theta States** We agree that reports of slow gamma power decreasing with speed (Kemere et al., 2013) must be interpreted carefully. This observation presents a paradox for parcellation models: how can a signal ('slow gamma') whose power weakens with increased behavioral drive simultaneously serve as the dominant predictor of place cell spiking? In the Energy Cascade framework, this is resolved by waveform dynamics. As running speed increases, the theta waveform becomes more asymmetric (Sheremet et al., 2016). This redistributes energy across the spectrum, 'tightening' the harmonic structure and paradoxically reducing power in the broad 20–50 Hz inter-harmonic trough, even as the system becomes more energetic.

Finally, regarding non-theta states (e.g., sharp-wave ripples), the presence of 20–50 Hz power does not require a gamma oscillator. As shown by Oliva et al. (2018), increased power in this range often reflects the envelope dynamics and fusion of sharp-wave ripples rather than a separate gamma rhythm. We have added this citation to the manuscript to clarify the interpretation of non-theta states.

**Major 8. Below, I point out a few places where the citations do not quite line up with the claims being made, and where closer attention would strengthen the manuscript.**

**1. In line 60, the manuscript states: "The gamma band has often been further subdivided into slow (30-50 Hz) and fast (60-100 Hz) frequencies as well as slow-, mid-, and fast-variants (Belluscio et al., 2012; Lopes-dos-Santos et al., 2018)". However, the two studies cited here both divide hippocampal gamma into slow, mid, and fast components, with fast gamma extending largely above 100 Hz. As written, it is therefore unclear which literature the authors are referring to when they introduce the slow versus fast gamma dichotomy in the middle of the sentence. It could be Colgin et al. (2009), which claims "a slow gamma range (∼25-50 Hz) and a fast gamma range (∼65-140 Hz)". More generally, given the use of the term "often," I would expect this statement to be supported by citations from multiple laboratories or by a review. Clarifying the intended distinction and strengthening the citation base would help avoid confusion at this early stage of the manuscript.**

We thank the reviewer for noting this ambiguity in our original wording. We agree that the sentence as written could be read as conflating distinct classification schemes and did not sufficiently clarify which bodies of literature were being referenced.

We have revised the sentence to explicitly emphasize that multiple, partially incompatible gamma-band classification schemes coexist in the literature, including both slow–fast and slow–mid–fast frameworks, with frequency boundaries that vary substantially across studies and analytical approaches (now citing Colgin et al., 2009; Belluscio et al., 2012; Lopes-dos-Santos et al., 2018).

“The hippocampus has been a central testbed for spectral parcellation, especially models that assign distinct functional roles to "slow" and "fast" gamma during retrieval versus encoding. A fundamental problem immediately arises: the frequency boundaries used to define these bands vary substantially across studies (**Figure 2**). Colgin et al. (2009) defined slow gamma as 25–50 Hz and fast gamma as 65–140 Hz; however, other reports use different cutoffs. This lack of agreement is not a cosmetic issue. If band definitions shift to accommodate each new dataset, the framework loses falsifiable boundaries and becomes difficult to refute. In parallel, the field often performs a kind of "dual phrenology," mapping cognitive functions onto both frequency bands and anatomical subregions as if each mapping were independently diagnostic.”

This clarification aligns the text with the intent of Figure 2, which documents the historical and methodological variability in gamma definitions rather than presupposing a unified taxonomy. We believe this revision strengthens the manuscript by making explicit that the lack of consensus itself is a central empirical observation motivating our subsequent analysis.

**2. Line 62. The sentence reads: "Some propose that slow gamma coordinates memory recall and hippocampal-cortical interactions, while fast gamma supports sensory processing and memory encoding (Bieri et al., 2014; Colgin et al., 2009; Wang et al., 2025)." This appears to be an unintended misstatement. The cited studies do not claim that slow gamma coordinates hippocampal-cortical interactions; rather, they relate slow gamma to CA3->CA1 interactions. Unless the authors are referring to the "slow gamma" band described by Fernández-Ruiz et al. (2021), which is presumably related to LEC->DG interactions. However, this is not the classically defined slow gamma presumably associated with CA3->CA1 interactions and, to my knowledge, has not yet been replicated. I suspect the authors intended to attribute hippocampal-cortical interactions to fast gamma instead, which would be more consistent with the cited literature.**

We thank the reviewer for catching this imprecision. The original phrasing inadvertently conflated hippocampal–cortical interactions with CA3–CA1 interactions. The cited studies (Colgin et al., 2009; Bieri et al., 2014) specifically relate slow gamma to CA3→CA1 interactions and memory retrieval, whereas fast gamma has been associated with entorhinal–CA1 interactions and hippocampal–cortical coordination during encoding-related states. We have revised the sentence accordingly to reflect this distinction explicitly.

We also agree with the reviewer that the term 'slow gamma' has been applied to activity attributed to different anatomical pathways across studies (e.g., CA3→CA1 versus LEC→DG). We have revised the surrounding text to discuss this complexity explicitly, noting that such overlapping definitions may reflect a propagating rhythm through the pathway rather than independent oscillatory generators (citing Hsiao et al., 2016; Fernández-Ruiz et al., 2021).

**3. Paragraph starting at line 152. In this paragraph, the authors list three limitations of the BBS theory. The first two points are well taken: (1) gamma activity in macaque V1 is strongly dependent on stimulus properties (as also shown in PMC3001273), and (2) concerns about generalizability. The third point, however, concerning spike-leakage artifacts, seems conceptually misplaced in this list. Jia et al. (2013) (as well as PMC3075230) show that high-frequency LFP components are often heavily contaminated by broadband, spike-related signals. This is an important methodological caution, but it is not a direct critique of the BBS theory itself. Rather, it highlights the need to distinguish genuine narrow band gamma oscillations from broadband high-frequency power when interpreting LFP data, and therefore challenges how gamma is measured rather than the core theoretical claim that synchrony could support binding. More broadly, several substantive criticisms of BBS are not discussed here. For example, much of the foundational BBS literature was conducted in anesthetized animals using highly artificial stimuli, such as drifting gratings optimized for the recorded neurons, and gamma synchrony is often weak or absent under more naturalistic conditions. These limitations seem more directly relevant for assessing the explanatory scope of BBS than concerns about spike-leakage artifacts per se.**

We appreciate the reviewer's clarification and agree that the distinction between broadband spike contamination and narrow-band oscillations is primarily a methodological issue rather than a direct theoretical refutation of Binding by Synchrony (BBS). We have revised the text to make this distinction explicit.

However, we note that this methodological concern bears directly on the empirical grounding of BBS. The theory's central claims require the existence of identifiable, narrow-band gamma oscillations with precise phase relationships. If a nontrivial fraction of reported ‘gamma synchrony’ reflects broadband spiking activity rather than a genuine oscillatory process (Jia et al., 2013; Ray & Maunsell, 2011), then the evidentiary basis for BBS becomes ambiguous, not because synchrony could not in principle support binding, but because the phenomenon invoked by the theory may not be reliably isolated in vivo.

We agree that additional limitations more directly constrain the explanatory scope of BBS. Accordingly, we have expanded the discussion to emphasize that much of the foundational BBS literature relied on anesthetized preparations and highly artificial stimuli (e.g., drifting gratings optimized for recorded neurons). Under more naturalistic conditions in awake animals, gamma synchrony is often weak, intermittent, or absent (Brunet et al., 2014), raising questions about whether the precise synchronization observed in early studies reflects a general-purpose binding mechanism or a state dependent regime induced by specific experimental conditions.

Our revised text separates these issues conceptually while acknowledging that both how gamma is measured and the behavioral contexts in which it is observed are critical for evaluating the empirical support for gamma-mediated binding.

Section 3.2: “Significant limitations to the BBS model have since emerged. First, gamma oscillations in sensory cortex are highly dependent on stimulus properties. Henrie and Shapley (2005) demonstrated that gamma power and coherence in macaque V1 vary strongly with stimulus contrast and size, challenging the idea that gamma provides a general-purpose mechanism independent of sensory conditions. Similar stimulus dependence has been reported in subsequent work (Ray & Maunsell, 2010).

Second, concerns about generalizability remain. Gamma oscillations are robust in cats and some primates, yet they appear weaker and less consistent in humans (Hermes et al., 2015), raising questions about the extent to which findings from select animal models generalize across species.

A third major limitation involves ecological validity. Much foundational evidence for BBS was obtained under constrained conditions, including anesthetized preparations and artificial stimuli such as drifting gratings (Eckhorn et al., 1988; Gray et al., 1989). Under naturalistic conditions in awake animals, gamma synchrony is often weak, intermittent, or absent (Gieselmann & Thiele, 2008; Kayser et al., 2003; Thiele & Stoner, 2003) questioning whether precise synchronization reflects a general computational principle or a state-dependent regime induced by specific experimental contexts.

Interpretation of gamma synchrony is further complicated by methodological issues. High-frequency LFP components are often contaminated by broadband, spike-related signals (Jia et al., 2013; Ray & Maunsell, 2010). Because BBS requires identifiable, narrowband oscillations with precise phase relationships, such contamination complicates the isolation of the empirical phenomenon on which the theory relies.

Finally, causal evidence for gamma's role in perception remains limited. Histed and Maunsell (2014) demonstrated that optogenetically inducing gamma-frequency activity in macaque V1 increased spectral power but failed to improve, and in some cases disrupted, visual detection performance. While externally induced gamma may differ from endogenous rhythms, these findings highlight that replicating gamma spectral signatures is insufficient to invoke functional engagement. This underscores the necessity for caution when interpreting gamma activity as a causal driver of visual perception.”

**4. In the Spectral Multiplexing model, I was extremely confused. Consider the sentence: "This organizes the temporal windows within which faster oscillations (e.g., gamma, 30-100 Hz) can flexibly encode stimulus-specific information, effectively multiplexing signals within the same circuit to support cognitive functions (Bieri et al., 2014; Colgin et al., 2009; Lisman & Jensen, 2013)." None of the cited papers mention 'multiplexing' at all. In Colgin et al. (2009), "routing" refers to frequency-dependent biasing inputs to CA1, typically alternating across theta cycles (they claim a negative correlation). They claim this reflects a form of state-dependent input selection rather than multiplexing or the simultaneous routing of multiple independent information streams that require explicit demultiplexing mechanisms at the receiver (as in frequency-division coding schemes).**

**Akam & Kullmann (2014), which is cited immediately prior, is an influential review that does discuss oscillatory multiplexing. However, in that context they do not cite Colgin's or Jensen's papers; instead, they refer primarily to their own model (PMC3125699), which is widely known as the multiplexing model. Importantly, that framework does not rely on cross-frequency comodulation, making the remainder of the paragraph read awkwardly. My impression is that the authors are labeling Colgin-style frequency-dependent routing as multiplexing, but doing so creates confusion, since a distinct theory explicitly referred to as multiplexing exists and is cited in the same paragraph. Moreover, the authors write: "A key criticism of spectral multiplexing theories is that they risk being descriptively appealing but mechanistically underspecified." However, PMC3125699 explicitly presents a model showing how such multiplexing could, in principle, be implemented. The authors are of course welcome to critique this model or to argue that it lacks biological plausibility, but this would need to be done explicitly rather than in a vague or generic manner**.

We thank the reviewer for raising this important conceptual distinction. We agree that our original phrasing risked conflating related but distinct frameworks, and we have revised the manuscript to clarify terminology and theoretical commitments explicitly.

First, we acknowledge that **Colgin et al. (2009) describe frequency-dependent input biasing (“routing”) rather than oscillatory multiplexing in the strict engineering sense**. Their model emphasizes alternating dominance of inputs across theta cycles, not the simultaneous transmission of independent information streams requiring demultiplexing mechanisms at the receiver. We have revised the text to avoid labeling Colgin-style routing as multiplexing.

In contrast, **oscillatory multiplexing is explicitly proposed and formalized in the model of Akam & Kullmann (2014; PMC3125699)**. We agree with the reviewer that this framework provides a clear computational proof-of-principle and should be evaluated on its own terms. Our critique is therefore directed specifically at multiplexing models of this type, not at routing frameworks such as Colgin et al.

When evaluated against known hippocampal biophysics, we argue that the Akam & Kullmann multiplexing framework faces several specific constraints:

1. **Resonance properties** of hippocampal neurons are dominated by theta frequency dynamics, with gamma responsiveness that is broad and state dependent rather than narrowly tuned, limiting the feasibility of stable frequency selective inhibitory filters.

2. **Gamma activity in hippocampus is nested within theta**, appearing as transient, phase-locked bursts rather than as independent carrier oscillations, complicating frequency-division assumptions.

3. **Scaling limitations** arise under realistic convergence conditions, as acknowledged in Akam & Kullmann’s own simulations, whereas hippocampal circuits operate in high fan-in regimes.

4. **Gamma reflects circuit state rather than initiating routing**, as routing in these models requires prior parameter changes, with oscillations emerging as a consequence rather than a causal gate.

These points are now stated explicitly in the manuscript. We believe this revision resolves the reviewer’s concern by clearly distinguishing routing from multiplexing, acknowledging formal multiplexing models, and articulating specific biological constraints on their applicability to hippocampal gamma dynamics.

**5. The sentence "Kemere et al. (2013) found that both slow and fast gamma power increased during memory encoding" is not precise. Kemere et al. show that power in both gamma bands is elevated during exploration of novel environments relative to familiar ones, which is not equivalent to demonstrating a direct increase during memory encoding. That said, the broader point the authors are making remains valid, but the support from this study is more indirect than the current wording implies.**

We thank the reviewer for this excellent point regarding precision. We agree that Kemere et al. (2013) specifically demonstrated that gamma power is modulated by environmental novelty rather than providing a direct measure of memory encoding. We appreciate the distinction, as it prevents over-interpreting the physiological state as a functional one.

We have revised the text to accurately reflect their findings:

“ Kemere et al. (2013) reported that power in both slow and fast gamma bands was elevated during exploration of novel environments compared to familiar ones, consistent with state dependent modulation rather than frequency-specific encoding. "

**6. Line 296. "Multiple influential studies propose that CA3 and MEC generate distinct gamma frequencies (Colgin et al., 2009; Fernández-Ruiz et al., 2017; Schomburg et al., 2014; Zheng et al., 2016), yet none demonstrate that these upstream cells intrinsically oscillate at the attributed frequencies." The first work reporting slow-frequency gamma in CA3 (they call it 'low' gamma) is not acknowledged (PMC2791641). That said, I agree with the authors' main point: these studies rely largely on LFP analyses and do not show that spiking in upstream populations intrinsically expresses rhythms at those frequencies.**

We thank the reviewer for highlighting this important omission. We agree that Tort et al. (2009) represents the foundational report of low-frequency (‘slow’) gamma activity in CA3, and we have updated the manuscript to explicitly cite this work alongside Colgin et al. (2009) and subsequent studies. Importantly, while Tort et al. demonstrated gamma band structure in CA3 LFPs, this finding (like later work) does not establish that upstream CA3 neurons intrinsically oscillate at these frequencies at the level of spiking, which remains the central point of our critique. We appreciate the reviewer’s agreement on this distinction.

**7. Line 373. The sentence "Oriens-lacunosum moleculare (O-LM) cells … are essential for theta-gamma coupling (Tort et al., 2007)." cites a purely computational (modeling) study as if it provided experimental evidence.**

We thank the reviewer for this important correction regarding the nature of the evidence in Tort et al. (2007). We agree that our previous phrasing inadvertently implied this was an experimental finding rather than a computational prediction. We have revised the text to explicitly attribute this insight to modeling work.

*Revised text:*

“Computational models suggest that Oriens-lacunosum moleculare (O-LM) cells, which project to distal dendrites in stratum lacunosum-moleculare, can play a critical role in coordinating theta–gamma interactions at the circuit level (Tort et al., 2007)."

**Minor comments:**

**Minor 1. In the CTC paragraph the authors overlook several important criticisms regarding this hypothesis. They do mention the evidence is correlational but they don't spell out. In a few sentences they could mention that much work has shown that coherence is very likely a consequence of communication rather than the cause (explicitly studied here PMC8691951).**

We thank the reviewer for this excellent suggestion. We have substantially expanded our critique of the Communication Through Coherence hypothesis to incorporate the important findings from Schneider et al. (2021, Neuron).

Specifically, we now explicitly state that "coherence is a consequence rather than a cause of communication" and detail the mechanistic evidence supporting this conclusion. The revised paragraph now includes:

1. **Empirical evidence** from simultaneous recordings in macaque fronto-parietal cortex and mouse LGN-V1 demonstrating that coherence depends lawfully on anatomical connectivity and sender power rather than representing an active gating mechanism.

2. **Causal evidence** from optogenetic silencing experiments and cortical DOWN state analyses showing that afferent synaptic inputs—not spike entrainment in the receiver—are the principal determinant of coherence.

3. **State-dependent changes** in coherence explained entirely by changes in sender dynamics rather than modulation of synaptic gain or effective connectivity.

4. **Specific confounds** in attention-related coherence studies, including changes in firing rates, stimulus drive, and phase-locking properties of projection neurons that can independently modulate coherence without changes in communication efficacy.

This addition strengthens our argument that observed correlations between coherence and behavior do not establish coherence as a causal mechanism for communication, which aligns with our broader theoretical framework that gamma oscillations reflect metabolic/circuit constraints rather than evolved computational mechanisms.

**Minor 2. InSection 4.3, the authors should highlight studies that have shown experimentally that downstream neurons integrate inputs over fairly long temporal windows, challenging the realistic function of spike synchrony (PMC3890892).**

We thank the reviewer for this excellent suggestion. We have now incorporated the findings from Histed & Maunsell (2014, PNAS) into Section 4.3 to strengthen our argument that downstream neurons integrate inputs over extended temporal windows, challenging the functional relevance of spike synchrony and spectral multiplexing.

Specifically, we added a new paragraph immediately following our introduction of rate based coding that details the key experimental findings:

1. Linear integration over ~100ms windows - behavioral detection depended solely on total spike count, not temporal arrangement of inputs

2. No behavioral advantage for synchrony - synchronous inputs concentrated into 1-3 ms periods provided no advantage over sustained inputs of equivalent total power spread across 100 ms

3. No frequency-specific routing - pulse trains tested across beta through gamma frequencies (10-50 Hz) showed no frequency-dependent effects on detection performance

4. Population rate coding with weak inputs - behavioral responses were driven by many neurons with small rate changes (~1.1 spikes/s average) rather than strong synchronous bursts

These findings provide direct experimental evidence that downstream neurons integrate inputs over extended temporal windows and that precise spike timing or frequency specific synchrony confers little to no behavioral advantage under these conditions. This substantially constrains the functional role that spike synchrony and spectral multiplexing can plausibly play in downstream readout. These results are naturally accommodated by rate-based frameworks, including Binding by Rate Enhancement, without requiring frequency-specific routing mechanisms.

2nd Decision letter

### **Reference:** CRNEUR-D-25-00019R1

### **Title:** Spectral Dependence as a Framework for Neural Coordination

### **Journal:** Current Research in Neurobiology

Dear Ms Besosa,

Thank you for resubmitting your manuscript to Current Research in Neurobiology and for your efforts in addressing the previous round of reviewer comments.

We have completed our evaluation of your revised manuscript. As you will see from the reports, while many of the reviewers’ concerns have been addressed, some outstanding points remain. In particular, I would like to highlight Major Comment 7 from Reviewer 2, which will require careful consideration in a revision.

I therefore invite you to resubmit your manuscript after addressing the comments below. Please resubmit your revised manuscript by April 17, 2026.

When revising your manuscript, please consider all issues mentioned in the reviewers' comments carefully: please outline every change made in response to their comments and provide suitable rebuttals for any comments not addressed. Please note that your revised submission may need to be re-reviewed.

To submit a revision, go to https://www.editorialmanager.com/crneur/
and log in as an Author. You will see a menu item call Submission Needing Revision. You will find your submission record there.

When you submit the revised manuscript, please provide a separate document, uploaded as 'Detailed Response to Reviewers,' that carefully details, point-by-point, the list of changes made in response to the reviewers' comments. You may also include a suitable rebuttal to any specific request for change that you have not made. The journal strongly encourages you to submit two versions of your revised manuscript in order to facilitate the evaluation process: one with changes in the manuscript marked (upload as file type: Manuscript (REVISED, text with changes Marked)), in addition to an unmarked, production-ready version (required) (upload as file type: Manuscript).

Current Research in Neurobiology values your contribution and I look forward to receiving your revised manuscript.

Kind regards,

Ipshita Zutshi
Editor
Current Research in Neurobiology

**Comments from Editors and Reviewers:**

**Reviewer #1:**

The authors have done an excellent job with this revision, and I greatly appreciate the extent of the work that went into it. This is not a superficial update, but a substantial reworking of the manuscript that has made the central argument much clearer and more compelling. The revised version is more focused, with the hippocampus now serving as a much clearer primary exemplar system rather than the manuscript moving back and forth between hippocampal and cortex-wide claims. I also think the new schematics and graphical framing are a major improvement. The energy cascade framework is now presented in a much more accessible and concrete way, and the figures do a far better job of positively communicating the authors' model rather than primarily critiquing alternative accounts. In addition, the manuscript is strengthened by the inclusion of new analyses and by the explicit articulation of falsifiable predictions, which make the framework more rigorous and useful to the field.
Overall, this now reads as an ambitious, carefully argued, and impressively comprehensive piece that will likely become an important reference for discussions of oscillations and neural coordination. I only have a few remaining minor comments/ suggestions/ points of discussion.
1. Figure 5 / laminar coherence figure: I know this is a reproduced figure, but it would be nice to label it a bit more clearly here. For a reader who is not already very familiar with hippocampal laminae, it is difficult to immediately understand which regions/layers are being compared. Better labeling of the layers and clearer visual guidance would make the figure much easier to interpret.
2. Spike timing / ISI analysis figure: I thought this was a very interesting result and a strong addition to the manuscript.
3. Lines 1158-1162: One potential result to consider is the EC lesions in Bragin et al 1995. That study reported that entorhinal lesion decreased theta and increased gamma in radiatum. If so, it would be useful either to discuss how that finding fits with the present framework or to clarify why it does not falsify the argument here.
4. Possible future tests involving septal manipulations: I do not view this as necessary for the current manuscript, but one potentially interesting line of discussion would be to consider datasets in which theta has been manipulated more directly through the septum, for example by inhibiting cholinergic drive, optogenetically pacing theta, or cooling the septum. Predicting what should happen when theta frequency versus theta amplitude is altered could be interesting future steps.

**Reviewer #2:**

The authors have clearly invested substantial effort in revising this manuscript, and I appreciate the constructive way in which they engaged with the previous round of feedback. The manuscript has improved in several important respects, particularly in clarity and scope. In this second round, I have aimed to maintain the same level of rigor and depth in evaluating the revised arguments. While some remaining points reflect differences in interpretation, others concern issues that, in my view, require further clarification or revision. The length of this report reflects the extent of the changes introduced in the revision and is intended as a careful and respectful engagement with the authors' work. I leave it to the editors to weigh the necessity of each point for acceptance, but I would like to highlight that I remain particularly concerned about the issues raised in Major Comment 7, as well as the way the "parcellation" framework is characterized throughout the manuscript.

In particular, I believe the manuscript currently conflates conceptually distinct positions within what is referred to as "spectral parcellation." Several of the cited studies (e.g., Fernández-Ruiz et al., 2023; Lasztóczi & Klausberger, 2016; Lopes-dos-Santos et al., 2018) do not propose that gamma oscillations play a mechanistic role in communication, but rather that band-limited activity can serve as a proxy for pathway engagement. This distinction is critical. In principle, upstream circuits with different temporal structure could impose separable spectral components on downstream LFPs without those oscillations functioning as causal communication channels or entraining downstream spiking. Under this interpretation, gamma-band activity can be used as an indirect readout to test the contribution of specific pathways, rather than as the mechanism mediating communication itself, a point explicitly acknowledged in Fernández-Ruiz et al. (2023). By contrast, stronger claims such as those originally suggested by Colgin et al. (2009) suggest a functional role for gamma in routing or transmission. These are qualitatively different levels of interpretation, and treating them as a single unified "parcellation" position risks misrepresenting the literature. It would strengthen the manuscript to explicitly distinguish between these layers of interpretation before critiquing them.

Regarding 'Major 1': the presentation of the Energy Cascade Model.

As with several other sections, Section 4.1 has been substantially revised. The authors make a clear effort to more precisely articulate the "Energy Cascade Model for Neural Computation," in part by contrasting it with alternative frameworks. Despite this, I found that several aspects of the model remain unclear. Clarifying these points would improve both interpretability for the reader and accessibility for a broader audience.

In the segment stating that phenomena such as "oscillations," "bursts," or "cross-frequency coupling" are not independent signals but structured patterns arising from circuit dynamics, the authors appear to suggest that alternative frameworks treat these phenomena as independent signals exchanged between circuits. It is not clear that this accurately reflects most existing theories, which generally also interpret such activity as emerging from circuit properties, including connectivity, cellular composition, and intrinsic biophysics. The intended distinction may instead be that some frameworks operate at a higher level of abstraction, potentially underemphasizing biophysical constraints or treating frequency bands as functionally separable units. If so, this distinction should be stated more explicitly and justified. Most importantly, how these assumptions arise and in what sense they are intrinsic to those frameworks. As written, the contrast risks being interpreted as a straw-man characterization.

In addition, it would be helpful for the authors to specify more clearly which frameworks they see as contrasting with their own along this dimension. For example, the Fernández-Ruiz et al. (2023) review, cited here as an example of a "frequency parcellation" framework, explicitly states that "a given gamma oscillation does not per se implement any specific function (e.g., attention or memory recall), but it rather reports the underlying computations and communication channels for information processing". This appears closely aligned with the authors' own statement that "gamma oscillations mark circuit activation shaped by excitatory-inhibitory balance rather than specialized cognitive signals." I understand that the authors' critique of Fernández-Ruiz et al. may instead concern the proposed functional or causal role of oscillations in communication. However, it is not clear why such "parcelation" views require oscillations to be treated as independent signals, detached from the physiological constraints of the underlying circuits.

My understanding from the revised Section 4.3 is that the cascade model proposes a hierarchical organization across multiple scales, with theta oscillations providing the dominant low-frequency drive. Within this framework, theta defines phases of relatively higher and lower network excitability (e.g., via disinhibition or net excitatory input). During high-excitability phases, increased synaptic drive recruits local excitatory-inhibitory circuit motifs, from which gamma rhythms emerge. In this view, gamma reflects a consequence of local circuit dynamics rather than an independent process. These gamma dynamics, in turn, impose finer-scale structure on neuronal spiking, with higher-frequency components emerging from this modulation, not necessarily as distinct oscillations.

Please correct me if this is not an accurate synthesis. If I am missing something, it would be helpful to make this clearer in the text.

Assuming this interpretation is broadly correct, it is not clear that the cascade model is mutually exclusive with the views the authors describe as "frequency parcellation." It is well established that driving circuits with excitatory-inhibitory loops using a theta-paced input can generate nested gamma activity, including in canonical network models. Within parcellation frameworks, differences in circuit properties (such as conduction delays, cellular composition, or intrinsic dynamics) could naturally give rise to variation in gamma frequency across circuits under a shared low-frequency drive. In this scenario, gamma would still covary with the strength of the underlying drive. It would therefore be helpful to clarify whether the authors intend to argue that these frameworks are fundamentally incompatible, or instead that there is insufficient empirical evidence for distinct circuits to produce reliably separable, non-overlapping gamma bands.

It would also be helpful to clarify how the framework accounts for the presence of a "mid-gamma" component at the peak of CA1 pyramidal layer theta, a phase typically associated with lower excitability. If gamma reflects the recruitment of local excitatory-inhibitory dynamics by increased drive, one might expect it to be strongest during phases of maximal excitation. While "fast gamma" (if treated as a genuine oscillation rather than a spike-related component) does align with this expectation, it remains unclear how the framework accommodates the coexistence of multiple gamma bands within the same circuit. Several studies (e.g., Schomburg et al.; Lasztóczi & Klausberger), using different analytical approaches, report that fast gamma is largely localized to the pyramidal layer, whereas mid-gamma is strongest in stratum lacunosum-moleculare. It would be useful for the authors to explain how such laminar and spectral dissociations are interpreted within the cascade framework, particularly given that fast gamma is substantially lower amplitude than mid-gamma.

A clearer contrast between the cascade framework and what the authors term "frequency parcellation" is presented in the paragraph describing gamma as a "spectral front" that shifts with increasing drive. If this is intended as a fundamental distinction, it risks overstating the contrast. In hippocampal contexts, parcellation models can be interpreted more modestly: different upstream circuits may exhibit distinct gamma-band activity due to differences in their network properties. When such inputs converge onto a common target, they could generate synaptic currents with different temporal structure, which would be reflected in the downstream LFP even with no entrainment of downstream spiking activity (see Buzsáki & Schomburg, 2015; Schneider et al., 2021). In this scenario, partially overlapping gamma bands may serve as indicators of pathway engagement without requiring local neurons to be entrained to those rhythms. It would therefore be helpful to clarify whether the authors consider this scenario biophysically implausible, or whether their argument is instead that current empirical evidence does not support it.

Some parts of the text remain difficult to interpret in concrete terms. For example, the concept of fractality is introduced without a clear explanation of what it corresponds to at the level of neural circuits or cellular organization. The statement that the hippocampal formation exhibits self-similar structural complexity with a fractal dimension of ~2.5 is presented without clarifying what this value implies biologically or functionally. It would be helpful to explain whether this specific value is informative for the proposed framework, and how different values would affect its interpretation. More generally, the role of fractal scaling within the model would benefit from clearer articulation.

The sentence "Metabolic constraints further limit sustained high-frequency activity, while inhibitory feedback and refractory dynamics introduce history-dependent temporal filtering at the circuit level" requires a reference, and it is vague.

This claim also needs a reference: "Axonal delays across hippocampal-entorhinal and cortical circuits routinely span 10-50 ms, rendering millisecond-scale oscillations such as gamma (~10-20 ms period) ill-suited for global phase alignment." At least for the hippocampal-entorhinal example.

Regarding 'Major 2', New section 4.4:
First of all, the authors must revisit how the reference Schomburg et al., (2014) is used in the segment "Regions characterized by strong, punctate synaptic drive, such as CA3 and the dentate gyrus, exhibit greater theta waveform asymmetry and consequently more prominent harmonic power in the 20-50 Hz range. This accounts for the prevalence of 'slow gamma' reports in these regions (Schomburg et al., 2014)...". In the referred study, the authors explicitly note that modulation BELOW ~30 Hz may reflect theta harmonics or waveform asymmetry, and they apply high-pass filtering (>30 Hz) to remove these components. In their own words "We therefore high-pass filtered (>30 Hz) the LFP traces to eliminate the large amplitude theta and its first few harmonics from signals recorded during RUN and REM". Importantly, their analysis treats radiatum associated slow gamma in the 30-80 Hz (not particularly narrow) range as a pathway-specific oscillatory component associated with CA3 input, rather than as a byproduct of theta harmonics. As such, the cited work does not support the interpretation proposed here.

In my view, Prediction 1 lacks discriminative power. As noted above, it is a basic and well-established principle that increasing the level of drive to a network with excitatory-inhibitory loops leads to an increase in the amplitude of the emergent gamma oscillation. In this sense, the prediction does not appear to distinguish the proposed framework from competing models. Unless the authors mean that oscillations above a certain frequency cannot exist without an underlying drive that is itself necessarily oscillatory, this prediction seems limited. At times, I get the impression that the authors may be making this stronger claim, but it is not stated explicitly. If this is indeed the case, it would constitute a strong and testable prediction. Would the authors argue that it is not biophysically possible to generate gamma oscillations in a neural circuit in the absence of an underlying low-frequency rhythm? If so, how does this reconcile with gamma oscillations generated in vitro (e.g., PMC196924; PMID: 9671302)? Would these always be expected to be modulated by theta or another slower rhythm? Similarly, how does the framework account for findings from the primate visual cortex, where persistent gamma oscillations can be driven by visual stimuli (e.g., gratings) without an obvious low-frequency component? Are such oscillations predicted to be necessarily modulated by slower rhythms at the single-trial level? In the hippocampus, the so-called beta2 rhythm (23-30 Hz) (Berke et al., 2008; also reported by Tort's and M.X. Cohen's labs) has been shown to anticorrelate with theta. Although described as "transient" in the original work, it persists for multiple cycles and, during the first minutes of novelty, can exceed theta in amplitude. How does this observation fit within the proposed framework? Would beta2 in this case take the role of the slower organizing rhythm? If so, how flexible is the model with respect to what constitutes the "slow" component?

Prediction 2 is stronger. If I understand well, it claims that multiple gamma oscillations cannot exist in a given circuit. Therefore, manipulation experiments cannot get rid of one gamma component in isolation. The sentence "Conversely, CA3 inactivation, predicted by routing models to abolish "slow gamma" (30-50 Hz), produces no significant reduction in any gamma range" needs a reference with experimental evidence.

Prediction 3. It would be helpful if the authors could clarify why increased asymmetry of the low-frequency component is naturally expected to lead to increased gamma power within the cascade framework (excluding potential contributions from harmonic artifacts). Is the underlying intuition that a more asymmetric theta waveform concentrates synaptic drive into a shorter time window, thereby delivering more energy at once to local excitatory-inhibitory loops? From my understanding, this would be a very elegant prediction.

Prediction 4 would benefit from further clarification. The statement that models positing independent gamma channels predict relative preservation of gamma structure under such manipulations is difficult to interpret in concrete terms. For example, in the canonical CA3-EC-CA1 system, where theta is present across regions, what would it mean experimentally to "disrupt theta"? More generally, it is not clear that this prediction cleanly distinguishes the proposed framework from alternative models. Even in frequency parcellation accounts, gamma activity depends on synaptic drive and local excitatory-inhibitory dynamics, so perturbations of low-frequency input would also be expected to affect gamma. It would therefore be particularly helpful for the authors to describe a concrete experimental scenario (e.g., replacing rhythmic input with tonic drive in a defined pathway) and to specify the distinct outcomes predicted by each framework.

Regarding Major 3, I appreciate the authors' discussion of the PV⁺ and SST⁺ manipulation study. However, the first part of my comment was not addressed; specifically, the possibility that two upstream circuits converging onto the same downstream target may exhibit partially distinct gamma activity, such that their synaptic inputs could contribute separable components to the downstream LFP. This alone may be sufficient to justify the use of gamma-band activity as a proxy for pathway engagement. In the hippocampus, for example, gamma components resembling those observed in upstream regions (e.g., EC and CA3) appear to align with their respective dendritic targets in CA1 (stratum lacunosum-moleculare and stratum radiatum), and occur at corresponding theta phases associated with activity in those inputs. It would be helpful for the authors to clarify how this possibility is accounted for within the proposed model: if upstream populations exhibit distinct gamma-band activity, their synaptic inputs to a shared downstream target would be expected to be temporally structured at those respective frequencies, potentially giving rise to multiple gamma components in the resulting LFP when these inputs are not spectrally identical.
In this section of the rebuttal letter, the authors again cite Schomburg et al. (2014) in a way that is difficult to reconcile with the original study. The statement that activity in t30-50 Hz ranges may reflect theta harmonics is not supported by that work. Schomburg et al. explicitly note that components below ~30 Hz may be contaminated by theta harmonics and apply high-pass filtering (>30 Hz) to remove these contributions. Their subsequent analyses treat activity above 30 Hz as reflecting a distinct oscillatory component. As such, using this reference to support the interpretation that 30-50 Hz activity reflects theta harmonics is problematic and should be revised or more carefully qualified. I comment more on the harmonics issue in my comments on Major 7 below.


Regarding Major 4, I find the authors' emphasis on the lack of mechanistic explanation to be a strong and valuable point. The literature in this area is often largely descriptive, and statements such as "any more than observing theta in multiple regions implies multiple types of theta oscillation" capture this issue particularly well. I would have welcomed a stronger focus on this line of argument, although I understand the authors' preferred to focus on other arguments. In contrast, I'm still not convinced by the utility of the associated figure. As noted previously, simply illustrating variability in how gamma bands are defined does not substantially advance the mechanistic critique. In addition, at least one citation issue remains unresolved (as I wrote before: "Of note, Lopes et al. define fast gamma largely above 100 Hz (tSC5), rather than around 80 Hz as implied in your Figure 1"); however this is relatively minor compared to other citation concerns raised elsewhere.


Regarding Major 5, point 1 ("Anatomy constrains location, not frequency") is well written, and I have no further comments.
Point 2 requires correction. I agree that CSD analyses can be problematic when a specific channel is used as the reference for triggering LFP averaging before computing CSD. However, none of the studies I cited (Schomburg et al. (2014) or Lasztóczi & Klausberger (2016)) used that procedure. Schomburg et al. used ICA to decompose multivariate silicon probe recordings and identified the slow-gamma component from the component with strongest loadings in radiatum. Lasztóczi & Klausberger (2016) computed CSD directly from multichannel LFP recordings and then derived gamma components from those data. In other words, the artifact you describe does not apply here.
Point 3 appears to diverge from the interpretations proposed by Buzsáki & Schomburg (2015) and Schneider et al. (2021). This is entirely reasonable and could represent a valuable contribution, but it should be stated explicitly. Both studies argue that gamma-paced synaptic inputs from upstream circuits can be reflected in downstream LFPs, and therefore caution against interpreting LFP-LFP or spike-LFP coherence as direct evidence of effective communication. If the authors instead argue that observing oscillatory structure in a downstream region requires engagement of local circuit dynamics, this constitutes a clear departure from those models and should be explicitly framed as such.


Regarding Major 6, I do not find the argument based on low coherence values compelling. Measured coherence is strongly influenced by signal-to-noise ratio, and theta being a slower, more global signal is likely to have a higher SNR than gamma, which is faster and more spatially localized. Further, absolute coherence values in the range of ~0.2-0.25 being labeled as "low" is somewhat subjective. Moreover, neuronal firing is determined by multiple factors, and gamma coherence would not be expected to account for all of them. That said, I recognize that this line of argument is considered persuasive by many in the field, and I raise these points primarily to encourage scientific discussion rather than as a central objection.
Regarding the authors' item 2, I agree with what the authors outline but it remains unclear to me how perturbation of entorhinal cortex activity SELECTIVELY affects mid-gamma in CA1 stratum lacunosum-moleculare, while leaving fast gamma in the CA1 pyramidal layer and slow gamma in CA1 radiatum relatively unaffected.


Regarding Major 7, this is the section where I have the most difficulty with the authors' rebuttal. I agree with the central point that energy in a frequency band does not, by itself, demonstrate the existence of an independent oscillator, and that asymmetric theta waveforms naturally generate harmonics. I also appreciate the addition of the analysis in Figure 8. In particular, the absence of slow-gamma structure in spike timing is an important result. It would strengthen the manuscript to frame this more explicitly as a falsification-relevant test: namely, that the presence of a consistent slow-gamma timescale in ISIs would constitute credible evidence for a generator, but we do not observe it. At present, this result is presented more as supportive evidence for the harmonics interpretation rather than as a clear criterion that could, in principle, have supported the opposing view. Making this explicit would clarify that the authors' position remains open to counter-evidence, but that such evidence is not found in the data.

I find the bicoherence-based argument less compelling, as it relies on more complex analytical assumptions. For example, methods such as EMD have been proposed to separate nonlinear waveform components and mitigate harmonic artefacts, but there is no clear mathematical guarantee that they are fully free from such confounds as far as I know. A similar concern applies to bicoherence-based approaches. For this reason, I would encourage the authors to emphasize simpler, more mechanistically interpretable tests where possible. In that context, one suggestion from my previous review was ignored: a direct comparison of harmonic power (e.g., testing whether putative slow-gamma components exceed the power of lower-order theta harmonics at 16 and 24 Hz). If slow gamma reflects a higher-order harmonic, it would not be expected to carry more energy than preceding harmonics. Would the authors agree that this provides a straightforward and complementary test? If not please let me know why for the sake of my own curiosity. Anyway, including such simple, interpretable analyses could further strengthen the argument.

Regarding the explanation of the "Kemere paradox," I find the proposed mechanism difficult to reconcile with the expected spectral consequences of increased theta asymmetry. If slow-gamma-range power is largely attributable to theta harmonics, then increasing theta asymmetry (and amplitude) would generally be expected to enhance harmonic structure, including components within the 20-50 Hz range. While it is possible that energy becomes more concentrated into narrower harmonic peaks or redistributed outside this band, it is not immediately clear that such a process would produce a net decrease in band-limited power under physiologically plausible conditions. To examine this directly, I simulated myself asymmetric theta waveforms (Abreu et al., 2010). In this framework, increasing asymmetry via faster rise times (consistent with hippocampal theta) redistributes energy into higher harmonics and, when total energy is controlled, tends to increase rather than decrease power in the 20-50 Hz range. Achieving a net decrease in this band required waveform shapes with very sharp, non-physiological features (e.g., near-sawtooth profiles), suggesting that the proposed explanation may not be sufficient on its own. Taken together, this raises the possibility that the observed decrease in 20-50 Hz power with running speed cannot be explained solely by increased theta asymmetry. If the authors propose that asymmetry alone is sufficient, it would be helpful to demonstrate this explicitly, for example using a physiologically constrained waveform model of theta asymmetry.

The passage beginning "This divergence illustrates a broader vulnerability noted by Buzsáki (2020)…" gives the impression that the specific critique (flexible selection of frequency bands and their mapping onto anatomical pathways) is directly mentioned in the cited article. However, Buzsáki (2020) does not make this argument in those terms, but rather presents a broader critique of top-down mappings between psychological constructs and neural correlates. As written, the text risks attributing a more specific claim to Buzsáki (2020) than is explicitly stated. It would be helpful to clarify that the extension to what might be termed "spectral phrenology" reflects the authors' interpretation rather than a position directly advanced in the cited work.

2nd Author Response Letter

**Response to comments from Editors and Reviewers:**

Dear Editors and Reviewers,

We are pleased to submit the revised version of our manuscript and would like to express our sincere gratitude for the continued time, care, and intellectual engagement devoted to this review process. We especially appreciate the exceptionally thorough and constructive nature of the feedback we received across both rounds of revision. The reviewers’ comments have substantially strengthened the manuscript, and we found their critiques both rigorous and genuinely helpful in clarifying our arguments, refining the presentation, and broadening the conceptual scope of the paper.

We have carefully considered each suggestion and recommendation and made every effort to meet or exceed the expectations articulated in the reviews. In response to the reviewers’ prior recommendations, we previously re-centered the manuscript around the energy cascade / spectral dependence framework, strengthened its mechanistic grounding, and added figures to improve accessibility. We also introduced new empirical analyses and schematics, articulated explicit falsifiable predictions, and expanded the framework beyond the hippocampus to neocortex. In this second revision, we have continued that process by sharpening the logic of several contested sections, improving transitions and framing, clarifying terminology, and ensuring that our central claims are presented with greater precision and balance.

Reviewer 2 expressed concern that our treatment of the parcellation literature risked misrepresenting the positions we critique. We took this concern seriously, and it is the primary reason Section 3.8 relies heavily on direct quotation. The central argument of that section is that portions of the parcellation literature maintain incompatible positions simultaneously, alternating between mechanistic and proxy claims depending on which is under challenge. This is a strong claim, and we do not believe it can be established responsibly through paraphrase alone, since paraphrase itself invites the concern that we are substituting our interpretation for the authors’ actual claims. By quoting the primary sources directly, we allow readers to evaluate these tensions in the authors’ own language and without interpretive mediation.

For example, the same paper (Fernández-Ruiz et al., 2023) states that gamma does not implement “any specific function,” while also claiming that gamma inputs “determine the precise timing of action potential discharge.” The same article refers to gamma oscillations as “elementary units of collective neural activity” while also acknowledging that they are not “real” physical units. These tensions are not created by our framing; they are present in the source text itself and become visible most clearly when the original wording is preserved. Similarly, our discussion of Lopes-dos-Santos et al. (2018) depends on showing that their own reported result—spectral component strengths lying on “a multidimensional continuum rather than clustering into non-overlapping subsets”—sits uneasily beside an interpretive framework of “flexible switching between different operating modes.” In such cases, the quotations function as primary evidence, with each included to document a specific claim that our analysis then evaluates on its own terms. We believe this is the most rigorous way to address the concern of possible misrepresentation: rather than telling the reader what these papers claim, we show them directly.

More broadly, we have worked throughout this revision to ensure that criticism of existing frameworks is paired with constructive theoretical development. The manuscript now places even greater emphasis on explanatory alternatives, empirical testability, and synthesis, while preserving the critical analysis necessary to motivate those advances.

We are deeply appreciative of the time and expertise the reviewers and editors have devoted to our manuscript. Their thoughtful engagement has materially improved the quality, clarity, and rigor of the work. We hope that the revisions satisfy all remaining concerns and that the manuscript is now suitable for publication.

Thank you again for your thoughtful evaluation and for the opportunity to revise and resubmit.

Sincerely,

The Authors

**Reviewer 1:**

**The authors have done an excellent job with this revision, and I greatly appreciate the extent of the work that went into it. This is not a superficial update, but a substantial reworking of the manuscript that has made the central argument much clearer and more compelling. The revised version is more focused, with the hippocampus now serving as a much clearer primary exemplar system rather than the manuscript moving back and forth between hippocampal and cortex-wide claims. I also think the new schematics and graphical framing are a major improvement. The energy cascade framework is now presented in a much more accessible and concrete way, and the figures do a far better job of positively communicating the authors' model rather than primarily critiquing alternative accounts. In addition, the manuscript is strengthened by the inclusion of new analyses and by the explicit articulation of falsifiable predictions, which make the framework more rigorous and useful to the field.**

**Overall, this now reads as an ambitious, carefully argued, and impressively comprehensive piece that will likely become an important reference for discussions of oscillations and neural coordination. I only have a few remaining minor comments/ suggestions/ points of discussion.**

We sincerely thank the reviewer for this generous assessment and are grateful for their constructive engagement across both rounds of review, which has substantially strengthened the manuscript.

**1. Figure 5 / laminar coherence figure: I know this is a reproduced figure, but it would be nice to label it a bit more clearly here. For a reader who is not already very familiar with hippocampal laminae, it is difficult to immediately understand which regions/layers are being compared. Better labeling of the layers and clearer visual guidance would make the figure much easier to interpret.**

We have revised the Figure 5 caption to provide additional anatomical guidance. Because this is a reproduced figure from Berényi et al. (2014) and the original publication does not label individual laminae on the coherence panels, we have limited our annotations to what can be verified from the source material.

**2. Spike timing / ISI analysis figure: I thought this was a very interesting result and a strong addition to the manuscript.**

We thank the reviewer for this positive feedback and are glad this analysis strengthens the manuscript.

**3. Lines 1158-1162: One potential result to consider is the EC lesions in Bragin et al 1995. That study reported that entorhinal lesion decreased theta and increased gamma in radiatum. If so, it would be useful either to discuss how that finding fits with the present framework or to clarify why it does not falsify the argument here.**

Long story short: We thank the reviewer for raising Bragin et al. (1995), which represents an important test case for the cascade framework. We have added a new passage to Section 3.7 interpreting this finding in detail.

The expanded logic:

This pre-emptive paragraph may not be necessary, but just in case… We wish to clarify that our framework does not posit theta as a prerequisite for gamma generation. Gamma oscillations emerge from local excitatory-inhibitory network interactions (PING/ING mechanisms) whose resonant frequency is determined by intrinsic membrane properties and synaptic kinetics of the participating interneurons, not by the frequency of the slow oscillation that provides periodic excitatory drive (Buzsáki and Wang, 2012; Bartos et al., 2007). The role of any slower rhythm is simply to deliver temporally structured volleys of excitation that initiate each gamma cycle. Theta happens to be the dominant slow oscillation in the hippocampus during exploration, but gamma couples equally well to delta during NREM sleep (Staresina et al., 2015), to respiration-entrained rhythms at ~2–4 Hz during immobility (Tort et al., 2018; Lockmann et al., 2016; Zhong et al., 2017), and to the neocortical slow oscillation during anesthesia (Isomura et al., 2006). Indeed, Tort et al. (2018) cautioned that respiration-entrained oscillations can overlap in frequency with theta and delta bands, and that previous reports of theta-gamma coupling may in some cases have captured respiration-gamma coupling instead. Therefore, the emergence of rhythmic activity in the CA3-CA1 network after entorhinal lesion in Bragin et al. (1995) is entirely expected from a PING/ING perspective: the local gamma-generating circuitry in CA3 remains intact and, once released from dentate gate suppression, is free to engage with whatever slow periodic input is available. For instance, respiration-coupled oscillations, which are present in the hippocampus independent of theta and entorhinal input (Lockmann et al., 2016) and which have their largest amplitude in the dentate gyrus.

So yes, gamma can increase when theta decreases. The cascade still exists but it not maintained by the usual suspect (theta).

The Bragin et al. (1995) finding is directly predicted by the present framework. After bilateral entorhinal lesion, gamma in the dentate hilus driven by theta modulated feedforward recruitment of interneurons via the perforant path, disappeared. Concurrently, large-the 40-100 Hz activity increased in the CA3 stratum radiatum (fig 9 of Bragin et al., 1995;). Rather than reflecting the "unmasking" of an independent physiological oscillator, we interpret this emergent activity as a pathological consequence of dentate gate failure: with feedforward inhibition removed, disinhibited granule cells deliver unfiltered excitatory drive to CA3 via mossy fibers, and the resulting hypersynchronous activity in CA3's recurrent network manifests as high-power rhythmic activity in the Schaffer collateral termination zone. The decrease in theta power is likewise expected, as the entorhinal cortex is a primary driver of hippocampal theta.

Bragin et al. themselves concluded that CA3-CA1 gamma is normally "suppressed by either the hilar region or the entorhinal cortex." We would restate this as: the entorhinal cortex, through feedforward inhibition, maintains the dentate gate that prevents pathological CA3 activation. Its removal produces exactly the pattern expected from gate failure, loss of organized oscillatory structure and emergence of hypersynchronous activity downstream.

**4. Possible future tests involving septal manipulations: I do not view this as necessary for the current manuscript, but one potentially interesting line of discussion would be to consider datasets in which theta has been manipulated more directly through the septum, for example by inhibiting cholinergic drive, optogenetically pacing theta, or cooling the septum. Predicting what should happen when theta frequency versus theta amplitude is altered could be interesting future steps.**We agree that septal manipulations provide a compelling avenue for future tests of the cascade framework and appreciate the suggestion. We have added a brief mention of this direction in the Conclusions section.

"Septal manipulations offer a particularly direct test of the cascade framework because the medial septum is the primary pacemaker of hippocampal theta. The framework makes specific predictions for two classes of manipulation. Inhibiting septal cholinergic drive, which reduces theta amplitude, should produce proportional broadband reductions in gamma power without selective preservation of individual gamma bands. Graded septal cooling, which progressively slows and weakens theta (Petersen & Buzsáki, 2020), should produce hierarchical spectral degradation consistent with reduced energetic throughput. In each case, the cascade predicts coordinated, proportional changes across the spectrum, whereas independent-oscillator models predict relative preservation of gamma-band structure. We note, however, that forced-frequency paradigms such as optogenetic septal pacing require cautious interpretation, as artificial stimulation can produce field potentials that resemble physiological theta while profoundly disrupting the underlying cellular dynamics (Scarlett et al., 2004)." **Reviewer 2:**

**The authors have clearly invested substantial effort in revising this manuscript, and I appreciate the constructive way in which they engaged with the previous round of feedback. The manuscript has improved in several important respects, particularly in clarity and scope. In this second round, I have aimed to maintain the same level of rigor and depth in evaluating the revised arguments. While some remaining points reflect differences in interpretation, others concern issues that, in my view, require further clarification or revision. The length of this report reflects the extent of the changes introduced in the revision and is intended as a careful and respectful engagement with the authors' work. I leave it to the editors to weigh the necessity of each point for acceptance, but I would like to highlight that I remain particularly concerned about the issues raised in Major Comment 7, as well as the way the "parcellation" framework is characterized throughout the manuscript.**

We thank the reviewer for their continued rigorous and constructive engagement with our manuscript **In particular, I believe the manuscript currently conflates conceptually distinct positions within what is referred to as "spectral parcellation." Several of the cited studies (e.g., Fernández-Ruiz et al., 2023; Lasztóczi & Klausberger, 2016; Lopes-dos-Santos et al., 2018) do not propose that gamma oscillations play a mechanistic role in communication, but rather that band-limited activity can serve as a proxy for pathway engagement. This distinction is critical. In principle, upstream circuits with different temporal structure could impose separable spectral components on downstream LFPs without those oscillations functioning as causal communication channels or entraining downstream spiking. Under this interpretation, gamma-band activity can be used as an indirect readout to test the contribution of specific pathways, rather than as the mechanism mediating communication itself, a point explicitly acknowledged in Fernández-Ruiz et al. (2023). By contrast, stronger claims such as those originally suggested by Colgin et al. (2009) suggest a functional role for gamma in routing or transmission. These are qualitatively different levels of interpretation, and treating them as a single unified "parcellation" position risks misrepresenting the literature. It would strengthen the manuscript to explicitly distinguish between these layers of interpretation before critiquing them.**

We appreciate this important distinction, which motivated a new Section 3.8 (The Separability Assumption and Its Consequences) that explicitly separates proxy from mechanistic interpretations before evaluating each. We note, however, that the three studies cited as examples of the proxy position make claims that extend substantially beyond passive readout, as documented with direct quotations in the new section.

**Regarding 'Major 1': the presentation of the Energy Cascade Model.

As with several other sections, Section 4.1 has been substantially revised. The authors make a clear effort to more precisely articulate the "Energy Cascade Model for Neural Computation," in part by contrasting it with alternative frameworks. Despite this, I found that several aspects of the model remain unclear. Clarifying these points would improve both interpretability for the reader and accessibility for a broader audience.

In the segment stating that phenomena such as "oscillations," "bursts," or "cross-frequency coupling" are not independent signals but structured patterns arising from circuit dynamics, the authors appear to suggest that alternative frameworks treat these phenomena as independent signals exchanged between circuits. It is not clear that this accurately reflects most existing theories, which generally also interpret such activity as emerging from circuit properties, including connectivity, cellular composition, and intrinsic biophysics. The intended distinction may instead be that some frameworks operate at a higher level of abstraction, potentially underemphasizing biophysical constraints or treating frequency bands as functionally separable units. If so, this distinction should be stated more explicitly and justified. Most importantly, how these assumptions arise and in what sense they are intrinsic to those frameworks. As written, the contrast risks being interpreted as a straw-man characterization.**

We thank the reviewer for their continued rigorous engagement with our manuscript.

***Regarding the conflation of conceptually distinct positions within spectral parcellation:***

*We appreciate the conceptual distinction the reviewer draws between gamma as a causal communication mechanism and gamma as a passive proxy for pathway engagement. We agree that these represent different levels of interpretation and have added a new Section 3.8 ('The Separability Assumption and Its Consequences') that explicitly distinguishes between these layers before evaluating each.*

However, we respectfully note that the three studies cited as examples of the proxy position make claims that extend substantially beyond passive readout. Fernández-Ruiz et al. (2023) propose that the interplay between gamma frequency inputs *' determines the precise timing of action potential discharge of CA1 pyramidal cells*,' that gamma-paced spiking in a source region '*potentially entrain target neurons,*' and that theta phase of CA1 firing provides '*a potential mechanism for routing information to different circuits.*' Lasztóczi and Klausberger (2016) conclude that CA1 dynamics are '*structured by rapid, concerted dynamics imposed by converging gamma oscillatory networks.*' Lopes-dos-Santos et al. (2018) hypothesize that theta-nested spectral components reflect '*flexible switching of the hippocampal network between different operating modes, such as memory encoding and retrieval.*' These are functional parcellation claims attributing causal or organizing roles to discrete spectral components, not the use of band-limited activity as a passive readout.

Indeed, the passage the reviewer cites as evidence for the proxy interpretation, that gamma '*does not per se implement any specific function (e.g., attention or memory recall), but rather reports the underlying computations and communication channels for information processing*' replaces named cognitive functions with unnamed ones while retaining the same structural claim: ***that gamma indexes distinct computational operations***. What are these computational operations? Leaving this ambiguous does not excuse that a frequency-function relationship is being made. Unnamed functions are still functions, and claiming gamma indexes them is still a frequency-function assignment regardless of whether the functions are specified.

We therefore maintain that our characterization of these frameworks as spectral parcellation is consistent with the claims advanced in the cited literature. Nevertheless, the new Section 3.8 evaluates both levels of interpretation and demonstrates that our biophysical critique applies to each, since both require spectral separability: the same foundational assumption challenged by the bicoherence, spike-timing, and laminar coherence evidence presented in Sections 3.5–3.7. We further show that even the purely proxy interpretation is redundant with established theta-band laminar methods, circular in its generator assumption, and contradicted by available perturbation data.

**Regarding the straw-man concern:**

The reviewer suggests that parcellation frameworks generally interpret oscillatory activity as emerging from circuit properties, and that our framing risks misrepresenting these frameworks as detached from biophysics. We do not dispute that parcellation papers acknowledge circuit mechanisms. Our critique is not that these frameworks ignore biophysics. It is that they invoke biophysics to generate frequency-specific components and then treat those components as functionally separable entities, assignable to pathways, decodable by downstream targets, and independently manipulable, without demonstrating that the biophysics actually supports such separability. This is not a straw man. It is a specific, empirically testable claim about where the frameworks exceed their biophysical foundations. We have revised the passage in Section 4.1 to make this distinction explicit.

**Regarding the apparent alignment between our position and Fernández-Ruiz et al. (2023):**

We appreciate the comparison but note that the two statements differ in a critical respect. Our claim that 'gamma oscillations mark circuit activation shaped by excitatory-inhibitory balance' means that gamma is a generic consequence of sufficient synaptic drive to local E/I circuits. It does not index which specific pathway is active. By contrast, the claim that gamma '*reports the underlying computations and communication channels for information processing*' treats gamma as a diagnostic readout of specific, separable processes. The first position predicts that gamma will appear similar regardless of which input drives the circuit; the second predicts that different inputs will produce distinguishable gamma components in different layers. These are testable, contrasting predictions, and our bicoherence, ISI, and laminar coherence data support the former.

We note that when the parcellation framework is stripped of independent gamma generators and reduced to its most defensible form, that coordination between upstream inputs and downstream CA1 is organized by theta phase timing and anatomical targeting, the resulting account is not a parcellation model. It is the energy cascade framework described in different vocabulary. Theta organizes when different inputs arrive through phase-dependent timing; anatomy determines where they arrive through laminar targeting. No independent gamma oscillator is required to explain which pathway dominates at a given moment, because theta phase and dendritic layer already specify it. The disagreement is therefore not about whether theta organizes hippocampal dynamics. In this instance, there would be broad agreement. A potential disagreement would exist whether gamma does anything beyond being a spectral consequence of the drive that theta is already organizing.

**My understanding from the revised Section 4.3 is that the cascade model proposes a hierarchical organization across multiple scales, with theta oscillations providing the dominant low-frequency drive. Within this framework, theta defines phases of relatively higher and lower network excitability (e.g., via disinhibition or net excitatory input). During high-excitability phases, increased synaptic drive recruits local excitatory-inhibitory circuit motifs, from which gamma rhythms emerge. In this view, gamma reflects a consequence of local circuit dynamics rather than an independent process. These gamma dynamics, in turn, impose finer-scale structure on neuronal spiking, with higher-frequency components emerging from this modulation, not necessarily as distinct oscillations.**

The reviewer's synthesis is accurate and we appreciate the clarity of this formulation. We note that this understanding, once accepted on its own terms, constrains the interpretations available for the phenomena discussed elsewhere in the review. If gamma reflects the recruitment of local E-I circuits by theta-organized drive, as the reviewer correctly summarizes, then gamma does not preserve information about which upstream pathway sent the drive (as offered in Figure 1 of Fernandez-Ruiz et al., 2023), only how much arrived. The implications for the proxy interpretation are addressed in the new Section 3.8. **Please correct me if this is not an accurate synthesis. If I am missing something, it would be helpful to make this clearer in the text. Assuming this interpretation is broadly correct, it is not clear that the cascade model is mutually exclusive with the views the authors describe as "frequency parcellation." It is well established that driving circuits with excitatory-inhibitory loops using a theta-paced input can generate nested gamma activity, including in canonical network models. Within parcellation frameworks, differences in circuit properties (such as conduction delays, cellular composition, or intrinsic dynamics) could naturally give rise to variation in gamma frequency across circuits under a shared low-frequency drive. In this scenario, gamma would still covary with the strength of the underlying drive. It would therefore be helpful to clarify whether the authors intend to argue that these frameworks are fundamentally incompatible, or instead that there is insufficient empirical evidence for distinct circuits to produce reliably separable, non-overlapping gamma bands.**

The frameworks are empirically incompatible because they make distinct, testable predictions. The cascade predicts continuous spectral shifts with circuit state and proportional broadband changes under perturbation. Parcellation predicts discrete, separable bands amenable to selective elimination. Available perturbation data support the cascade predictions (Zhao et al., 2026). The reviewer asks whether upstream circuits could produce reliably separable gamma bands under shared low-frequency drive. This is an empirical question, and three independent lines of evidence address it directly: bicoherence shows putative slow-gamma components are phase-locked to theta at integer ratios (Sheremet et al., 2019), ISI analysis reveals no distinct gamma-frequency timescale in upstream neurons (Figure 8), and laminar gamma coherence drops sharply at layer boundaries (Berényi et al., 2014). These results indicate that the spectral separability parcellation requires is not present in hippocampal circuits. The full argument, including the demonstration that even the weakest version of parcellation reduces to the cascade framework once independent gamma generators are removed, is developed in the new Section 3.8.

**It would also be helpful to clarify how the framework accounts for the presence of a "mid-gamma" component at the peak of CA1 pyramidal layer theta, a phase typically associated with lower excitability. If gamma reflects the recruitment of local excitatory-inhibitory dynamics by increased drive, one might expect it to be strongest during phases of maximal excitation. While "fast gamma" (if treated as a genuine oscillation rather than a spike-related component) does align with this expectation, it remains unclear how the framework accommodates the coexistence of multiple gamma bands within the same circuit. Several studies (e.g., Schomburg et al.; Lasztóczi & Klausberger), using different analytical approaches, report that fast gamma is largely localized to the pyramidal layer, whereas mid-gamma is strongest in stratum lacunosum-moleculare. It would be useful for the authors to explain how such laminar and spectral dissociations are interpreted within the cascade framework, particularly given that fast gamma is substantially lower amplitude than mid-gamma.**

The framework accounts for the apparent coexistence of multiple gamma bands as a predictable consequence of applying band-limited decomposition methods to non-sinusoidal waveforms that vary in shape across hippocampal laminae (Zhou et al., 2019). The question presupposes the existence of the entities our manuscript argues are methodological artifacts. We address the specific methods below.

ICA's foundational mathematical assumption is statistical independence between components. That is precisely what the energy cascade framework denies. If gamma is hierarchically dependent on theta (if it's a dissipative consequence of theta-driven synaptic currents rather than an independent process) then forcing independence in the decomposition will create artificial separation. Schomburg et al. found a component with maximum spatial loading in radiatum and called it "slow gamma." But ICA finds maximally independent spatial patterns. It doesn't verify that those patterns correspond to independent oscillatory processes. A component concentrated in radiatum could reflect the spatial distribution of theta harmonic structure in that layer. ICA would separate it from the pyramidal layer theta component because the spatial profiles differ, not because the underlying generators are independent. The method cannot distinguish "this is a spatially distinct expression of the same underlying process" from "this is a truly independent oscillatory generator."

CSD is a spatial derivative of the extracellular potential. It localizes where current flows, not what frequency is being generated there. Band-pass filtering followed by CSD shows where current at that frequency enters and exits tissue, but does not establish that the current at that frequency originates from an independent generator at that location.

Neither method actually identifies dominant frequencies in the way that would be needed. CSD gives you current flow, ICA gives you spatial patterns. Neither is a frequency detector. The frequency content attributed to each component or layer is determined after the decomposition, typically through the same band-pass filtering or spectral analysis that Zhou et al. (2019) already showed can mistake harmonics for independent oscillations.

**A clearer contrast between the cascade framework and what the authors term "frequency parcellation" is presented in the paragraph describing gamma as a "spectral front" that shifts with increasing drive. If this is intended as a fundamental distinction, it risks overstating the contrast. In hippocampal contexts, parcellation models can be interpreted more modestly: different upstream circuits may exhibit distinct gamma-band activity due to differences in their network properties. When such inputs converge onto a common target, they could generate synaptic currents with different temporal structure, which would be reflected in the downstream LFP even with no entrainment of downstream spiking activity (see Buzsáki & Schomburg, 2015; Schneider et al., 2021). In this scenario, partially overlapping gamma bands may serve as indicators of pathway engagement without requiring local neurons to be entrained to those rhythms. It would therefore be helpful to clarify whether the authors consider this scenario biophysically implausible, or whether their argument is instead that current empirical evidence does not support it.**

This scenario is addressed in our response to the reviewer's overarching concern about spectral parcellation and in the new Section 3.8. Briefly: the scenario is not implausible in the abstract, but current evidence indicates that the spectral separability it requires does not hold. Upstream neurons lack distinct temporal structure at slow gamma timescales (Figure 8), putative “slow-gamma” components are the consequence of spectral leakage of harmonics due to small time windows (the Gabor-Heisenberg time-frequency trade-off; Zhou et al., 2019), and laminar gamma coherence does not maintain cross-layer relationships (Figure 5).

Please see figure 1 of Fernández-Ruiz et al. (2023):


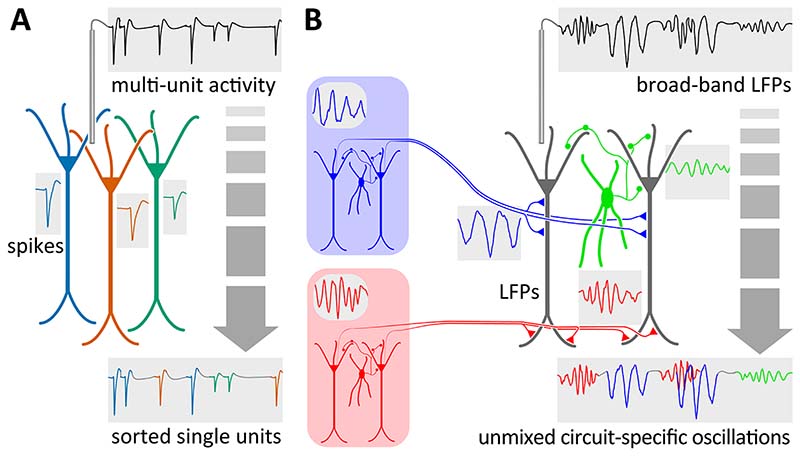


“**A)** Action potentials discharged by nearby neurons are recorded on the same electrode, resulting in an aggregated signal of multi-unit activity (MUA). Spike sorting relies on sampling of the action potentials by an array of recording sites that allow assigning each action potential to an individual neuron (color-coded single-units), notably using spike waveform features. **B)** Likewise, multiple oscillations generated by different rhythm-generating circuits project repetitive volleys of synchronous action potentials via axonal pathways to their downstream targets, producing synaptic current-generating sources that sum up in the extracellular space and give rise to the recorded local field potentials (broad-band LFPs). These individual (color-coded) oscillations can then be demixed and assigned to their generating sources and subcellular domains, notably using their spectro-temporal and spatial characteristics.”

This spike-sorting analogy requires linear superposition. **Independent signals from independent sources combine additively in the extracellular space, and because the mixing is linear, the process is reversible**. One can decompose the mixture back into its components. In the image above, the electrode measuring gamma is in the CA1 pyramidal layer, with a superposition of different gamma oscillations that can be decoupled.

The reviewer is offering one of two positions 1) that gamma components remain confined to their respective dendritic domains and can be read directly from laminar recordings without requiring demixing. The signals never mix. Slow gamma comes into the radiatum and fast gamma into the LM. In this instance, we would need an explanation as to why Fernandez-Ruiz et al. (2023) offer such a demixing research program? The two oscillations should never linearly superimpose. 2) That the oscillations do mix and can be demixed, but do not entrain downstream spiking. This position faces a biophysical contradiction. Dendrites operate as frequency-dependent low-pass filters (Vaidya & Johnston, 2013; Golding et al., 2005), attenuating higher frequencies more strongly than lower ones. A gamma-frequency synaptic current arriving at distal dendrites in stratum lacunosum-moleculare is physically transformed as it propagates toward the soma; attenuated, phase-shifted, and integrated with concurrent currents from other dendritic compartments. The signal that reaches the somatic recording site is not the original input but the product of a nonlinear, frequency-dependent transformation imposed by the dendritic cable itself. Linear demixing methods, whether ICA, spectral decomposition, or any technique based on the assumption that independent sources combine additively, require that the mixing medium is passive and that the original signals are preserved in the mixture. The dendrite is not passive. It actively filters, integrates, and transforms its inputs. Attempting to recover the original upstream gamma signals from a somatic or pyramidal-layer recording is therefore attempting to invert a nonlinear transformation, which is mathematically underdetermined and physically unrealizable. The reviewer's own position, that gamma arrives but does not entrain downstream neurons, confirms that the local circuit has already consumed and transformed the input according to its own dynamics, precisely the condition under which linear demixing fails.

We note that the reviewer's position, that gamma does not entertain downstream spiking, is directly incompatible with the claim it is invoked to defend:

Reviewer: “**When such inputs converge onto a common target, they could generate synaptic currents with different temporal structure, which would be reflected in the downstream LFP even with no entrainment of downstream spiking activity (see Buzsáki & Schomburg, 2015; Schneider et al., 2021).”**

**Fernández-Ruiz et al., (2023):** “The dynamic interplay between both gamma frequency inputs determines the precise timing of action potential discharge of CA1 pyramidal cells”

Does gamma determine spike timing? The reviewer says no. Colgin et al. (2009) says yes. Fernández-Ruiz et al. (2023) say yes, through 'the dynamic interplay between both gamma frequency inputs.' So first, its worth noting that there is a lack of consensus in the literature.

Focusing on Fernández-Ruiz et al. (2023), 'interplay between both' requires simultaneous presence of both components, interacting nonlinearly to shape output. This is incompatible with the cycle-by-cycle alternation proposed by Lopes-dos-Santos et al. (2018), in which individual theta cycles are dominated by one gamma band or the other. The parcellation literature requires both models and can sustain neither.

The reviewer's defense thus concedes the mechanistic claims of the parcellation framework while attempting to preserve the ontological ones. A signal that does not entrain downstream neurons cannot determine their discharge timing, and a signal that has been nonlinearly transformed by dendritic filtering cannot be linearly demixed.

Three claims:

- demixing requires linearity (you know the inputs and can trace them to the inputs; Fernández-Ruiz et al., 2023)
- biology is nonlinear (Vaidya & Johnston, 2013; Golding et al., 2005)
- neurons don't entrain to gamma (Buzsáki & Schomburg, 2015; Schneider et al., 2021).

This forms an impossible triad as any two exclude the third.

We note that the reviewer's suggestion, that gamma-paced synaptic inputs arrive in the downstream LFP without entraining local spiking, constitutes a radical departure from the existing literature. A substantial body of work, including from the groups the reviewer cites in defense of the proxy interpretation, explicitly claims that gamma modulates CA1 spike timing (Fernández-Ruiz et al., 2017; 2023; Lasztóczi & Klausberger, 2014; 2016; Colgin et al., 2009; Lopes-dos-Santos et al., 2018). Moreover, the position reduces to a claim that synaptic input structured at gamma frequencies is visible in the field potential but has no consequence for the neurons generating that field potential. Without an account of why gamma-frequency synaptic input should be treated differently from theta-frequency synaptic input in this regard, given that both produce transmembrane currents in the same dendritic compartments, this argument amounts to: **synaptic input makes neurons fire, but the temporal structure of that input at gamma timescales is irrelevant**. If so, gamma cannot serve as a proxy for anything, because a signal the neurons ignore carries no information about what the circuit is doing.

As described in the revised Section 3.7: while upstream inputs can in principle contribute to downstream field potentials, the premise that upstream populations fire rhythmically at gamma frequencies is not supported by available spike-timing data

**Some parts of the text remain difficult to interpret in concrete terms. For example, the concept of fractality is introduced without a clear explanation of what it corresponds to at the level of neural circuits or cellular organization. The statement that the hippocampal formation exhibits self-similar structural complexity with a fractal dimension of ~2.5 is presented without clarifying what this value implies biologically or functionally. It would be helpful to explain whether this specific value is informative for the proposed framework, and how different values would affect its interpretation. More generally, the role of fractal scaling within the model would benefit from clearer articulation.**

We agree this point needed clarification and have added text explaining the biological significance of fractal scaling for the cascade framework: (Wang et al., 2024). A fractal dimension substantially above 2.0 indicates that neural tissue contains structural complexity across spatial scales (e.g., branching dendrites, nested circuit loops, and hierarchically organized connectivity), providing the physical substrate through which activity can redistribute across temporal scales. The specific value is less important for the cascade framework than the confirmation that the substrate is not smooth or homogeneous but contains structure at every measurable scale.

**The sentence "Metabolic constraints further limit sustained high-frequency activity, while inhibitory feedback and refractory dynamics introduce history-dependent temporal filtering at the circuit level" requires a reference, and it is vague.**We agree this sentence was vague and have revised it with specific mechanisms and supporting references: Sustained high-frequency firing is metabolically costly due to the energetic demands of Na⁺/K⁺-ATPase activity required to restore ionic gradients after each action potential (Attwell & Laughlin, 2001). Inhibitory feedback imposes refractory periods on population activity, and short-term synaptic depression introduces history-dependent gain reduction at excitatory synapses (Zucker & Regehr, 2002), both of which act as temporal filters limiting the propagation of high-frequency structure.

**This claim also needs a reference: "Axonal delays across hippocampal-entorhinal and cortical circuits routinely span 10-50 ms, rendering millisecond-scale oscillations such as gamma (~10-20 ms period) ill-suited for global phase alignment." At least for the hippocampal-entorhinal example.**

We have added supporting references for this claim: Axonal conduction velocities in entorhinal-hippocampal pathways span 0.6–3 m/sec (Andersen et al., 1969; Epson and Heinemann, 1995), and the temporal delays between population activity in successive stages of the EC-hippocampal loop extend to approximately half a theta cycle (~60 ms), substantially exceeding passive conduction time (Mizuseki et al., 2009). These delays render gamma-period timescales (~10–20 ms) ill-suited for precise phase alignment across the full circuit.

**Regarding 'Major 2', New section 4.4:**

**First of all, the authors must revisit how the reference Schomburg et al., (2014) is used in the segment "Regions characterized by strong, punctate synaptic drive, such as CA3 and the dentate gyrus, exhibit greater theta waveform asymmetry and consequently more prominent harmonic power in the 20-50 Hz range. This accounts for the prevalence of 'slow gamma' reports in these regions (Schomburg et al., 2014)...". In the referred study, the authors explicitly note that modulation BELOW ~30 Hz may reflect theta harmonics or waveform asymmetry, and they apply high-pass filtering (>30 Hz) to remove these components. In their own words "We therefore high-pass filtered (>30 Hz) the LFP traces to eliminate the large amplitude theta and its first few harmonics from signals recorded during RUN and REM". Importantly, their analysis treats radiatum associated slow gamma in the 30-80 Hz (not particularly narrow) range as a pathway-specific oscillatory component associated with CA3 input, rather than as a byproduct of theta harmonics. As such, the cited work does not support the interpretation proposed here.**

We agree that our citation of Schomburg et al. (2014) was imprecise and have revised the text. We no longer cite this study as supporting the harmonic interpretation. However, we note that the assumption underlying their filtering approach (that a 30 Hz high-pass filter eliminates theta harmonics) does not hold. Theta at ~8 Hz generates harmonics at integer multiples extending well above 30 Hz (32, 40, 48 Hz, etc.), and our bicoherence analyses demonstrate stable phase coupling between theta and spectral components in this range (Sheremet et al., 2019). A 30 Hz high-pass filter removes only the lowest-order harmonics while leaving the higher-order structure intact. We have revised the manuscript to make this distinction explicit.

**In my view, Prediction 1 lacks discriminative power. As noted above, it is a basic and well-established principle that increasing the level of drive to a network with excitatory-inhibitory loops leads to an increase in the amplitude of the emergent gamma oscillation. In this sense, the prediction does not appear to distinguish the proposed framework from competing models. Unless the authors mean that oscillations above a certain frequency cannot exist without an underlying drive that is itself necessarily oscillatory, this prediction seems limited. At times, I get the impression that the authors may be making this stronger claim, but it is not stated explicitly. If this is indeed the case, it would constitute a strong and testable prediction. Would the authors argue that it is not biophysically possible to generate gamma oscillations in a neural circuit in the absence of an underlying low-frequency rhythm? If so, how does this reconcile with gamma oscillations generated in vitro (e.g., PMC196924; PMID: 9671302)? Would these always be expected to be modulated by theta or another slower rhythm? Similarly, how does the framework account for findings from the primate visual cortex, where persistent gamma oscillations can be driven by visual stimuli (e.g., gratings) without an obvious low-frequency component? Are such oscillations predicted to be necessarily modulated by slower rhythms at the single-trial level? In the hippocampus, the so-called beta2 rhythm (23-30 Hz) (Berke et al., 2008; also reported by Tort's and M.X. Cohen's labs) has been shown to anticorrelate with theta. Although described as "transient" in the original work, it persists for multiple cycles and, during the first minutes of novelty, can exceed theta in amplitude. How does this observation fit within the proposed framework? Would beta2 in this case take the role of the slower organizing rhythm? If so, how flexible is the model with respect to what constitutes the "slow" component**

The reviewer raises an important clarification. The cascade framework does not predict that gamma cannot exist without an oscillatory slow rhythm. It predicts that gamma requires sufficient energetic drive to local E/I circuits. The framework claims gamma reflects how local circuits dissipate energy entering from larger scales. The 'slow' component is defined by its position in the spatial hierarchy, the largest-scale coordinating input available, not by a fixed frequency label. In intact behaving systems, that drive is typically organized by slow oscillations because anatomical constraints favor low-frequency coordination across extended circuits (Buzsáki & Draguhn, 2004). But the drive need not be oscillatory. Tonic excitation, such as pharmacological activation in vitro (Fisahn et al., 1998; Whittington et al., 1995) or sustained sensory input in visual cortex, is sufficient to recruit local E/I dynamics and produce gamma-range activity. The critical variable is the magnitude of synaptic drive, not its temporal structure. We have revised Prediction 1 to state this explicitly.

This resolves the specific cases the reviewer raises. In vitro gamma generated by tonic pharmacological application emerges because sustained chemical drive recruits local E/I dynamics. The cascade prediction is that gamma amplitude should scale with drive magnitude, which it does. Notably, even under tonic pharmacological drive, gamma in hippocampal slices is not spectrally static. It fluctuates in power and frequency, and these fluctuations are structured by whatever low-frequency dynamics emerge from the network (Fisahn et al., 1998). The framework predicts that even in vitro, whatever slow fluctuations are present should show statistical coupling with gamma, a testable prediction that, to our knowledge, remains unexamined. The test is straightforward: extract the amplitude envelope of in vitro gamma and compute its power spectral density. If the cascade framework is correct, the envelope should contain low-frequency structure even under tonic pharmacological drive.

Primate visual cortex gamma under sustained grating stimulation presents a similar case. The visual stimulus provides continuous energy injection. The framework predicts that gamma should be modulated by whatever low-frequency structure is present at the single-trial level, even if it is not visible in trial-averaged analyses. Recent work demonstrating that cortical gamma power is modulated by slow fluctuations in arousal state, microsaccade timing, and attentional state (McGinley et al., 2015) is consistent with this expectation. The apparent absence of a low-frequency component in earlier reports may reflect trial-averaging procedures that wash out non-phase-locked slow dynamics.

That said, the reviewer is correct that Prediction 1 as originally stated was insufficiently specific. The discriminative content is not that gamma covaries with drive; any E-I network predicts that. The discriminative content is the specific form of the covariation: gamma power should scale with the spectral slope and harmonic structure of the low-frequency drive, not simply with its amplitude. Flattening of the spectral slope with increased drive, systematic redistribution of power across frequencies, and correlated decay rates across the spectrum under energy withdrawal are predictions that do not follow from independent-oscillator models. We have revised Prediction 1 to emphasize this structural covariation rather than simple amplitude scaling.

We note that this clarification, taken together with the reviewer's other observations, points toward the resolution the cascade framework provides. If gamma scales with drive, it should not simultaneously carry pathway-specific identity independent of that drive. And if it simply reflects drive magnitude, the decrease in 20–50 Hz power with running speed (Kemere et al., 2013) requires an explanation beyond amplitude scaling, which the entrainment mechanism provides (Zhou et al., 2019). The cascade framework resolves these observations coherently: gamma reflects local dissipation whose spectral expression depends on cross-scale entrainment dynamics, waveform structure, and population-level reorganization, not simply drive magnitude.

Regarding the beta2 rhythm (Berke et al., 2008): several considerations are relevant. First, the 23–30 Hz range falls within the harmonic structure of theta. Second, the novel environments in Berke et al. were characterized by distinct odors and explored through active sniffing, introducing a potential contribution from olfactory beta oscillations generated in the olfactory bulb and piriform cortex (Freeman, 1975; Kay, 2005), which propagate to the hippocampus. No simultaneous olfactory recordings were performed, and Berke et al. did not report controls that would distinguish hippocampally generated beta2 from olfactory-driven input. We do not claim the rhythm is olfactory in origin, but note that this possibility has not been excluded. Third, the observation that a rhythm above theta frequency exceeds theta in amplitude during exploration is unusual in the hippocampal literature and warrants careful methodological scrutiny. We do not dispute the observation, but note that its interpretation as an independent hippocampal rhythm requires ruling out harmonic, olfactory, and methodological contributions, which the original study was not designed to address. If beta2 reflects periodic input from an external source such as respiratory-coupled olfactory drive, it would integrate naturally with the energy cascade framework as an alternative source of slow periodic drive. If it proves to be an intrinsic hippocampal rhythm independent of both theta harmonics and olfactory input, its relationship to the cascade framework (potentially as a transient reorganization of the spectral hierarchy during states of reduced theta dominance) would require further investigation.

**Prediction 2 is stronger. If I understand well, it claims that multiple gamma oscillations cannot exist in a given circuit. Therefore, manipulation experiments cannot get rid of one gamma component in isolation. The sentence "Conversely, CA3 inactivation, predicted by routing models to abolish "slow gamma" (30-50 Hz), produces no significant reduction in any gamma range" needs a reference with experimental evidence.**

We thank the reviewer for noting this omission. We have added the reference (Zhao et al., 2026).

We thank the reviewer for identifying this potential misreading. The cascade framework does not predict the absence of spectral structure at different frequencies. It predicts that such structure arises from hierarchically coupled dynamics rather than independent generators. The testable consequence is that pathway-specific manipulations should produce correlated, broadband spectral changes rather than selective elimination of isolated bands. We have revised Prediction 2 to state this distinction explicitly, clarifying that the prediction concerns interdependence across frequencies rather than the absence of spectral diversity. **Prediction 3. It would be helpful if the authors could clarify why increased asymmetry of the low-frequency component is naturally expected to lead to increased gamma power within the cascade framework (excluding potential contributions from harmonic artifacts). Is the underlying intuition that a more asymmetric theta waveform concentrates synaptic drive into a shorter time window, thereby delivering more energy at once to local excitatory-inhibitory loops? From my understanding, this would be a very elegant prediction.**

The reviewer's intuition is correct. A more asymmetric theta waveform concentrates excitatory drive into a shorter temporal window, delivering more energy per unit time to local E/I circuits. The circuit-level mechanism has been described by Ahmed and Mehta (2009), offering that increased running speed increases excitatory drive from CA3 and medial entorhinal cortex to CA1, elevating interneuron firing rates and shifting gamma frequency upward, consistent with models in which gamma frequency scales with excitatory drive to interneuron networks (Traub et al., 1996).

This mechanism connects directly to the bicoherence framework. The waveform asymmetry parameter, the biphase φ in the Abreu formulation, controls how energy is distributed across harmonics. Greater asymmetry redistributes energy from the fundamental into higher-order components, including the gamma range. The prediction is therefore quantitative: theta-gamma coupling strength should scale with theta waveform asymmetry, measurable through the biphase. This is confirmed in our data (Sheremet et al., 2019). We have added a brief clarification to Prediction 3 citing this mechanism.

**Prediction 4 would benefit from further clarification. The statement that models positing independent gamma channels predict relative preservation of gamma structure under such manipulations is difficult to interpret in concrete terms. For example, in the canonical CA3-EC-CA1 system, where theta is present across regions, what would it mean experimentally to "disrupt theta"? More generally, it is not clear that this prediction cleanly distinguishes the proposed framework from alternative models. Even in frequency parcellation accounts, gamma activity depends on synaptic drive and local excitatory-inhibitory dynamics, so perturbations of low-frequency input would also be expected to affect gamma. It would therefore be particularly helpful for the authors to describe a concrete experimental scenario (e.g., replacing rhythmic input with tonic drive in a defined pathway) and to specify the distinct outcomes predicted by each framework.**

We agree that a concrete experimental scenario strengthens this prediction. Consider optogenetic silencing of the medial septum to abolish theta while leaving CA3 and entorhinal circuits intact.

The cascade predicts: gamma power should degrade proportionally across the entire broadband spectrum, the spectral slope should steepen, and no individual gamma band should be selectively preserved because gamma depends on the large-scale drive that theta organizes, not on independent pathway-specific generators.

Parcellation predicts: if slow gamma and mid-gamma arise from independent generators in CA3 and EC respectively, their spectral signatures should persist in the downstream LFP (perhaps reduced in amplitude) even when theta-phase organization is removed, because the generators themselves remain intact.

The reviewer's suggestion, replacing rhythmic input with tonic drive, yields a similar contrast. The cascade predicts that tonic drive should produce gamma-range activity that under the cascade framework, produce broadband increases across all frequencies without the emergence of discrete pathway-specific bands. Parcellation predicts that specific gamma bands should persist because they arise from pathway-specific circuit properties rather than from the temporal structure of the drive.

Existing data support the cascade prediction: systematic energy reduction via barbiturate overdose produces hierarchical spectral degradation in which high-frequency components collapse before low-frequency rhythms, with correlated decay rates across the spectrum (Zhou et al., 2021). We have revised Prediction 4 to include this concrete scenario.

**Regarding Major 3, I appreciate the authors' discussion of the PV⁺ and SST⁺ manipulation study. However, the first part of my comment was not addressed; specifically, the possibility that two upstream circuits converging onto the same downstream target may exhibit partially distinct gamma activity, such that their synaptic inputs could contribute separable components to the downstream LFP. This alone may be sufficient to justify the use of gamma-band activity as a proxy for pathway engagement. In the hippocampus, for example, gamma components resembling those observed in upstream regions (e.g., EC and CA3) appear to align with their respective dendritic targets in CA1 (stratum lacunosum-moleculare and stratum radiatum), and occur at corresponding theta phases associated with activity in those inputs. It would be helpful for the authors to clarify how this possibility is accounted for within the proposed model: if upstream populations exhibit distinct gamma-band activity, their synaptic inputs to a shared downstream target would be expected to be temporally structured at those respective frequencies, potentially giving rise to multiple gamma components in the resulting LFP when these inputs are not spectrally identical.
In this section of the rebuttal letter, the authors again cite Schomburg et al. (2014) in a way that is difficult to reconcile with the original study. The statement that activity in t30-50 Hz ranges may reflect theta harmonics is not supported by that work. Schomburg et al. explicitly note that components below ~30 Hz may be contaminated by theta harmonics and apply high-pass filtering (>30 Hz) to remove these contributions. Their subsequent analyses treat activity above 30 Hz as reflecting a distinct oscillatory component. As such, using this reference to support the interpretation that 30-50 Hz activity reflects theta harmonics is problematic and should be revised or more carefully qualified. I comment more on the harmonics issue in my comments on Major 7 below.**

We addressed the proxy argument in detail in our response to the reviewer's overarching concern about spectral parcellation (see above). To briefly restate: the claim that upstream circuits 'exhibit partially distinct gamma activity' whose synaptic inputs 'contribute separable components to the downstream LFP' assumes the existence of the upstream generators whose existence is the question at issue. Our ISI analysis (Figure 8) directly tests whether CA3 and MEC neurons exhibit temporal structure at slow-gamma timescales. They do not. Without evidence that upstream populations generate distinct gamma-band activity, the expectation that their inputs would be 'temporally structured at those respective frequencies' lacks empirical foundation. We refer the reviewer to our extended discussion in the parcellation response, section 3.8, which addresses this argument in full.

Regarding the laminar alignment of gamma components with dendritic targets: we agree that CA3 projects to radiatum and EC projects to lacunosum-moleculare. Anatomy constrains where current flows. It does not determine the frequency at which the current oscillates. As detailed in Section 3.7, a non-sinusoidal theta waveform varying in shape across laminae will produce different harmonic CSD profiles at different depths. The appearance of spectrally distinct components in different layers is a predictable consequence of laminar variation in waveform geometry, not evidence for independent pathway-specific generators.

The Schomburg citation has been corrected as noted.

“We therefore high-pass filtered (>30 Hz) the LFP traces to eliminate the large amplitude *theta and its* ***first few harmonics*** from signals recorded during RUN and REM.”

We also note that the Schomburg et al. supplemental methods describe the 30 Hz high-pass filter as removing '*the large amplitude theta and its first few harmonics*.' This language indicates partial removal of the lowest-order harmonics, not a complete separation of harmonic from non-harmonic structure. The 4th and higher harmonics of an 8 Hz theta (32, 40, 48 Hz) pass through this filter and remain in the analyzed signal. We do not dispute that Schomburg et al. treated activity above 30 Hz as a distinct component. We note that the filtering procedure they employed does not establish that it is a distinct component. **Regarding Major 4, I find the authors' emphasis on the lack of mechanistic explanation to be a strong and valuable point. The literature in this area is often largely descriptive, and statements such as "any more than observing theta in multiple regions implies multiple types of theta oscillation" capture this issue particularly well. I would have welcomed a stronger focus on this line of argument, although I understand the authors' preferred to focus on other arguments. In contrast, I'm still not convinced by the utility of the associated figure. As noted previously, simply illustrating variability in how gamma bands are defined does not substantially advance the mechanistic critique. In addition, at least one citation issue remains unresolved (as I wrote before: "Of note, Lopes et al. define fast gamma largely above 100 Hz (tSC5), rather than around 80 Hz as implied in your Figure 1"); however this is relatively minor compared to other citation concerns raised elsewhere.**

The reviewers original comment was:

“Major 4. I did not find the arguments presented in Figure 1 and the associated text particularly convincing. The authors suggest that variability in reported gamma-band definitions across studies reflects a tendency for other groups to flexibly adjust frequency boundaries to fit their theoretical interpretations. However, this variability seems more likely to reflect the lack of a single, objective way to define gamma bands across studies.
In practice, the cited papers use quite different analytical approaches. For example, Fernandez-Ruiz et al. (2017) seem to visually inspect power spectra from ICA-extracted components in multichannel recordings; Lasztoczi & Klausberger define gamma bands based on CSD signals decomposed as amplitude modulations across theta phase (also visual inspection it seems); Scheffer-Teixeira & Tort focus on modulation index (MI) plots; and Lopes-dos-Santos et al. (2018) primarily report peak frequencies of spectrogram IC components rather than frequency bands. Of note, Lopes et al. define fast gamma largely above 100 Hz (tSC5), rather than around 80 Hz as implied in your Figure 1.
At the same time, some features are fairly consistent across studies. For example, several reports place "mid" gamma near the theta peak and associate it with LM. Much of the apparent variability instead seems to arise from loosely defined bands, often based on visual inspection of data processed in different ways, and in some cases it is not even clear how band boundaries are determined. I agree this is a real problem. However, the figure risks overstating how arbitrarily or strategically band boundaries are chosen in order to make these theories appear less falsifiable. I may be missing something here, and if so I'd welcome clarification from the authors.
Relatedly, when discussing Fernández-Ruiz et al. (2021), the authors note that slow gamma is associated with routing object-related information and present this as a contradiction to earlier theories. However, I could not find any claim in that work that this "slow gamma" refers to the same slow gamma proposed by Colgin and others in the context of CA3-CA1 interactions. Rather, Fernández-Ruiz et al. analyze a rhythm linked to LEC-DG communication. This reflects a lack of clarity in how oscillations are labeled, rather than a genuine contradiction of Colgin's framework.”

The reviewer’s central argument was that the variability reflects "*the lack of a single, objective way to define gamma bands across studies*" and that "*the cited papers use quite different analytical approaches.*" The reviewer then listed ICA, CSD amplitude modulation, modulation index plots, and spectrogram IC components as examples of this methodological diversity. So, the reviewer’s position can be distilled as “*the variability is innocent, it's just different methods.*”

This is precisely what we have been arguing throughout this entire exchange. Different methods produce different apparent gamma bands because each method handles theta harmonics, non-sinusoidal waveforms, and time-frequency tradeoffs differently. Zhou et al. (2019) demonstrated this explicitly by showing that wavelet and EEMD produce "slow gamma" in synthetic data containing only theta and its harmonics. The definitional variability across labs isn't despite good methods. It's because of method-dependent artifacts.

We have corrected the frequency range attributed to Lopes-dos-Santos et al. in Figure 2. Lopes dos santos says: "Peak frequencies across all mouse recording days (median and interquartile range) are as follows: tSC1, 22 Hz, 21–23 Hz; tSC2, 35 Hz, 34–36 Hz; tSC3, 54 Hz, 52–55 Hz; tSC4, 80 Hz, 77–82 Hz; tSC5, 169 Hz, 153–174 Hz." We moved the range to tSC5 per the reviewers request and then placed the other bands as well.

We respectfully maintain the figure's utility, however, because the very issue the reviewer identifies illustrates its point: Lopes-dos-Santos et al. define fast gamma as >100 Hz, whereas Colgin et al. (2009) define it as 65–140 Hz. If these labels referred to discrete biological entities with consistent mechanisms of generation, independent laboratories would converge on common frequency boundaries. The persistent failure to converge is evidence that these categories may reflect analysis choices imposed on continuous spectral structure rather than natural kinds.

Indeed, the absence of a mechanistic explanation for how these putative oscillations are generated is what permits frequency definitions to drift across studies without the inconsistency becoming apparent. A mechanistically defined entity, anchored to specific circuit properties, synaptic time constants, or interneuron populations, would predict a convergent frequency range. The persistent definitional variability suggests that the labels are accommodating correlational findings rather than tracking a stable biological process.

The variability isn't just 'labs disagree, therefore maybe the thing isn't real'" is conversational. Consider: "The definitional variability is not merely an inconvenience of cross-laboratory comparison. It follows the pattern predicted by method-dependent artifacts: different decomposition methods applied to the same non-sinusoidal waveform produce different apparent band boundaries. When tested with a method insensitive to these artifacts, direct examination of spike timing (Figure 8), the putative oscillatory entity is absent. **Regarding Major 5, point 1 ("Anatomy constrains location, not frequency") is well written, and I have no further comments.**

Thank you.

**Point 2 requires correction. I agree that CSD analyses can be problematic when a specific channel is used as the reference for triggering LFP averaging before computing CSD. However, none of the studies I cited (Schomburg et al. (2014) or Lasztóczi & Klausberger (2016)) used that procedure. Schomburg et al. used ICA to decompose multivariate silicon probe recordings and identified the slow-gamma component from the component with strongest loadings in radiatum. Lasztóczi & Klausberger (2016) computed CSD directly from multichannel LFP recordings and then derived gamma components from those data. In other words, the artifact you describe does not apply here.**

We note that our prior rebuttal described the reference-dependent artifact as a general concern for CSD-based inferences, not as a specific critique of the methods used by Schomburg et al. or Lasztóczi & Klausberger. We agree that ICA decomposition and direct CSD computation do not suffer from the specific triggering artifact described. The manuscript cites Csicsvari et al. (2003) for this point independently.

That said, neither ICA nor CSD is a frequency detection/decomposition method. ICA identifies spatially independent components. CSD identifies where current flows. Neither establishes what frequency the underlying generator oscillates at. In both cases, the frequency content attributed to each component or layer is determined after the decomposition, typically through band-pass filtering or spectral analysis, the same methods Zhou et al. (2019) demonstrated can produce the appearance of separable gamma components from signals containing only theta and its harmonics. Schomburg et al.'s ICA component with strongest loadings in radiatum may reflect the spatial distribution of theta harmonic structure in that layer rather than an independent slow-gamma generator. ICA would separate it from the pyramidal layer theta component because their spatial profiles differ, not because their underlying generators are independent. The method cannot distinguish 'this is a spatially distinct expression of the same underlying process' from 'this is a truly independent oscillatory generator.'

**Point 3 appears to diverge from the interpretations proposed by Buzsáki & Schomburg (2015) and Schneider et al. (2021). This is entirely reasonable and could represent a valuable contribution, but it should be stated explicitly. Both studies argue that gamma-paced synaptic inputs from upstream circuits can be reflected in downstream LFPs, and therefore caution against interpreting LFP-LFP or spike-LFP coherence as direct evidence of effective communication. If the authors instead argue that observing oscillatory structure in a downstream region requires engagement of local circuit dynamics, this constitutes a clear departure from those models and should be explicitly framed as such.**

The reviewer is correct that our interpretation constitutes a departure from the models proposed by Buzsáki & Schomburg (2015) and Schneider et al. (2021), and we appreciate the suggestion to state this explicitly. Both studies argue that upstream gamma-paced synaptic inputs can be passively reflected in downstream LFPs. Our framework goes further: we propose that gamma-range spectral structure in the downstream LFP primarily reflects the impedance properties and E/I dynamics of the local receiving circuit responding to afferent drive, rather than a faithful transmission of upstream temporal structure. We have added a sentence to Section 3.7 making this departure explicit **Regarding Major 6, I do not find the argument based on low coherence values compelling. Measured coherence is strongly influenced by signal-to-noise ratio, and theta being a slower, more global signal is likely to have a higher SNR than gamma, which is faster and more spatially localized. Further, absolute coherence values in the range of ~0.2-0.25 being labeled as "low" is somewhat subjective. Moreover, neuronal firing is determined by multiple factors, and gamma coherence would not be expected to account for all of them. That said, I recognize that this line of argument is considered persuasive by many in the field, and I raise these points primarily to encourage scientific discussion rather than as a central objection.**

We appreciate the reviewer raising this for discussion. We note that coherence values of 0.2–0.25 correspond to ~20% shared variance between regions. Whether this is labeled 'low' is less important than what it implies for the proposed mechanism: a communication channel that accounts for ~20% of the variance between sender and receiver faces a substantial explanatory burden. The reviewer's point about SNR is well taken, but we note that if gamma coherence is low precisely because gamma is weak, spatially localized, and non-stationary, these are intrinsic properties of the signal (not measurement artifacts) and they constrain gamma's plausibility as an inter-regional coordination mechanism. And if the signal-to-noise is low for the researcher, then what’s the plausible mechanism of influence in the biology? Indeed, the reviewer's own explanation, that theta produces high coherence because it is a slower, more global signal with higher SNR, articulates precisely why the cascade framework identifies theta, not gamma, as the primary inter-regional coordinating rhythm.

We have now addressed this in the manuscript.

**Regarding the authors' item 2, I agree with what the authors outline but it remains unclear to me how perturbation of entorhinal cortex activity SELECTIVELY affects mid-gamma in CA1 stratum lacunosum-moleculare, while leaving fast gamma in the CA1 pyramidal layer and slow gamma in CA1 radiatum relatively unaffected.**

The premise of this question is directly addressed by available perturbation data. Entorhinal inactivation does not selectively affect fast/mid-gamma while leaving other bands unaffected. Zhao et al. (2026) demonstrated that entorhinal silencing produces broadband spectral reduction across frequencies, including the 20–50 Hz range, rather than selective elimination of a mid-gamma band. This is the outcome predicted by the cascade framework (Prediction 2) and is inconsistent with selective pathway-frequency correspondence. **Regarding Major 7, this is the section where I have the most difficulty with the authors' rebuttal. I agree with the central point that energy in a frequency band does not, by itself, demonstrate the existence of an independent oscillator, and that asymmetric theta waveforms naturally generate harmonics. I also appreciate the addition of the analysis in Figure 8. In particular, the absence of slow-gamma structure in spike timing is an important result. It would strengthen the manuscript to frame this more explicitly as a falsification-relevant test: namely, that the presence of a consistent slow-gamma timescale in ISIs would constitute credible evidence for a generator, but we do not observe it. At present, this result is presented more as supportive evidence for the harmonics interpretation rather than as a clear criterion that could, in principle, have supported the opposing view. Making this explicit would clarify that the authors' position remains open to counter-evidence, but that such evidence is not found in the data.**

We thank the reviewer for this suggestion and agree that the epistemological framing matters. The revised caption for Figure 8 now explicitly states, for each panel, what observations would constitute credible evidence for an independent slow-gamma generator, a consistent attractor in the 20–50 ms ISI range, a separable modulation peak in the 25–50 Hz band, or rhythmic structure in autocorrelograms at slow-gamma timescales, before reporting that no such evidence is found. This framing clarifies that our position remains open to counter-evidence and that the analysis constitutes a survived falsification test rather than purely confirmatory support. **I find the bicoherence-based argument less compelling, as it relies on more complex analytical assumptions. For example, methods such as EMD have been proposed to separate nonlinear waveform components and mitigate harmonic artefacts, but there is no clear mathematical guarantee that they are fully free from such confounds as far as I know. A similar concern applies to bicoherence-based approaches. For this reason, I would encourage the authors to emphasize simpler, more mechanistically interpretable tests where possible. In that context, one suggestion from my previous review was ignored: a direct comparison of harmonic power (e.g., testing whether putative slow-gamma components exceed the power of lower-order theta harmonics at 16 and 24 Hz). If slow gamma reflects a higher-order harmonic, it would not be expected to carry more energy than preceding harmonics. Would the authors agree that this provides a straightforward and complementary test? If not please let me know why for the sake of my own curiosity. Anyway, including such simple, interpretable analyses could further strengthen the argument.**

As this comment has been highlighted as among the most important, we will take care to parcel the statements and address the reviewer’s concerns systematically.

1. **Bicoherence Does Not Rely on Complex Analytical Assumptions**

*“I find the bicoherence-based argument less compelling, as it relies on more complex analytical assumptions.”* The reviewer successfully used the parameters of bicoherence when they employed the Abreu simulation elsewhere. Therefore, they must find that the parameters defined by bicoherence are somewhat simple to use and understand.

Bicoherence is one of the more mathematically transparent higher-order spectral tools. It is a **direct** extension of Fourier analysis. The bispectrum measures whether three frequency components (f₁, f₂, and f₁+f₂) maintain a stable phase relationship. Bicoherence is the normalized version, giving a value between 0 and 1. The underlying math is the same Fourier basis used for any power spectral analysis. It examines third-order statistics (phase coupling between triplets) instead of second-order statistics (power). *The assumptions are essentially stationarity over the analysis window and sufficient data length. These are the same assumptions Fourier analysis itself requires.*

Compare that to EMD/EEMD, which genuinely does rely on complex, poorly constrained assumptions (adaptive basis functions, no fixed mathematical framework, known mode-mixing problems, and no clear error theory). Grouping bicoherence with EMD as though they share similar analytical vulnerabilities is a category error. They are fundamentally different kinds of tools. Bicoherence has decades of rigorous mathematical development in physics and engineering, particularly in wave dynamics, where it was specifically designed to distinguish harmonically coupled components from independent oscillations, ***which is exactly the question at issue here.***

**1.1 Proof: Bicoherence Is Derived Directly from the Fourier Decomposition**

The assumptions presented by the reviewer appear to stem from a lack of familiarity with bicoherence, which is a simple extension of the Fourier decomposition. If the reviewer accepts any power spectral analysis based on Fourier decomposition, they have implicitly accepted every assumption that bicoherence requires. The window size choice, the stationarity requirement, the frequency resolution – **these are all identical**. The statement that bicoherence “*relies on more complex analytical assumptions*” lacks a comparison (more complex relative to what?).

The Fourier decomposition of a windowed signal segment produces a complex coefficient $X\left( f \right)= A\left( f \right)\cdot e^{i\varphi\left( f \right)}$ at each frequency f, containing both an amplitude A(f) and a phase φ(f).

The power spectrum computes |X(f)|², retaining the amplitude while discarding the phase spectrum.

Bicoherence uses the same Fourier coefficients, including the phase information, to test whether the phase relationship among frequency triads (f₁, f₂, f₁+f₂) is consistent across segments. Formally, one computes the average of X(f₁)·X(f₂)·X*(f₁+f₂) across segments and normalizes. The analytical framework is identical to power spectral estimation; the sole difference is that bicoherence does not discard the phase information already contained in the Fourier coefficients.

**1.2 Precedent: Bicoherence in Acoustics and Signal Processing**

This approach is not novel or exotic. In vocal analysis, bicoherence has been used for decades to distinguish harmonics of vocal fold vibration that arise from nonlinear coupling from independently occurring spectral components (Fackrell & McLaughlin, 1996). In musical acoustics, bicoherence differentiates instrument families by their phase-coupling structure: trumpet harmonics show strong quadratic phase coupling while flute harmonics do not, despite overlapping power spectra (Dubnov & Rodet, 2003). The method has been applied to synthetic speech detection (AlBadawy et al., 2019), Alzheimer’s disease classification from voice recordings (Nasrolahzadeh et al., 2018), and emotion recognition from speech (Yogesh et al., 2017). In each case, the power spectrum alone cannot determine whether a spectral component at f₁+f₂ arises from nonlinear interaction or exists independently; bicoherence directly addresses this. The present application asks the same question of hippocampal oscillations.

Therefore, as the assumptions are no more or less than those of Fourier, unless the reviewer wishes to contest that Fourier has complex analytical assumptions.

**2. The Reviewer’s Own Simulation Is Parameterized by Bicoherence Variables**

**2.1 The Abreu et al. (2010) Formulation**

The Abreu et al. (2010) formulation that the reviewer employed is parameterized by r (the nonlinearity index, controlling the distribution of energy across harmonics) and φ (the waveform parameter). Abreu et al. explicitly identify φ as the biphase, citing Elgar and Guza (1985), *the same quantity that bicoherence measures across analysis windows*.

The reviewer’s simulation, which they report running successfully, is parameterized by bicoherence variables. **In brief,** the Abreu et al. (2010) formulation the reviewer employed below is parameterized by the biphase (φ) and the nonlinearity index (r), the same quantities that bicoherence measures in empirical data. The reviewer’s finding that the spectral outcome depends on which value of φ is selected underscores, rather than undermines, the importance of measuring this quantity in actual hippocampal recordings. **The reviewer successfully discovered how altering bicoherence parameters in the Abreu model changes waveform shape, but characterizes the quantification of those same parameters as complex.**

**2.2 The Reviewer’s Abreu Simulation**

*“I simulated myself asymmetric theta waveforms (Abreu et al., 2010). In this framework, increasing asymmetry via faster rise times (consistent with hippocampal theta) redistributes energy into higher harmonics and, when total energy is controlled, tends to increase rather than decrease power in the 20–50 Hz range.”*

The Abreu waveform (their Eq. 6) is:

$$u\left( t \right)=U_{w}\Sigma_{\left\{ k=0 \right\}}^{\left\{ \infty\right\}} \left( \frac{1}{n^{k}} \right)\sin\left[ \left( k+1 \right)\omega t + k\varphi\right]$$

So the Fourier component at the m-th harmonic (frequency $mf^{0}$) has amplitude proportional to$\frac{1}{n^{\left\{ m-1 \right\}}}$and phase equal to $\left( m-1 \right)\varphi$.

**2.3 Proof: The Abreu Parameter φ Is the Biphase**

Compute the biphase for any triad. Take the general case: harmonics m, n, and m+n. The biphase is:

$$\psi= phase(mf₀) + phase(nf₀) - phase((m+n)f₀)$$

$$\psi= \left( m-1 \right)\varphi+ \left( n-1 \right)\varphi- \left( m+n-1 \right)\varphi$$

$$\psi= \left( m - 1 + n - 1 - m - n + 1 \right)\varphi$$

$$\boldsymbol{\psi= -\varphi}$$

**The biphase is −φ for every single triad.** Not just the fundamental self-interaction. Every harmonic pair in the entire waveform. The Abreu parameter φ ***is*** the biphase. Not analogous to, not related to, not “connected to.” It is the biphase, with a sign convention.

**The reviewer used the variables that bicoherence measures to make an asymmetric waveform with harmonics, but debates its utility in measuring harmonics?**

**2.4 Parameter Mapping**

| **Abreu Parameter** | **Bicoherence Measure** | **What It Controls** |
| --- | --- | --- |
| φ (biphase) | Biphase ψ(f₁,f₂) = −φ | Phase relationship between harmonics |
| r (nonlinearity) | Bicoherence magnitude pattern | Energy distribution across harmonics |
| Both together | Full bispectrum | Complete third-order spectral structure |

When the parameters that bicoherence measures, the biphase and the nonlinearity index, are varied directly, as in the reviewer’s Abreu simulation, waveform shape and harmonic structure in the power spectrum change accordingly. The reverse also holds: to quantify how waveform shape and harmonic structure in the power spectrum are interdependent, one uses bicoherence.

The question is whether spectral energy in the 20–50 Hz range reflects theta harmonics or an independent oscillation. **The reviewer’s own simulation demonstrates that the answer depends on the biphase and the nonlinearity index - the parameters that control waveform shape and harmonic structure in the Abreu et al. (2010) formulation. If one wanted to determine this from empirical data, one would measure exactly those parameters. That is bicoherence.** The reviewer’s simulation successfully demonstrates both that these parameters are neither analytically complex nor difficult to interpret, and that they are necessary for resolving the question at hand. This is why we use bicoherence.

**3. EMD Is the Wrong Tool for This Question**

*“For example, methods such as EMD have been proposed to separate nonlinear waveform components and mitigate harmonic artefacts, but there is no clear mathematical guarantee that they are fully free from such confounds as far as I know.”*

**3.1 EMD Addresses a Different Question**

The reviewer suggests that EMD has been proposed to “separate nonlinear waveform components and mitigate harmonic artefacts.” This mischaracterizes the claims of EMD’s developers. Huang et al. (1998) argued that Fourier analysis distributes the energy of a non-sinusoidal waveform across multiple harmonic components, and that EMD avoids this by representing non-sinusoidal oscillations within single intrinsic mode functions using adaptive, non-sinusoidal basis functions (Wu & Huang, 2004). This is a legitimate optimization for questions about waveform morphology. However, it is the wrong optimization for the question at hand.

Our analysis does not ask “what does the theta waveform look like?” It asks “is spectral energy in the 20–50 Hz range generated by nonlinear coupling to theta, or does it arise from an independent oscillatory process?” This is a question about the relationship between frequency components, not about waveform shape. EMD, by design, attempts to collapse harmonics into a single waveform component, eliminating precisely the inter-frequency phase information needed to answer this question. Bicoherence retains that information and tests it directly: if spectral energy at f₁+f₂ is phase-locked to energy at f₁ and f₂ across analysis windows, it was generated through nonlinear interaction. If the 20–50 Hz components were independent of theta, as the “slow gamma” interpretation requires, bicoherence at the corresponding bifrequency pairs would not differ from zero. It does. No variant of EMD provides this test.

**3.2 EMD’s Limitations Are More Severe Than the Reviewer Acknowledges**

The reviewer is correct that EMD has “no clear mathematical guarantee” against harmonic confounds. This substantially understates EMD’s limitations. Huang and Wu (2008) acknowledged that the theoretical basis of EMD is “purely empirical” and identified mode mixing, in which energy from different physical processes is unpredictably distributed across intrinsic mode functions, as “one of the main drawbacks of EMD.” The decomposition is not guaranteed to produce the same IMFs for the same signal across runs. The method’s developers and subsequent investigators have cataloged a series of unresolved problems including end effects, spline interpolation sensitivity, and IMF non-uniqueness (Datig & Schlurmann, 2004; Chen & Feng, 2003), each addressed by heuristic patches that introduce additional free parameters. The ensemble variant (EEMD; Wu & Huang, 2009) adds noise amplitude and ensemble size. The masking variant adds masking signal frequency and amplitude. Endpoint handling methods range from mirror extension to autoregressive models to neural network prediction, each with their own parameter choices. None of these parameters are constrained by formal principles.

**3.3 Direct Comparison of Free Parameters**

Bicoherence requires one free parameter (window length), governed by the Heisenberg–Gabor uncertainty principle, with a known null distribution and formal significance test. EMD requires, at minimum, selection of a sifting stopping criterion and interpolation method, and in practice - if one uses any variant that addresses mode mixing - noise amplitude, ensemble size, or masking signal parameters, none governed by formal principles, with no null distribution and no significance test. We leave it to the reviewer to determine which method relies on “more complex analytical assumptions.”

**4. “A Similar Concern Applies to Bicoherence”**

*“A similar concern applies to bicoherence-based approaches.”*

We are unclear what concern is intended. The limitation the reviewer identified for EMD, that it cannot guarantee correct separation of harmonic components, is a concern specific to decomposition methods, because they attempt to assign spectral energy to discrete components.

Bicoherence does not decompose, separate, or assign anything. It evaluates a statistical quantity [the consistency of the phase relationship among frequency triads across analysis windows (**the same variables the reviewer changed in the Abreu model**)] against a formal significance threshold. If the concern is that bicoherence might detect spurious phase coupling, we note that this is addressed by the well-characterized null distribution of the bicoherence statistic (Elgar & Guza, 1988), by the complementary biphase variance test (Sebert & Elgar, 1989), and by the Hinich (1982) test for distinguishing nonlinear coupling from non-Gaussian linearity. These are formal statistical safeguards with no analogue in the EMD framework.

**5. Power Comparison Is Not a Valid Test of Harmonics**

*“For this reason, I would encourage the authors to emphasize simpler, more mechanistically interpretable tests where possible. In that context, one suggestion from my previous review was ignored: a direct comparison of harmonic power (e.g., testing whether putative slow-gamma components exceed the power of lower-order theta harmonics at 16 and 24 Hz).”*

**5.1 Arc of the Reviewer’s Argument**

It is worth pausing to trace the arc of the reviewer’s argument across this comment:

- The reviewer rejected bicoherence, the test that directly answers “are these harmonics?,” for being too complex, despite using the same parameters in their simulation.
- They proposed EMD, which does not answer the question at all, as an alternative.
- They now propose comparing power across frequency bins as a test of whether something is a harmonic. Note well, ***the definition of a harmonic is an integer, phase-locked relationship. Power does not define a harmonic.***

**5.2 Why Power Cannot Adjudicate This Question**

**Harmonics are not defined by power. They are integer phase-coupled relationships.** Relative power across frequency bins does not distinguish harmonics from independent oscillations. A harmonic series produced by nonlinear coupling can exhibit any power distribution across harmonic orders, depending on the strength and form of the nonlinearity. Equally, independent oscillations at those frequencies can have any relative power. The two hypotheses, A) that 20–50 Hz energy reflects theta harmonics versus B) an autonomous “slow gamma” oscillation, make no differential prediction about relative spectral power. The proposed test therefore has no discriminative capacity for the question being asked.

Bicoherence, by contrast, yields a direct, binary prediction. If 20–50 Hz energy is generated through nonlinear coupling to theta, bicoherence at the corresponding bifrequency pairs (f₀, f₀), (2f₀, f₀), etc., will be significantly non-zero. If it arises from an independent oscillatory process, bicoherence at those bifrequencies will not differ from zero. This is evaluated against a formal significance threshold with a known null distribution. We respectfully suggest that a test yielding a yes-or-no answer to the specific question at issue, grounded in the same Fourier coefficients the field already relies upon, is both simpler and more mechanistically interpretable than a power comparison that is ambiguous with respect to both hypotheses.

**6. Why We Did Not Include the Suggested Power Analysis**

*“In that context, one suggestion from my previous review was ignored: a direct comparison of harmonic power (e.g., testing whether putative slow-gamma components exceed the power of lower-order theta harmonics at 16 and 24 Hz). If slow gamma reflects a higher-order harmonic, it would not be expected to carry more energy than preceding harmonics. Would the authors agree that this provides a straightforward and complementary test? If not please let me know why for the sake of my own curiosity. Anyway, including such simple, interpretable analyses could further strengthen the argument.”*

**6.1 Empirical Data**

We present the following figure from Sheremet et al. (2020):


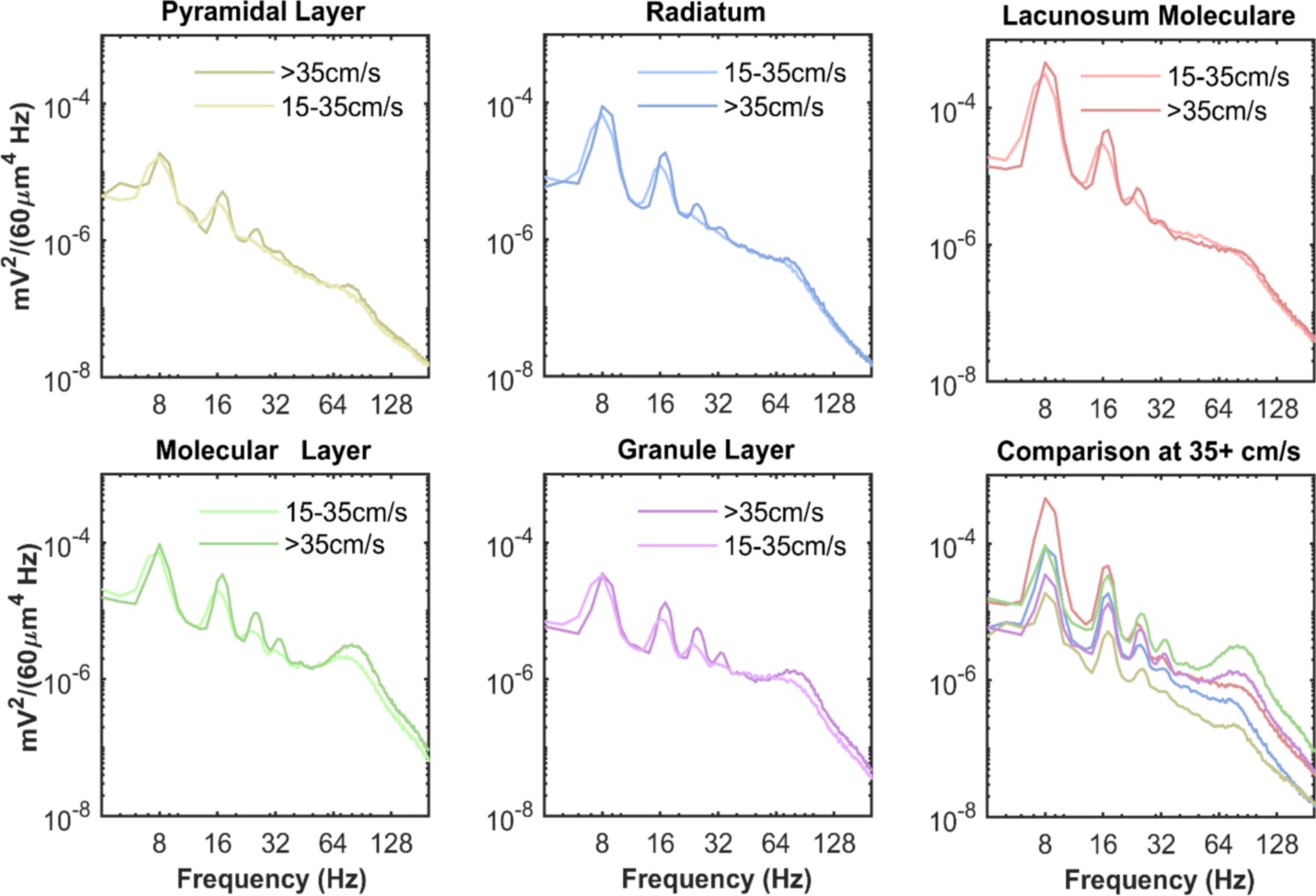


In the vast majority of our data, we observe a declining relationship in which each successive harmonic is smaller than the previous (8 Hz is larger than 16 Hz, which is larger than 24 Hz, which is larger than 32 Hz).

**6.2 The Premise Contains Multiple Unacknowledged Assumptions**

The reason this analysis was not included in the prior response is that the reviewer’s comment contains several unacknowledged assumptions that individually and collectively undermine its validity as a test.:

**“A direct comparison of harmonic power”**: This assumes harmonics can be identified from the power spectrum alone. But to know which spectral peaks are harmonics and which are independent, you need a measure of phase coupling. Power tells you energy is there; it does not tell you where it came from.

**“Testing whether putative slow-gamma components exceed the power of lower-order theta harmonics at 16 and 24 Hz”**: This treats 16 and 24 Hz as unambiguously “theta harmonics” while treating energy at 32 or 40 Hz as “putative slow gamma.” But 24 Hz (and as seen above, 32 Hz) is inside the 20–50 Hz band the field calls slow gamma. The reviewer has drawn an arbitrary frequency line where harmonics end and slow gamma begins. This is the question at issue, not a premise one can assume.

**“If slow gamma reflects a higher-order harmonic”**: This assumes slow gamma has its own ontological status and the question is whether it is “contaminated” by harmonics. The autocorrelation and ISI analyses presented in the current manuscript, along with our prior analyses failing to resolve slow gamma (but replicating the analytical artifacts that give the impression of their existence), suggest that there is no “slow gamma” to be separated. The energy in that band is harmonics plus background. The reviewer’s framing presupposes the answer, smuggling the two-gamma framework into the premise.

**“It would not be expected to carry more energy”**: Expected under what model? The Abreu geometric decay (1/n^k)? That is one parameterization. Monotonic harmonic decline requires a specific relationship between the waveform shape and its Fourier decomposition. The Abreu 1/n^k geometric series enforces it. Real physical systems (with resonant media, multiple generators, and complex waveform shapes) are not so constrained. Symmetric nonlinearities produce dominant odd harmonics (square waves). A waveform with sharp crests and flat troughs (like cnoidal waves), with broken half-wave symmetry, will produce both even and odd harmonics. The relative amplitudes depend on the specific shape of the nonlinearity. There is no universal expectation.

**“Than preceding harmonics”** : This assumes harmonic order maps simply onto frequency and that the comparison between adjacent orders is the relevant test. But the relationship between harmonic amplitudes depends on the specific shape of the nonlinearity, not on ordinal position.


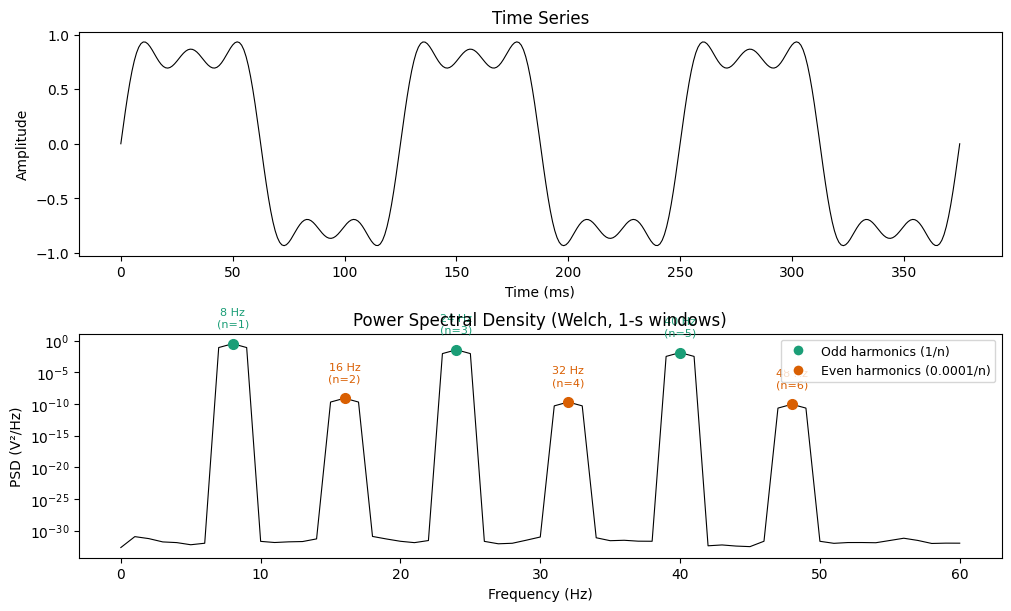
**6.3 Why We Decline to Include This Analysis**

Do we agree that this provides a complementary test? No. At best, the analysis request is an appeal for us to acquiesce to the point that an observer can say “both can be true,” which is a concession to our overall argument. A harmonic is by definition “integer phase coupled,” and there is no reason to believe hippocampal neurons are modulated by either harmonics or slow gamma (Fig. 8). By including the suggested analysis, it places us in a position of condoning a position that misrepresents our argument.

**7. Convergent Evidence from Independent Methodological Frameworks**

We agree with the reviewer that simpler, mechanistically interpretable tests strengthen the argument, which is why we have emphasized the ISI/autocorrelogram analysis (Figure 8) as a complementary, non-spectral falsification test. Together, the spike-timing analysis (showing no slow-gamma timescale in neuronal firing) and the bicoherence analysis (showing stable phase coupling between theta and putative slow-gamma components) provide convergent evidence from independent methodological frameworks.

**Regarding the explanation of the "Kemere paradox," I find the proposed mechanism difficult to reconcile with the expected spectral consequences of increased theta asymmetry. If slow-gamma-range power is largely attributable to theta harmonics, then increasing theta asymmetry (and amplitude) would generally be expected to enhance harmonic structure, including components within the 20-50 Hz range. While it is possible that energy becomes more concentrated into narrower harmonic peaks or redistributed outside this band, it is not immediately clear that such a process would produce a net decrease in band-limited power under physiologically plausible conditions. To examine this directly, I simulated myself asymmetric theta waveforms (Abreu et al., 2010). In this framework, increasing asymmetry via faster rise times (consistent with hippocampal theta) redistributes energy into higher harmonics and, when total energy is controlled, tends to increase rather than decrease power in the 20-50 Hz range. Achieving a net decrease in this band required waveform shapes with very sharp, non-physiological features (e.g., near-sawtooth profiles), suggesting that the proposed explanation may not be sufficient on its own. Taken together, this raises the possibility that the observed decrease in 20-50 Hz power with running speed cannot be explained solely by increased theta asymmetry. If the authors propose that asymmetry alone is sufficient, it would be helpful to demonstrate this explicitly, for example using a physiologically constrained waveform model of theta asymmetry.**

**1.1 Reviewer’s Original Comment**

*“In this context, it would also be relevant to understand how the authors interpret claims that slow gamma power decreases with running speed (Kemere et al., 2013, Fig. 3c), while theta nonlinearity is known to increase with speed (e.g., PMID: 27076421).”*

**1.2 Our Original Response**

*We agree that reports of slow-gamma power decreasing with speed (Kemere et al., 2013) must be interpreted carefully. This observation presents a paradox for parcellation models: how can a signal (“slow gamma”) whose power weakens with increased behavioral drive simultaneously serve as the dominant predictor of place cell spiking? In the Energy Cascade framework, this is resolved by waveform dynamics. As running speed increases, the theta waveform becomes more asymmetric (Sheremet et al., 2016). This redistributes energy across the spectrum, “tightening” the harmonic structure and paradoxically reducing power in the broad 20–50 Hz inter-harmonic trough, even as the system becomes more energetic.*

And in the manuscript:

*“Cholinergic modulation clearly shapes circuit dynamics, but the cascade view predicts that increased energetic drive and increased entrainment will reshape the PSD in a structured way, including changes that appear as selective reductions in intermediate bands, even without a dedicated routing mechanism.”*

**1.3 Clarification of Ambiguity**

We acknowledge that our previous response was ambiguous in a way that invited the reviewer’s simulation. We should clarify that our use of “waveform dynamics” drew on terminology from wave physics, where it refers to the evolution of the entire spectral field, the collective statistical reorganization of energy across scales through nonlinear coupling. In neuroscience usage, “waveform” typically refers to the shape of an individual oscillation cycle, and our statement that “the theta waveform becomes more asymmetric” was reasonably interpreted by the reviewer as a claim about single-cycle morphology. We should have been more precise: the mechanism we described is not a property of individual theta cycles but of the network-level spectral reorganization that accompanies increasing entrainment, as characterized in Sheremet et al. (2019). We restate the explanation below at the appropriate level of description.

**2. What the Reviewer’s Abreu Simulation Actually Shows**

**2.1 The Reviewer’s Comment**

*“Regarding the explanation of the “Kemere paradox,” I find the proposed mechanism difficult to reconcile with the expected spectral consequences of increased theta asymmetry. If slow-gamma-range power is largely attributable to theta harmonics, then increasing theta asymmetry (and amplitude) would generally be expected to enhance harmonic structure, including components within the 20–50 Hz range. While it is possible that energy becomes more concentrated into narrower harmonic peaks or redistributed outside this band, it is not immediately clear that such a process would produce a net decrease in band-limited power under physiologically plausible conditions. To examine this directly, I simulated myself asymmetric theta waveforms (Abreu et al., 2010). In this framework, increasing asymmetry via faster rise times (consistent with hippocampal theta) redistributes energy into higher harmonics and, when total energy is controlled, tends to increase rather than decrease power in the 20–50 Hz range. Achieving a net decrease in this band required waveform shapes with very sharp, non-physiological features (e.g., near-sawtooth profiles), suggesting that the proposed explanation may not be sufficient on its own.”*

**2.2 The Simulation Confirms Our Central Claim**

Although we do not have the simulation, **we believe the reviewer’s simulation correctly demonstrates that increasing the asymmetry of a single waveform does not reduce 20–50 Hz band power. It also nicely shows how phase coupling and nonlinearity, the same variables bicoherence measures, change wave shape and give rise to harmonics.**

The Abreu formulation constructs asymmetric waveforms from a harmonic series with explicit phase coupling between components. Specifically, it generalizes Drake and Calantoni (2001), Equation 5:

$$u\left( t \right)\propto\Sigma\left( \frac{1}{n^{k}} \right)\sin\left[ \left( k+1 \right)\omega t + k\varphi\right]$$

**The parameter φ in this expression is the biphase** (as it exists in higher-order spectral analyses such as bicoherence). Abreu et al. identify it as such, citing Elgar and Guza (1985) and Leykin et al. (1995). The parameter r (derived from n) controls the distribution of energy across harmonics. The waveform is therefore harmonically coupled by construction: every spectral component at (k+1)ω maintains a fixed phase relationship with the fundamental, governed by φ. This is not incidental to the model; it is the literal model.

This has a direct and clarifying implication. When the reviewer computed the Fourier spectrum of their simulated waveform and observed energy in the 20–50 Hz range, that energy consists entirely of phase-coupled theta harmonics. There is no mechanism in the Abreu formulation by which independent spectral energy could appear at those frequencies. It is not stated whether the reviewer found harmonics or slow gamma. However, by explicit construction of the model they chose, it is harmonics generated by nonlinear waveform asymmetry, which is the claim of our paper.

**2.3 Connection to Bicoherence (Cross-Reference)**

The biphase φ in the Abreu formulation is the same quantity that bicoherence measures: $\psi\left( f^{1}, f^{2} \right)= \varphi\left( f^{1} \right)+ \varphi\left( f^{2} \right)- \varphi\left( f^{1}+f^{2} \right)$. When bicoherence is significantly non-zero at a bifrequency pair, it indicates that the biphase is consistent across analysis windows. That is, the relationship between the fundamental and its harmonics is maintained in the data just as φ maintains it in the Abreu waveform. The reviewer’s simulation and our bicoherence analysis test the same physical relationship from opposite directions: the simulation assumes phase coupling and generates the waveform; bicoherence measures the waveform and detects the phase coupling. That both approaches point to theta harmonics in the 20–50 Hz range is convergent evidence. (For the full mathematical proof that the Abreu parameter φ is identically the biphase, see our response to the bicoherence comment, Section 2.3.)

**3. Why the Abreu Simulation Cannot Test the Mechanism We Described**

The reviewer’s Abreu et al. (2010) simulation cannot test the mechanism we described because it generates a single deterministic waveform, not a population of oscillators with distributed frequencies undergoing entrainment. A single waveform has no frequency distribution, no coupling between oscillators, no critical threshold, and no frequency-pulling. It therefore cannot exhibit the erosion phenomenon, the dips flanking the spectral peak, that is the signature of population-level entrainment.

The reviewer’s finding that increasing asymmetry in a single waveform increases harmonic power is expected and correct for that level of description. But it is analogous to studying one molecule and concluding that phase transitions do not occur: the phenomenon is collective and emerges only from the interaction of many oscillators with different natural frequencies. The Kemere paradox, decreasing 20–50 Hz power despite increasing theta power, is a population-level observation that requires a population-level explanation.

**4. The Energy Cascade Framework: A Population-Level Explanation**

**4.1 From Background to Entrainment**

The energy cascade framework (Sheremet et al., 2019) provides a principled account of the spectral redistribution underlying these observations. At low running speeds, the hippocampal LFP exhibits a featureless, approximately Gaussian, power-law background spectrum (f^−α, α ≈ 2 in CA1), consistent with a self-organized critical state (Sheremet et al., 2019, Figures 3 and 7). Energy is distributed broadly across all frequencies, including the 20–50 Hz range. As speed increases, energy enters the system at the theta scale and cascades toward higher frequencies through nonlinear triadic interactions, the same interactions bicoherence quantifies. This cascade produces two concurrent effects visible in the spectral evolution (Sheremet et al., 2019, Figure 4): theta and its harmonics emerge as discrete spectral peaks, concentrating energy at specific frequencies, while the spectral slope decreases (from α ≈ 2 toward α ≈ 1.5), reflecting the redistribution of energy from the SOC background into the cascade. In the gamma range, a spectral “front” propagates toward higher frequencies, developing a peak near 80–100 Hz consistent with a bottleneck in the cross-scale energy flux at the mesoscale/microscale transition.

**4.2 What Happens in the 20–50 Hz Window**

Consider what the hippocampal LFP looks like at rest versus during fast running. At rest or low speed, the LFP is noisy and irregular. The power spectrum is relatively smooth; energy is spread broadly across frequencies with no strong preferred rhythm. There is power in the 20–50 Hz range (indeed power at all frequencies following an approximate Amplitude = 1/frequency distribution), but it is not organized into any particular pattern. It is, loosely speaking, unentrained neural activity produced by the weakly correlated firing of many neurons.

As the animal begins to run, two things happen simultaneously. First, theta emerges and strengthens. Neurons that were firing at variable rates become entrained to theta. Their activity becomes rhythmic and coordinated. As this entrainment strengthens, the theta peak in the spectrum sharpens, and harmonics appear at 16, 24, 32, 40 Hz, and so on. These harmonics are discrete, narrow peaks. Second, interneuron networks become more strongly driven. As Traub et al. (1996) showed, gamma oscillation frequency increases linearly with driving current to interneurons. At low drive, interneurons oscillate near 25–30 Hz (within the 20–50 Hz band). At high drive, they oscillate at 60–80 Hz (above it). Gamma is a continuum, not discrete channels, as supported by Douchamps et al. (PMID: 38418832; “In conclusion, we found no evidence for narrow gamma bands”).

Now consider what happens to power measured in a fixed 20–50 Hz window during this transition. At low speed, the window captures broadband background energy from uncoordinated neural activity. At high speed, that broadband energy is attenuated as the neurons that were producing it are now entrained to both theta (strongly) and gamma (weakly), concentrating their energy at specific frequencies rather than spreading it across the spectrum. What remains in the 20–50 Hz band are the discrete theta harmonics at 24, 32, and 40 Hz, separated by gaps where there is very little energy. Between the harmonics and gamma may lose power due to this precise reorganization. If the highest harmonic was 24 Hz, as appropriately measured using bicoherence, and gamma started at 60 Hz, the pull of the organization into theta and the gamma attractors will create an erosion of power in the 25-50 Hz band as cells are force to entrain through excitation. It is a simple extension of the Wiener-Strogatz model. In their model, allowing neurons to interact with each other, a general frequency of entrainment is selected, accompanied by accretion of power in one band with erosion of power in the adjacent bands (Wiener, 1965, 1966; Strogatz, 1994). In our simple extension, neurons are heavily entrained to the macro scale (theta) and the local dynamics (gamma, 50-120 Hz), with the power spectra potentially eroding at 25-50 Hz.

Again, if this frequency is lost with drive, then what active process exists to maintain it? Kemere et al. (2013) proposed that the medial septum suppresses CA3 inputs to CA1 via cholinergic modulation during fast running, which is why slow gamma power decreases with speed. Who controls the septal state and why?

And then follow the logic.

Fernández-Ruiz et al. (2023): "the dynamic interplay between both gamma frequency inputs determines the precise timing of action potential discharge of CA1 pyramidal cells." Spike timing precision depends on the interaction between slow and mid gamma. Kemere et al. (2013): slow gamma power decreases with running speed.

Therefore, if the interplay between gamma inputs determines spike timing, and one of those inputs weakens during fast running, spike timing precision should degrade as the animal runs faster. But the opposite happens. Place fields sharpen with running speed. Phase precession tightens. Theta sequences become more organized (Maurer et al., 2012).

Follow it all the way down.

The reviewer started with their initial comment: “**that band-limited activity can serve as a proxy for pathway engagement.”** As if the utility of different frequencies **is necessary to determine the input.** CA3 doesn't shut off during fast running. CA3 place cells fire during fast running. CA3 still projects to CA1 radiatum. The anatomy hasn't changed. The pathway is active. CA1 place fields persist.

But in this Kemere discussion, slow gamma, the supposed proxy for CA3-CA1 engagement, decreases. So either CA3 is communicating with CA1 without slow gamma, in which case slow gamma was never a reliable proxy for that communication. Or CA3 is not communicating with CA1 during fast running, in which case CA1 place fields should degrade. They don't.

The proxy interpretation requires that slow gamma tracks CA3 engagement. Kemere shows slow gamma decreases during fast running. But CA3 is still engaged. The neurons fire, the anatomy is intact, the spatial coding works. The proxy fails precisely during the behavioral condition where hippocampal function is most robust.

**4.3 An Analogy**

An analogy may be helpful (noting that it is a heuristic and has no resemblance to an energy cascade). Imagine listening to an orchestra warming up. Before the conductor begins, every musician is playing fragments independently. The sound is broad and noisy, with energy at every frequency. When the conductor raises the baton and the piece begins, the sound becomes organized: strong energy at the musical pitches being played, silence between them. If you measured total acoustic power in a fixed frequency band (say, 440–880 Hz) before and after the downbeat, you might find it increased (if many notes fall in that range) or decreased (if the notes fall elsewhere and the background noise they replaced was substantial). The measurement is ambiguous not because the music is ambiguous, but because “total power in a fixed band” is the wrong measure of whether the orchestra is playing. The right measure is whether the frequencies present are harmonically related; that is, whether they are being produced by a coordinated source. That is what our analysis tests.

We therefore view the Kemere et al. (2013) observation not as a paradox but as a predictable consequence of spectral reorganization during the transition from weakly coordinated background activity to strongly entrained rhythmic activity. As neurons become more tightly coordinated, energy moves out of the broad, featureless background and into discrete, organized spectral features. The 20–50 Hz band sits in a region that loses its background energy while gaining only the theta harmonics that happen to fall within its borders. The net change in band power is indeterminate without knowing the spectral resolution of the measurement, but the phase-coupling structure of the energy that remains is unambiguous.

**5. The Literature on 20–50 Hz Power and Running Speed Is Contradictory**

It is worth noting that we are considering the veracity of a single result that contrasts with others. Chen et al. (2011; PMID: 21731735) reported that 20–45 Hz power increases with running speed. Kemere et al. (2013) reported a decrease. Zheng et al. (2015; PMID: 26774162; Figure S4) found minimal change. Ahmed and Mehta (2012) proposed that there is a single gamma frequency that shifts upward with speed, producing apparent redistribution.

This inconsistency is expected for a measure that conflates broadband background energy, theta harmonic structure, and shifting gamma frequencies within a fixed spectral window. Additionally, the general proposition—that “slow gamma” is coupled to theta, yet as theta power increases, “slow gamma” power decreases—is internally difficult to reconcile within the parcellation framework. How can a signal whose power weakens with increased behavioral drive simultaneously serve as the dominant predictor of place cell spiking?

**6. Revised Manuscript Text**

*We have revised the manuscript as follows:*

This framing provides an explanation for state-dependent spectral changes without invoking an external frequency-selective controller. For example, Kemere et al. (2013) report that power in the 20–50 Hz range decreases during running, a finding they attribute to cholinergic suppression of CA3 inputs by the medial septum. However, observations of the spectral evolution of hippocampal LFP across running speeds reveal a simpler account (Sheremet et al., 2019). At low speeds, the LFP spectrum approximates a featureless power-law background (e.g., an approximate relationship where Amplitude = 1/f), consistent with a self-organized critical state in which neural activity is weakly correlated and energy is distributed broadly across all frequencies. As running speed increases, neurons become progressively entrained to theta, concentrating spectral energy into the theta peak and discrete harmonics while depleting the broad background that previously filled the inter-harmonic frequencies. Concurrently, interneuron-network oscillation frequency increases with excitatory drive, shifting gamma-range energy from ~25–30 Hz toward ~60–80 Hz as drive strengthens (Traub et al., 1996). The 20–50 Hz range is thus caught between these two reorganizing processes (theta and gamma in this instance): it loses its broadband background energy as neurons entrain to organized rhythms (neurons are modulated by both the slow theta and the faster gamma rhythms; Zhou et al., 2019). The net result, an apparent decrease in 20–50 Hz band power during fast running, is a predictable consequence of spectral reorganization under the energy cascade (Sheremet et al., 2019), not evidence for a frequency-selective suppression mechanism.

This is a simple extension of the Wiener-Strogatz model. In their model, allowing neurons to interact with each other, a general frequency of entrainment is selected, accompanied by accretion of power in one band with erosion of power in the adjacent bands (Wiener, 1965, 1966; Strogatz, 1994). In our extension, neurons are heavily entrained to the macro scale (theta) and the local dynamics (gamma, 50-120 Hz), with the power spectra potentially eroding at 25-50 Hz.

It is worth noting that Kemere et al. (2013) report a decrease in slow gamma with velocity. This observation creates additional difficulties for the spectral parcellation framework. If the interplay between gamma frequency inputs determines spike timing precision (Fernández-Ruiz et al., 2023), then decreasing slow gamma power during fast running should degrade that precision. The opposite is observed: place fields sharpen, phase precession tightens, and theta sequences become more organized with increasing running speed (Maurer et al., 2012). Furthermore, the Kemere et al. result undermines the proxy interpretation of slow gamma. CA3 neurons remain active during fast running, the CA3-CA1 anatomical projection is intact, and CA1 spatial coding improves. Yet the signal supposedly indexing CA3-CA1 engagement weakens. Slow gamma cannot serve as a reliable proxy for CA3-CA1 communication if it fails during the behavioral condition where that pathway is most functionally relevant.

Cholinergic modulation clearly shapes circuit dynamics, but the cascade framework predicts that increased energetic drive and increased entrainment will reshape the power spectral density in a structured way, including changes that appear as selective reductions in intermediate bands, even without a dedicated routing mechanism. Notably, reports of 20–50 Hz power changes with running speed are contradictory across laboratories - increasing (Chen et al., 2011), decreasing (Kemere et al., 2013), or unchanged (Zheng et al., 2015) - as expected for a measure that conflates broadband background energy, theta harmonic structure, and shifting gamma frequencies within a fixed spectral window. Ahmed and Mehta (2012) proposed that a single gamma frequency shifts continuously upward with running speed, consistent with the cascade prediction and inconsistent with discrete, pathway-specific gamma bands.

**The passage beginning "This divergence illustrates a broader vulnerability noted by Buzsáki (2020)…" gives the impression that the specific critique (flexible selection of frequency bands and their mapping onto anatomical pathways) is directly mentioned in the cited article. However, Buzsáki (2020) does not make this argument in those terms, but rather presents a broader critique of top-down mappings between psychological constructs and neural correlates. As written, the text risks attributing a more specific claim to Buzsáki (2020) than is explicitly stated. It would be helpful to clarify that the extension to what might be termed "spectral phrenology" reflects the authors' interpretation rather than a position directly advanced in the cited work.**We agree that the attribution should be clearer. We have revised the text to specify that Buzsáki (2020) critiques top-down mappings between psychological constructs and neural correlates more broadly, and that the application to flexible frequency-band selection is our extension of this principle rather than a claim directly advanced in that work. 

Accept Letter

Dear Ms Besosa,   
 
Thank you for submitting your manuscript to Current Research in Neurobiology.

I am pleased to inform you that your manuscript has been accepted for publication. Congratulations.

Guest Editor comments are below.
    
Your accepted manuscript will now be transferred to our production department. We will create a proof which you will be asked to check, and you will also be asked to complete a number of online forms required for publication. If we need additional information from you during the production process, we will contact you directly.

We appreciate and value your contribution to Current Research in Neurobiology. We regularly invite authors of recently published manuscript to participate in the peer review process. If you were not already part of the journal’s reviewer pool, you have now been added to it. We look forward to your continued participation in our journal, and we hope you will consider us again for future submissions.

*CRNEUR* aims to be a unique, community-led journal, as highlighted in the [Editorial Introduction](https://www.sciencedirect.com/science/article/pii/S2665945X21000012). As part of this vision, we will be regularly seeking input from the scientific community and encourage you and your co-authors to take the [survey](https://www.surveymonkey.co.uk/r/5LHWTML).

We would also like to invite you to take part in our CRNEUR Author [Question & Answer (Q&A)](https://www.elsevier.com/__data/promis_misc/QA_Authors.docx), which could get published alongside your article and help to promote it. We suspect you might have an interesting story of perseverance or team work that was required for the research study to complete, or a diversity of perspectives that you might share, as a way of inspiring others about neuroscience.

Kind regards,

Anna S Mitchell, Ph.D.  
Editor in Chief  
Current Research in Neurobiology

Guest Editor comments:

Dear Cristina and colleagues,

It has been a real pleasure handling your manuscript, “Spectral Dependence as a Framework for Neural Coordination.” I am very pleased to accept your manuscript for publication. I have genuinely enjoyed following its evolution through the review process and have learned a great deal from the exchange between you and the reviewers.

The manuscript has benefited substantially from the depth and rigor of the discussions across both rounds of revision. The current version presents a much clearer and more accessible articulation of the spectral dependence framework, while engaging thoughtfully with alternative perspectives in the literature.

More broadly, I think this review process reflects the true spirit of science, where dissenting viewpoints are grounded in concrete arguments and careful analysis. While differences in interpretation remain, these are part of an active and important dialogue in the field.

As a note, Current Research in Neurobiology follows an open peer review policy, under which reviewer reports and author responses are published alongside accepted manuscripts. Accordingly, the peer review file for this manuscript will be made available upon publication. Given how thoughtful and substantive the discussion has been, this exchange may be particularly valuable to readers. Please feel free to let us know if you have any concerns.

Thank you again for the care and effort you have put into this work.

Sincerely,
Ipshita

Guest Editor for the VSI

*-------- End of Review Comments --------*
